# Supplementary material for: Vulnerability of invasive glioblastoma cells to lysosomal membrane destabilization
Source: EMBO Mol Med. 2019 May 8;11(6):e9034. doi: 10.15252/emmm.201809034 (PMC6554674; doi:10.15252/emmm.201809034)
Supplement: Supplementary file 1 — Appendix [file EMMM-11-e9034-s001.pdf]

Appendix

VULNERABILITY OF INVASIVE GLIOBLASTOMA CELLS TO LYSOSOMAL MEMBRANE DESTALIBISATION

Vadim Le Joncour,[1*] Pauliina Filppu,[1*] Maija Hyvönen,[1] Minna Holopainen,[2] S. Pauliina Turunen,[3,4] Harri Sihto,[1] Isabel Burghardt,[5] Heikki Joensuu,[1,6] Olli Tynninen,[7] Juha Jääskeläinen,[8] Michael Weller,[5] Kaisa Lehti,[3,4] Reijo T. Käkelä[2] and Pirjo Laakkonen[1,9]

Table of contents

Appendix Materials and Methods…………………………………………………………………...2

Antibodies……………………………………………………………………………..2

BAD silencing………………………………………………………………………....2

Appendix Figure S1……………………………………………………………………………..3

Appendix Figure S2……………………………………………………………………………..6

Appendix Figure S3……………………………………………………………………………..8

Appendix Figure S4…………………………………………………………………………...10

Appendix Table S1………………………………………………………………………………12

Appendix Table S2………………………………………………………………………………13

Appendix Table S3………………………………………………………………………………14

Appendix Material and methods

Antibodies

Imaging: mouse monoclonal anti-MDGI (sc-58274) was obtained from Santa Cruz Biotechnology, rat monoclonal antibody against MDGI (MAB1678) from R&D Systems, rabbit polyclonal antibodies against LGALS1 (ab25138) and LAMP2 (ab25631) from Abcam, human vimentin-Cy3 (C9080) from Sigma and rat anti-mouse CD31 (553370) from BD Pharmingen. **Western blot:** Rabbit polyclonal antibodies against HIF1α (#3716), β-actin (#4967), phosphorylated (Thr202/Tyr204) extracellular signal-regulated kinase 1/2 (ERK1/2, #9106), AKT (#9272), phosphorylated (Thr308) AKT (#9275), caspase 3 (8G10, #9665) and phosphorylated (Ser15) P53 (#9284) were obtained from Cell Signaling, rat monoclonal antibody against MDGI (MAB1678) from R&D Systems, mouse monoclonal anti-GAPDH (UBG8140) from Europa Bioproducts, rabbit polyclonal antibodies against EGFR (sc-03), anti-P27 (sc-528), ERK1 (sc-94), and mouse monoclonal antibodies against P53 (sc-126) and BAD (sc-8044) from Santa Cruz Biotechnology, and mouse monoclonal anti-β-tubulin (556321) from BD Pharmingen.

Silencing of BAD

To study whether silencing of BAD could rescue the MDGI silencing induced cell death, BT12 and BT13 cells were transfected with control or BAD siRNAs and after 48 hrs transduced with lentiviruses encoding control, scrambled (Scr) or MDGI shRNA (shM1). Cell number was measured using the MTT assay 1, 2, 3 and 4 days after transduction.

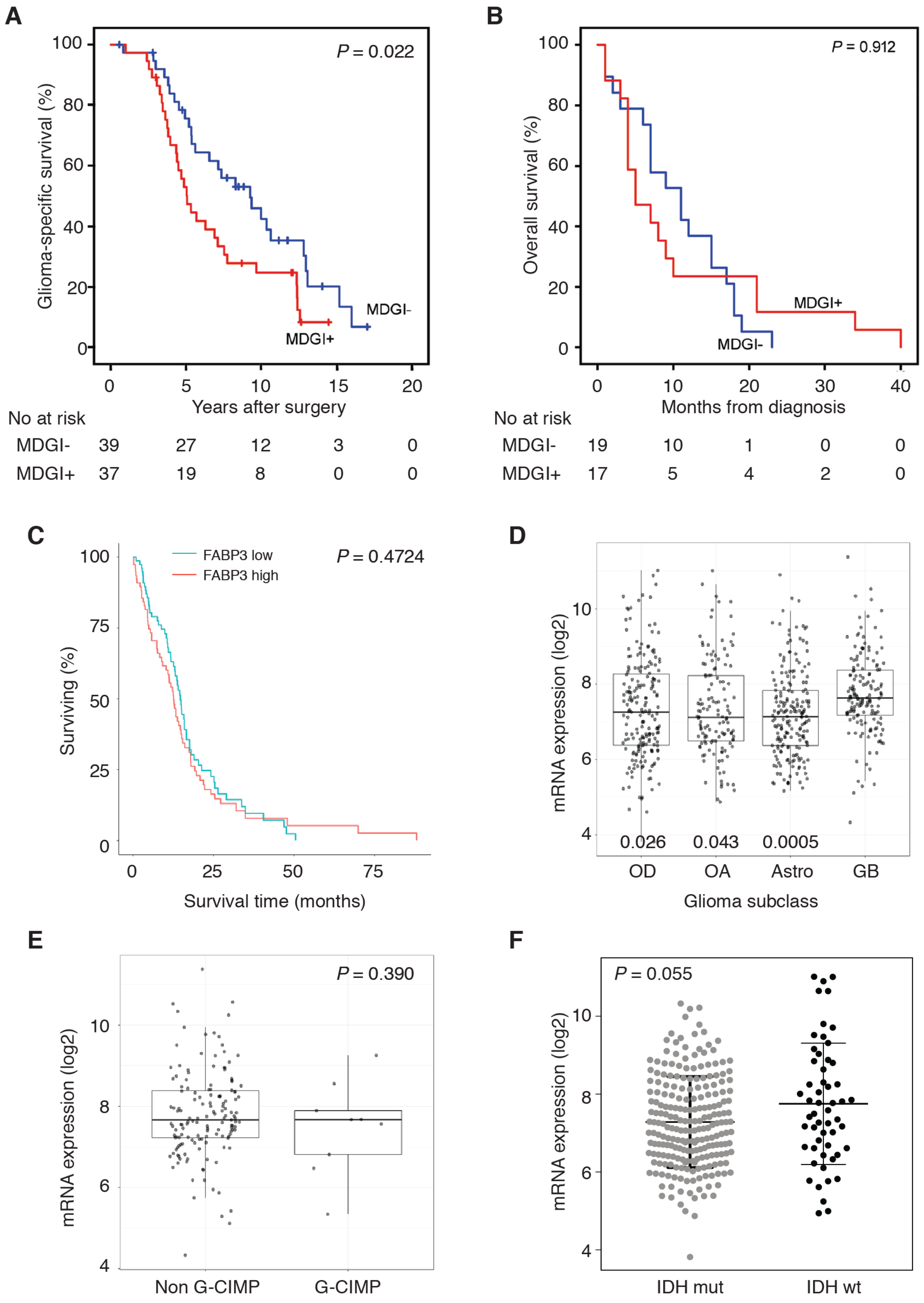
A
P = 0.022
Glioma-specific survival (%)
100
80
60
40
20
0
MDGI-
MDGI+
0
5
10
15
20
Years after surgery
No at risk
MDGI- 39 27 12 3 0
MDGI+ 37 19 8 0 0
B
P = 0.912
Overall survival (%)
MDGI+
MDGI-
0
10
20
30
40
Months from diagnosis
No at risk
MDGI- 19 10 1 0 0
MDGI+ 17 5 4 2 0
C
FABP3 low
FABP3 high
P = 0.4724
Surviving (%)
100
75
50
25
0
Survival time (months)
D
mRNA expression (log2)
0.026
0.043
0.0005
OD
OA
Astro
GB
Glioma subclass
E
P = 0.390
mRNA expression (log2)
Non G-CIMP
G-CIMP
F
P = 0.055
mRNA expression (log2)
IDH mut
IDH wt

Appendix Figure S1

A Glioma-specific survival of patients diagnosed with grade II and III glioma (***n*** = 76) was significantly better in patients with none/low MDGI expression (blue line) compared to patients with moderate/high (red line) MDGI expression (***P*** = 0.022). The cumulative survival was estimated by using the Kaplan–Meier method.

B Overall survival did not differ between glioblastoma patients (***n*** = 36) with none/low (blue line) MDGI expression and patients with moderate/high (red line) MDGI expression (***P*** = 0.912). The cumulative survival was estimated by using the Kaplan–Meier method.

C Analysis of the association of MDGI mRNA expression with glioblastoma patient survival using the TCGA GBM dataset (***n*** = 155) in the GlioVis data portal. No significant difference was observed between patients with low (green line; ***n*** = 77, events = 59, median. = 14.7) or high (red line; ***n*** = 78, events = 64, median = 12.6) MDGI/*FABP3* expression (***P*** = 0.4724). The Kaplan-Meier method was used to estimate the cumulative survival rates.

D Analysis of MDGI expression in the different glioma histological subtypes using the GlioVis data portal (the TCGA GBMLGG dataset, ***n*** = 667). Glioblastomas express higher levels of MDGI than the lower grade gliomas. OD, oligodendroglioma,; OA, oligoastrocytoma; Astro, astrocytoma; GB, glioblastoma. Numbers in the graph depict the ***P*** values lower grade gliomas compared to glioblastomas. Pairwise t-test with corrections for multiple testing, p-values with Bonferroni correction

E Association of MDGI expression with the CIMP status in glioblastomas using the GlioVis data portal (the TCGA GBM dataset, ***n*** = 156). The majority (94%) of MDGI expressing tumours display the nonG-CIMP status. ***P*** = 0.390. Pairwise t-test with corrections for multiple testing, p-values with Bonferroni correction.

F Association of MDGI expression with the IDH mutation status in lower grade gliomas using the GlioVis data portal (the TCGA LGG dataset, ***n*** = 281). No significant difference in MDGI expression was observed between the IDH wt and mutant tumours. ***P* =** 0.055, two-tailed, nonparametric Mann-Whitney's *U*-test.

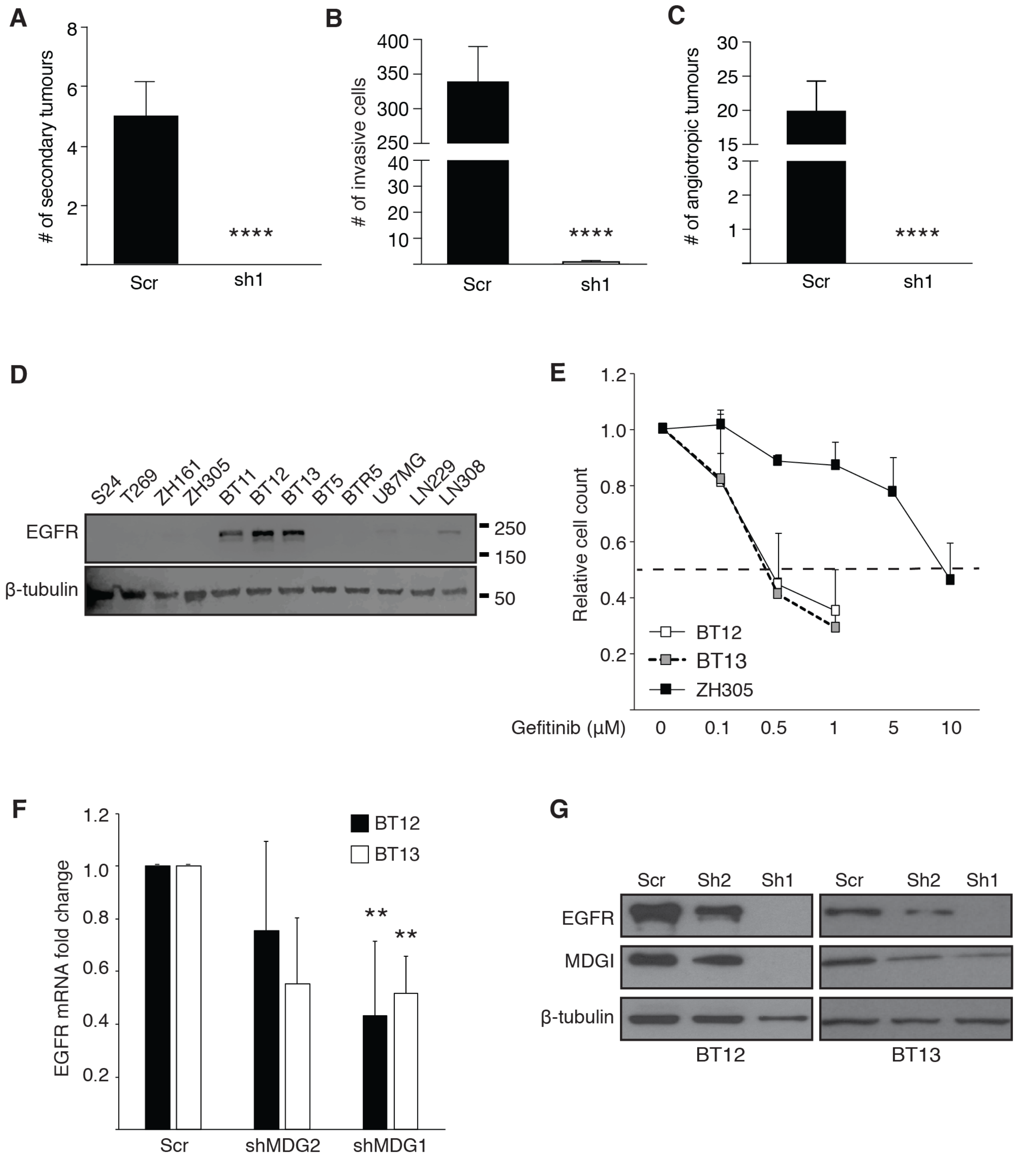

Appendix Figure S2

A, B, C Quantification of the number of secondary tumours (diameter >300 μm), number of invasive tumour cells, and number of angiotropic tumours detected in the whole brain of mice injected intracranially with control (Scr, $n = 5$) or MDGI-silenced (sh1, $n = 5$) BT12 cells. Data are represented as mean ± SD. ****$P < 0.0001$, two-tailed, nonparametric Mann-Whitney's U-test.

D Western blot analysis demonstrating expression of EGFR in various human glioma cell lines. β-tubulin served as a loading control.

E EGFR inhibition in BT12, BT13, and ZH305 glioblastoma cells by gefitinib. The number of viable cells was determined using an MTT assay 48h after the treatment and normalized to the untreated controls ($\boldsymbol{n}$ = 3). A dashed line represents the half maximal inhibitory concentration (IC_{50}) that was 0.4 μM for BT12 and BT13 cells that express high amounts of EGFR and 9.4 μM for ZH305 cells that express a negligible amount of EGFR.

F EGFR mRNA expression in control (Scr) and MDGI-silenced (shMDG1 and shMDG2) glioblastoma cells six days post-transduction. A representative graph of more than three individual experiments is shown. Data are represented as mean ± SD. $^{**}\boldsymbol{P} < 0.01$, two-tailed, nonparametric Mann-Whitney's *U*-test.

G Western blot analysis showing decreased expression of EGFR in response to MDGI silencing in BT12 and BT13 cell lines. β-tubulin served as a loading control.

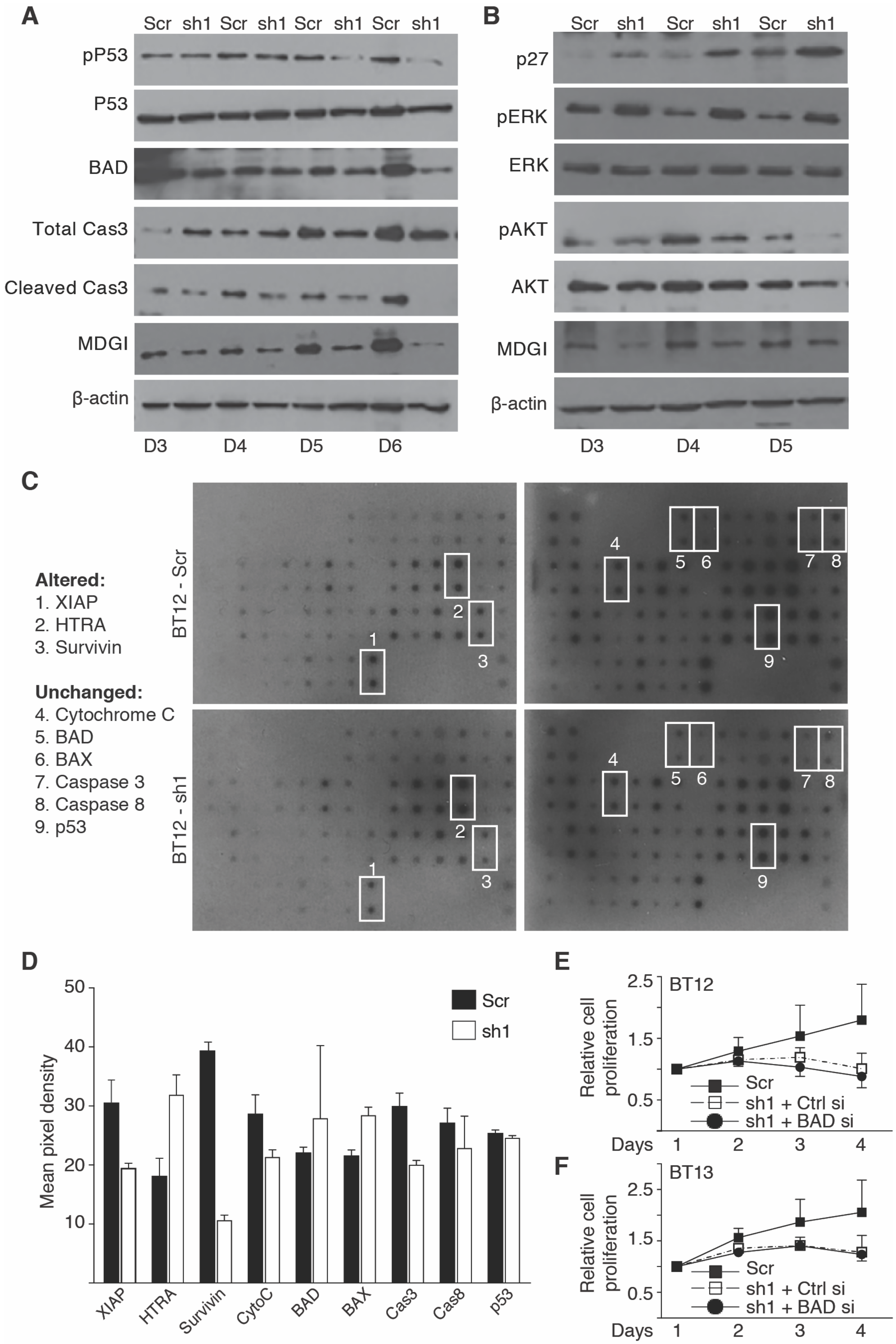
A
Scr sh1 Scr sh1 Scr sh1 Scr sh1
pP53
P53
BAD
Total Cas3
Cleaved Cas3
MDGI
β-actin
D3 D4 D5 D6
B
Scr sh1 Scr sh1 Scr sh1
p27
pERK
ERK
pAKT
AKT
MDGI
β-actin
D3 D4 D5
C
Altered:
1. XIAP
2. HTRA
3. Survivin
Unchanged:
4. Cytochrome C
5. BAD
6. BAX
7. Caspase 3
8. Caspase 8
9. p53
BT12 - Scr
BT12 - sh1
D
Mean pixel density
Scr
sh1
XIAP
HTRA
Survivin
CytoC
BAD
BAX
Cas3
Cas8
p53
E
BT12
Relative cell proliferation
Scr
sh1 + Ctrl si
sh1 + BAD si
Days
F
BT13
Relative cell proliferation
Scr
sh1 + Ctrl si
sh1 + BAD si
Days

Appendix Figure S3

A Western blot analysis shows expression of apoptosis-associated proteins in control (Scr) and MDGI-silenced (sh1) BT12 cells. Cell extracts were collected at indicated time points. Representative images of two individual experiments are shown.

B Western blot analysis shows expression of selected intracellular signalling proteins after MDGI silencing. Cell extracts were collected at indicated time points from control (Scr) and MDGI-silenced (sh1) cells. Representative images of two individual experiments are shown.

C Two different exposures of antibody-immobilized PVDF-membranes containing 43 different apoptosis-associated proteins. The membranes were incubated with extracts of control (Scr) and MDGI-silenced (sh1) BT12 cells. Differentially expressed proteins (fold change less than 0.6 or more than 1.5) are indicated with numbers 1-3 and examples of unaltered proteins with numbers 4-9.

D Graph shows the relative pixel density of each protein (n=2) of the apoptosis array shown in C four days after MDGI silencing. Analysis was performed using the ImageJ software according to manufacturer´s instructions.

E, F BT12 (E) and BT13 (F) cells were transfected with control (Ctrl si) or BAD (BAD si) siRNAs and transduced with lentiviruses encoding control, scrambled (Scr) or MDGI shRNA (sh1) 48 hrs after transfection. Cell number was measured using the MTT at indicated time points.

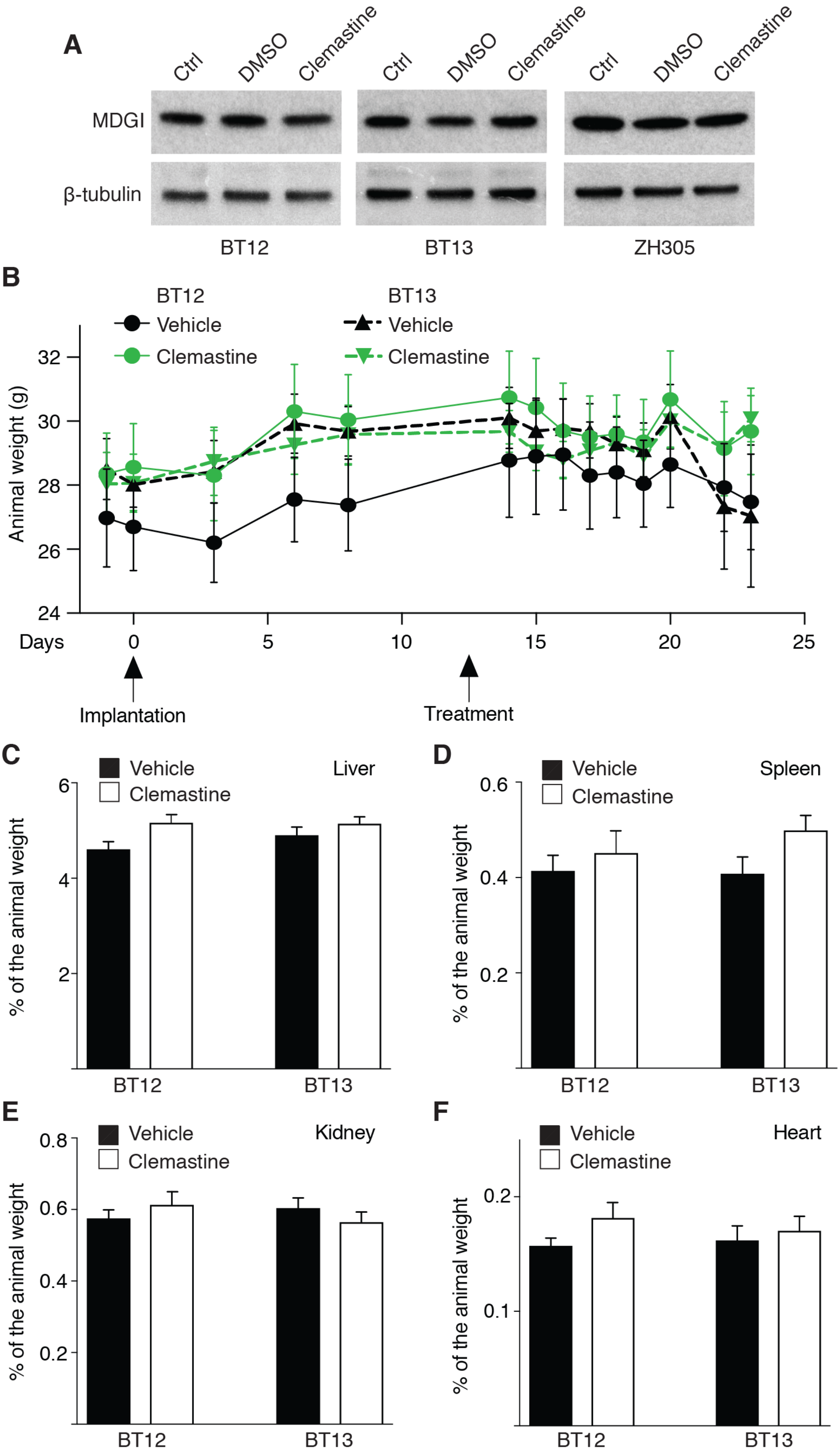
A
Ctrl
DMSO
Clemastine
MDGI
β-tubulin
BT12
BT13
ZH305
B
BT12
Vehicle
Clemastine
BT13
Vehicle
Clemastine
Animal weight (g)
Days
Implantation
Treatment
C
Vehicle
Clemastine
Liver
% of the animal weight
BT12
BT13
D
Vehicle
Clemastine
Spleen
% of the animal weight
BT12
BT13
E
Vehicle
Clemastine
Kidney
% of the animal weight
BT12
BT13
F
Vehicle
Clemastine
Heart
% of the animal weight
BT12
BT13

Appendix Figure S4

A Western blot analysis shows MDGI expression in BT12, BT13, and ZH305 cells that were treated with DMSO or 1 μM clemastine or left untreated for 24 hrs. No difference was observed in MDGI levels. β-tubulin served as a loading control.

B Graph shows the body weights of mice bearing BT12 or BT13 intracranial tumours. Tumours were implanted at day 0 and treated with saline vehicle (Vehicle, BT12 ***n*** = 4, BT13 ***n*** = 5) or 50 mg/kg clemastine (BT12 ***n*** = 5, BT13 ***n*** = 4) for 12 days starting at day 12. Arrows mark the tumour implantation and start of the treatment.

C, D, E, F Liver, spleen, kidney, and heart to body weight ratio of nude mice implanted with patient-derived BT12 or BT13 glioblastoma cells and daily treated with saline vehicle (control, BT12 ***n*** = 4, BT13 ***n*** = 5) or 50 mg/kg clemastine (BT12 ***n*** = 5, BT13 ***n*** = 4) for 12 days. Data are represented as mean ± SEM. No significance was found between the vehicle and clemastine groups. ***P***-values were calculated using two-tailed, nonparametric Mann-Whitney's *U*-test.

Appendix Table S1. Association of MDGI expression with clinical and histopathological factors in glioma.

Factor	MDGI 0+1 N= 39 (%)	MDGI 2+3 N= 37 (%)	P (Chi2)
**Gender**			
Female	17 (43,6)	19 (51,4)	0,498
Male	22 (56,4)	18 (48,6)	
**Grade**			
2	28 (71,8)	20 (54,1)	0,109
3	11 (28,2)	17 (45,9)	
**Histological type**			
Astrocytoma	18 (46,2)	15 (40,5)	0,296
Anaplastic astrocytoma	5 (12,8)	11 (29,7)	
Oligodendroglioma	10 (25,6)	8 (21,6)	
Oligoastrocytoma	6 (15,4)	3 (8,1)	
**MDGI vessel expression**			
Absent	10 (43,5)	3 (11,5)	0,011
Present	13 (56,5)	23 (88,5)	
Not available	16	11	
**Tumour KIT**			
Absent	34 (100)	33 (94,3)	0,493*
Present	0 (0)	2 (5,7)	
Not available	5	2	
**Tumour EGFR**			
Absent	28 (80)	34 (91,9)	0,145
Present	7 (20)	3 (8,1)	
Not available	4	0	
**Tumour p-EGFR**			
Absent	33 (100)	33 (100)	
Present	0	0	
Not available	6	4	
**Tumour p53**			
Absent	22 (62,9)	17 (45,9)	0,150
Present	13 (37,1)	20 (54,1)	
Not available	4	0	

*Fisher´s p-value

Appendix Table S2. Association of MDGI expression with clinical and histopathological factors in glioblastoma.

Factor	MDGI 0+1 N=19 (%)	MDGI 2+3 N=17 (%)	P (Fisher´s)
**Gender**			
Female	6 (31,6)	9 (52,9)	0,311
Male	13 (68,4)	8 (47,1)	
**Age**	N=39	N=37	
Min	17	23	0,92
Max	56	62	
Median	40	35	
**MDGI vessel expression**			
Absent	10 (66,7)	2 (15,4)	0,009
Present	5 (33,3)	11 (84,6)	
Not available	4	4	
**Tumour KIT** [a]			
Absent	15 (78,9)	11 (64,7)	0,463
Present	4 (21,1)	6 (35,3)	
**Perinecrotic KIT**[a]			
Absent	18 (94,7)	9 (52,9)	0,006
Present	1 (5,3)	8 (47,1)	
**Tumour EGFR**			
Absent	7 (38,9)	6 (40,0)	>0,999
Present	11 (61,1)	9 (60,0)	
Not available	1	2	
**Tumour EGFRvIII**			
Absent	16 (84,2)	14 (93,3)	0,613
Present	3 (15,8)	1 (6,7)	
Not available	0	2	
**Tumour p-EGFR**[b]			
Absent	11 (68,8)	8 (66,7)	>0,999
Present	5 (31,3)	4 (33,3)	
Not available	2	5	
**Tumour p53**			
Absent	14 (87,5)	11 (100)	0,499
Present	2 (12,5)	0 (0)	
Not available	3	6	

[a]KIT = CD117/C-Kit receptor
[b]Tyr-1173 phosphorylation

Appendix Table S3. Exact ***P*** values.

Result	Figure	*P* value	Summary
**Figures**			
Overall survival glioma	1B	0.007	**
Survival (TCGA GBMLGG)	1C	2.0e-4	***
Glioblastoma subtypes (TCGA)	1D		
Mesenchymal – Classical		0.29	ns
Proneural – Classical		0.64	ns
Proneural – Mesenchymal		1.00	ns
IVY_GAP histology	1E		
Leading edge – Cellular tumour		1.3e-18	***[a)]
Pseudopalisading cells – Microvascular proliferation		4.0e-10	***
Infiltrating tumour – Cellular tumour		8.9e-11	***
Leading edge – Infiltrating tumour		0.0035	***
Pseudopalisading cells – Cellular tumour		0.077	ns
Pseudopalisading cells – Infiltrating tumour		9.9e-5	***
Microvascular proliferation – Cellular tumour		2.5e-5	***
Pseudopalisading cells – Leading edge		8.5e-12	***
Microvascular proliferation – Infiltrating tumour		5.1e-21	***
Microvascular proliferation – Leading edge		6.7e-28	***
Number of secondary tumours	2M	0.0079	**
Number of angiotropic tumours	2N	< 0.0001	****[b)]
Number of invasive cells	2O	< 0.0001	****
Number of angiotropic tumours	2P	< 0.0001	****
Self-renewal	3C		
BT12 scr – BT12 sh2		0.1320	ns
BT12 scr – BT12 sh1		0.0002	***
BT13 scr – BT13 sh2		0.0798	ns
BT13 scr – BT13 sh1		0.0003	***
Relative cell proliferation	3D		
BT12 scr – BT12 sh1, 3d		0.0004	***
BT12 scr – BT12 sh1, 5d		< 0.0001	***[c)]
BT13 scr – BT13 sh1, 3d		0.0006	***
BT13 scr – BT13 sh1, 5d		< 0.0001	***
Annexin V	4B		
BT12 scr – BT12 sh1, P1		0.0045	**
BT12 scr – BT12 sh1, P2		0.0045	**
Puncta staining	4I		
BT12 scr – BT12 sh1		0.0022	**
BT13 scr – BT13 sh1		0.0022	**
Dead cells	4J		
BT12 scr – BT12 sh1		0.0022	**
BT13 scr – BT13 sh1		0.0022	**
Cytoplasmic cathepsin B activity	4K		
BT12 scr – BT12 sh1		0.035	*
BT13 scr – BT13 sh1		0.004	**
Relative cell viability	4L		
BT12 sh1 – BT12 sh1+K777, 1d		> 0.9999	ns
BT12 sh1 – BT12 sh1+K777, 3d		> 0.9999	ns
BT12 sh1 – BT12 sh1+K777, 5d		0.0379	*
BT13 sh1 – BT13 sh1+K777, 1d		> 0.9999	ns

BT13 sh1 – BT13 sh1+K777, 3d		> 0.9999	ns
BT13 sh1 – BT13 sh1+K777, 5d		0.0022	**
Number of invasive cells	7B		
BT12 vehicle – BT12 clemastine		< 0.0001	****
Distance of invasive cells	7C		
BT12 vehicle – BT12 clemastine		< 0.0001	****
Number of secondary tumours	7D		
BT12 wt control – BT12 wt clemastine		< 0.0001	****
BT12 scr control – BT12 scr clemastine		< 0.0001	****
BT12 shMDGI – BT12 shMDGI clemastine		> 0.9999	ns
BT13 vehicle – BT13 clemastine		> 0.9999	ns
Number of angiotropic tumours	7E		
BT12 vehicle – BT12 clemastine		< 0.0001	****
Number of TUNEL+ cells	7F		
BT12 vehicle – BT12 clemastine		< 0.0001	****
Survival	7H		
BT12 vehicle – BT12 clemastine		0.044	*
**Expanded view figures**			
Cell proliferation (U87-GFP – U87 MDGI-GFP)	EV1B	0.2836	ns
Colony formation (U87-GFP – U87 MDGI-GFP)	EV1D	0.0049	**
Invaded cells *ex vivo* (U87-GFP – U87 MDGI-GFP)	EV1F	0.006	**
Colony formation	EV2C		
BT12 scr – BT12 sh2		0.0049	**
BT12 scr – BT12 sh1		0.0049	**
BT13 scr – BT13 sh2		0.0043	**
BT13 scr – BT13 sh1		0.0022	**
Relative cell proliferation	EV2D		
BT12 scr – BT12 sh2, 3d		0.0027	**
BT12 scr – BT12 sh2, 5d		< 0.0001	***
BT13 scr – BT13 sh2, 3d		0.0006	***
BT13 scr – BT13 sh2, 5d		0.0003	***
Cell viability	EV2E		
BT12 scr – BT12 sh2		< 0.0001	***
BT12 scr – BT12 sh1		< 0.0001	***
BT13 scr – BT13 sh2		0.0008	***
BT13 scr – BT13 sh1		< 0.0001	***
Relative cell proliferation	EV2F		
ZH305 scr – ZH305 sh1, 3d		0.0004	***
ZH305 scr – ZH305 sh1, 5d		0.0004	***
Cell viability	EV2G		
ZH305 scr – ZH305 sh1		< 0.0001	***
Tumour area	EV4C		
BT12 wt vehicle – BT12 wt clemastine		> 0.9999	ns
BT12 scr vehicle – BT12 scr clemastine		> 0.9999	ns
BT13 vehicle – BT13 clemastine		> 0.9999	ns
ZH305 vehicle – ZH305 clemastine		< 0.0001	****
**Appendix Supplementary figures**			
Glioma-specific survival	S1A	0.022	*
Overall survival GBM	S1B	0.912	ns
Survival (TCGA GBM)	S1C	0.4724	ns
Histological glioma subclass (TCGA GBMLGG)	S1D		

GBM – Astrocytoma		4.9e-4	***
GBM – Oligoastrocytoma		0.043	*
GBM – Oligodendroglioma		0.026	*
Oligoastrocytoma – Oligodendroglioma		1.00	ns
Astrocytoma – Oligoastrocytoma		1.00	ns
Astrocytoma – Oligodendroglioma		1.00	ns
G-CIMP (TCGA GBM)	S1E	0.39	ns
IDH status (TCGA LGG)	S1F	0.055	ns
Number of secondary tumours	S2A	< 0.0001	****
Number of invasive cells	S2B	< 0.0001	****
Number of angiotropic tumours	S2C	< 0.0001	****
EGFR mRNA fold change	S2F		
BT12 scr – BT12 sh2		0.8884	ns
BT12 scr – BT12 sh1		0.0079	**
BT13 scr – BT13 sh2		1.0000	ns
BT13 scr – BT13 sh1		0.0079	**
BAD silencing D4	S3E		
BT12 Scr – BT12 sh1 + Ctrl si		0.0796	ns
BT12 Scr – BT12 sh1 + BAD si		0.0012	**
BT12 sh1 + Ctrl si – BT12 sh1 + BAD si		0.5550	ns
BAD silencing D4	S3F		
BT13 Scr – BT13 sh1 + Ctrl si		0.0073	**
BT13 Scr – BT13 sh1 + BAD si		0.0242	*
BT13 sh1 + Ctrl si – BT13 sh1 + BAD si		> 0.9999	ns
Liver to animal weight %	S4C		
BT12 vehicle – BT12 clemastine		0.1111	ns
BT13 vehicle – BT13 clemastine		0.2857	ns
Spleen to animal weight %	S4D		
BT12 vehicle – BT12 clemastine		0.5556	ns
BT13 vehicle – BT13 clemastine		0.0635	ns
Kidney to animal weight %	S4E		
BT12 vehicle – BT12 clemastine		0.9048	ns
BT13 vehicle – BT13 clemastine		0.4127	ns
Heart to animal weight %	S4F		
BT12 vehicle – BT12 clemastine		0.1270	ns
BT13 vehicle – BT13 clemastine		0.4127	ns

[a)] The GlioVis and Ivy_GAP dataportals mark all ***P*** values <0.001 with ***.
[b)] **** = Prism software v8 provides <0.0001 as an exact ***P*** value when the result is highly significant.
[c)] *** = Prism software v6 provides <0.0001 as an exact ***P*** value when the result is highly significant.
