# Supplementary material for: Vulnerability of invasive glioblastoma cells to lysosomal membrane destabilization
Source: EMBO Mol Med. 2019 May 8;11(6):e9034. doi: 10.15252/emmm.201809034 (PMC6554674; doi:10.15252/emmm.201809034)
Supplement: Supplementary file 2 — Expanded View Figures PDF [file EMMM-11-e9034-s002.pdf]

# Expanded View Figures

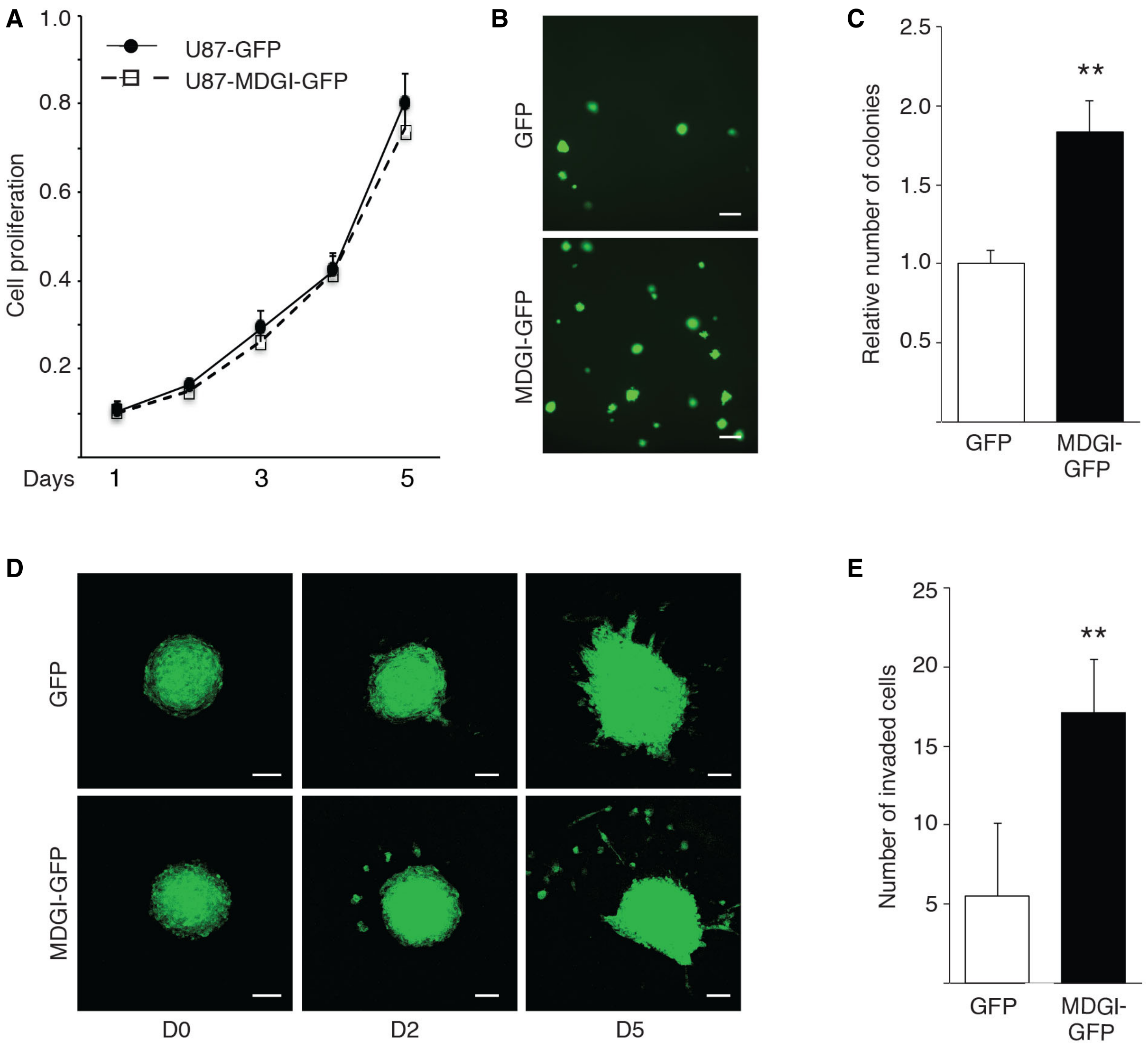


**Figure EV1. MDGI overexpression promotes glioma cell invasion.**

A Graph shows proliferation rate of U87MG-GFP control and MDGI-overexpressing U87MG-MDGI-GFP cells *in vitro*. Values of each cell line were normalized to the corresponding values at day 1 ($n = 3$). Data are represented as mean ± SD.

B Colony formation assay demonstrating anchorage-independent growth of U87MG-GFP control and MDGI-overexpressing U87MG-MDGI-GFP cells after 14 days in culture *in vitro*. Scale bar: 200 μm.

C MDGI overexpression (MDGI-GFP) significantly increased the number of the colonies compared to the GFP-expressing control cells ($n = 3$). Data are represented as mean ± SD. $^{**}P < 0.01$, two-tailed, nonparametric Mann–Whitney's *U*-test.

D Spheres of U87MG-GFP control and MDGI-overexpressing U87MG-MDGI-GFP were placed on top of murine brain slices, and their motility was recorded by confocal microscopy at the indicated time points (D0, D2 and D5, $n \geq 7$). Each micrograph is the maximal intensity projection from 10-μm-thick optical slices (18–22 slices per sample). Scale bar: 200 μm.

E Quantification of the number of invaded MDGI-GFP cells (black bar) relative to the GFP controls (white bar; $n \geq 7$). Data are represented as mean ± SD. $^{**}P < 0.01$, two-tailed, nonparametric Mann–Whitney's *U*-test.

Source data are available online for this figure.

 © 2019 The Authors

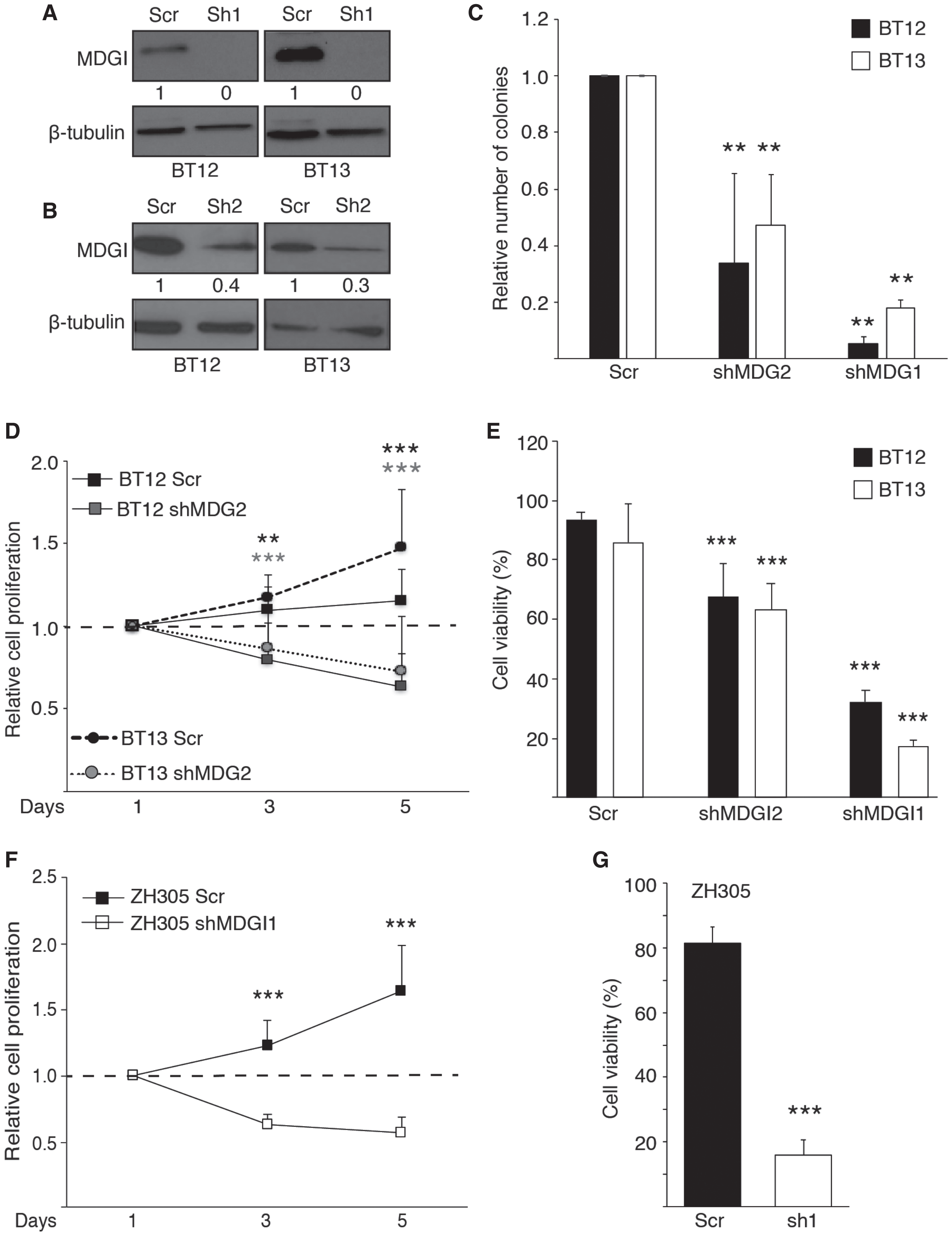
A
Scr Sh1 Scr Sh1
MDGI
1 0 1 0
β-tubulin
BT12 BT13
B
Scr Sh2 Scr Sh2
MDGI
1 0.4 1 0.3
β-tubulin
BT12 BT13
C
Relative number of colonies
BT12
BT13
Scr shMDG2 shMDG1
D
Relative cell proliferation
BT12 Scr
BT12 shMDG2
BT13 Scr
BT13 shMDG2
Days 1 3 5
E
Cell viability (%)
BT12
BT13
Scr shMDGI2 shMDGI1
F
Relative cell proliferation
ZH305 Scr
ZH305 shMDGI1
Days 1 3 5
G
ZH305
Cell viability (%)
Scr sh1

**Figure EV2.**

**Figure EV2. MDGI silencing reduces glioblastoma cell viability.**

A, B Western blot analyses of control (Scr) and MDGI-silenced patient-derived BT12 and BT13 glioblastoma cells using two different shRNAs, MDGI1 (sh1) and MDGI2 (sh2). sh1 leads to almost total loss of MDGI protein, while sh2 leads to partial silencing of MDGI compared to control cells.

C Colony formation assay demonstrating anchorage-independent growth of control (Scr) and MDGI-silenced (shMDGI2 and shMDGI1) patient-derived BT12 and BT13 cells after 27 days of culture. The number of colonies relative to the controls is indicated ($n$ = 3). Data are represented as mean ± SD. **$P < 0.01$, two-tailed, nonparametric Mann–Whitney's $U$-test.

D MTT-proliferation assay of control (Scr) and MDGI-silenced (shMDGI2) cells. Values of each cell line were normalized to the corresponding values at day 1 (dashed line) ($n$ = 3). Data are represented as mean ± SD. **$P < 0.01$; ***$P < 0.001$, two-tailed, nonparametric Mann–Whitney's $U$-test.

E Viability of BT12 and BT13 cells was determined using the Trypan blue staining 10 days after MDGI silencing (shMDGI2 and shMDGI1, $n$ = 3). Data show the percentage of live cells compared to the untreated control cells set as 100%. Data are represented as mean ± SD. ***$P < 0.001$, two-tailed, nonparametric Mann–Whitney's $U$-test.

F MTT cell proliferation assay of control (Scr) and MDGI-silenced (shMDGI1) ZH305 cells. Values of each cell line were normalized to the corresponding values at day 1 ($n$ = 3). Data are represented as mean ± SD. ***$P < 0.001$, two-tailed, nonparametric Mann–Whitney's $U$-test.

G Viability of control (Scr) and MDGI-silenced (sh1) ZH305 cells determined by Trypan blue staining at day 10 post-transduction ($n$ = 3). Data are represented as mean ± SD. ***$P < 0.001$, two-tailed, nonparametric Mann–Whitney's $U$-test.

Source data are available online for this figure.

 © 2019 The Authors

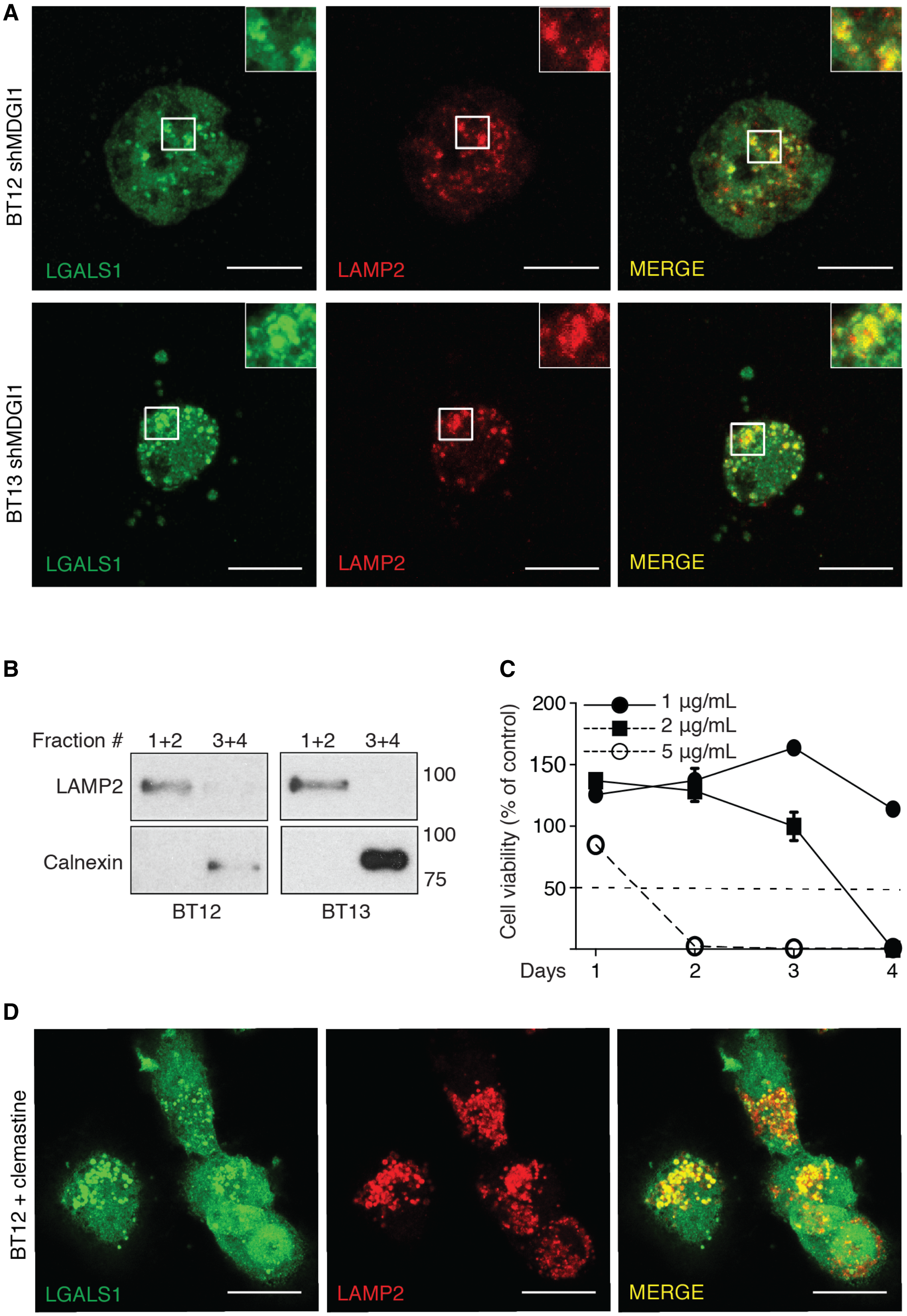
A
BT12 shMDGI1
BT13 shMDGI1
LGALS1
LAMP2
MERGE
B
Fraction #
1+2
3+4
LAMP2
Calnexin
100
75
BT12
BT13
C
1 µg/mL
2 µg/mL
5 µg/mL
Cell viability (% of control)
200
150
100
50
Days
1
2
3
4
D
BT12 + clemastine

**Figure EV3.**

**Figure EV3. MDGI silencing induces LMP.**

A MDGI-silenced (shMDGI1) BT12 and BT13 cells were stained with anti-galectin-1 (LGALS1, green) and anti-LAMP2 (red) antibodies 6 days after silencing. Co-localization of the LGALS1 with LAMP2 is seen as yellow colour (merge). Scale bar: 10 μm.

B Lysosomal extracts of BT12 and BT13 cells were prepared 6 days after MDGI silencing by density gradient ultracentrifugation. Western blot analyses show the presence of lysosomes and purity of the collected fractions (1 + 2 and 3 + 4). Antibodies against LAMP2 and Calnexin-1 were used for the localization of lysosomes (fractions 1 + 2) and endoplasmic reticulum (fractions 3 + 4), respectively.

C Measurement of the viability of murine brain endothelial (bEND3) cells using MTT assay at the indicated clemastine concentrations and time points ($n$ = 12). A dashed line marks the 50% cell viability. Data are represented as mean ± SD.

D Co-localization of the LGALS1 (green) with the lysosomal marker protein LAMP2 (red) is seen as yellow colour (merge) in clemastine (1 μg/ml)-treated BT12 cells. Scale bar: 10 μm.

Source data are available online for this figure.

**Figure EV4. Clemastine eradicates invasive glioblastoma cells by evoking the LMP.**

A, B Representative whole coronal section micrographs of murine brain intracranially implanted with MDGI-silenced BT12 glioblastoma cells, and daily treated with saline vehicle or 50 mg/kg clemastine for 12 days starting at day 15 after tumour implantation. No human glioblastoma cells, visualized with an anti-human vimentin (hVim, red), were observed. Nuclei were visualized by using DAPI (white). Scale bar: 1 mm.

C Quantification of the primary tumour volume of vehicle-treated (black bars) and clemastine-treated (white bars) animals. BT12 parental (wt, Ve $n$ = 9, Cle $n$ = 12), control shRNA infected BT12 (Scr, Ve $n$ = 5, Cle $n$ = 5), MDGI silenced BT12 (sh1 Ve $n$ = 5, Cle $n$ = 5), BT13 (Ve $n$ = 10, Cle $n$ = 9) and ZH305 (Ve $n$ = 10, Cle $n$ = 10). Since no primary tumour mass was observed in brains of animals injected with the ZH305 cells, the area occupied by the tumour cells was quantified. Data are represented as mean ± SD. ****$P < 0.0001$, two-tailed, nonparametric Mann–Whitney's $U$-test.

D, E Representative micrographs of the vehicle- and clemastine-treated xenografts stained with antibodies against human nuclear membrane antigen (hNUMA, red) to visualize glioma cells. Galectin-1 (LGALS1, green) showed diffusive staining in the vehicle-treated tumours (D), while it was seen in more punctate pattern in clemastine-treated tumours (E). Scale bar 10 μm.

F Western blot analyses of galectin-1 (LGALS1) expression in glioblastoma cells in murine brain treated with vehicle ($n$ = 4) or clemastine ($n$ = 4). Since the whole-brain extracts were analysed, anti-human vimentin (hVim) was used to evaluate the amount of tumour cells within the brain. Numbers indicate the relative expression of LGALS1 compared to the levels of hVim that was set as 1 in each xenograft. No significant difference was observed between the vehicle- and clemastine-treated tumours.

Source data are available online for this figure.


© 2019 The Authors

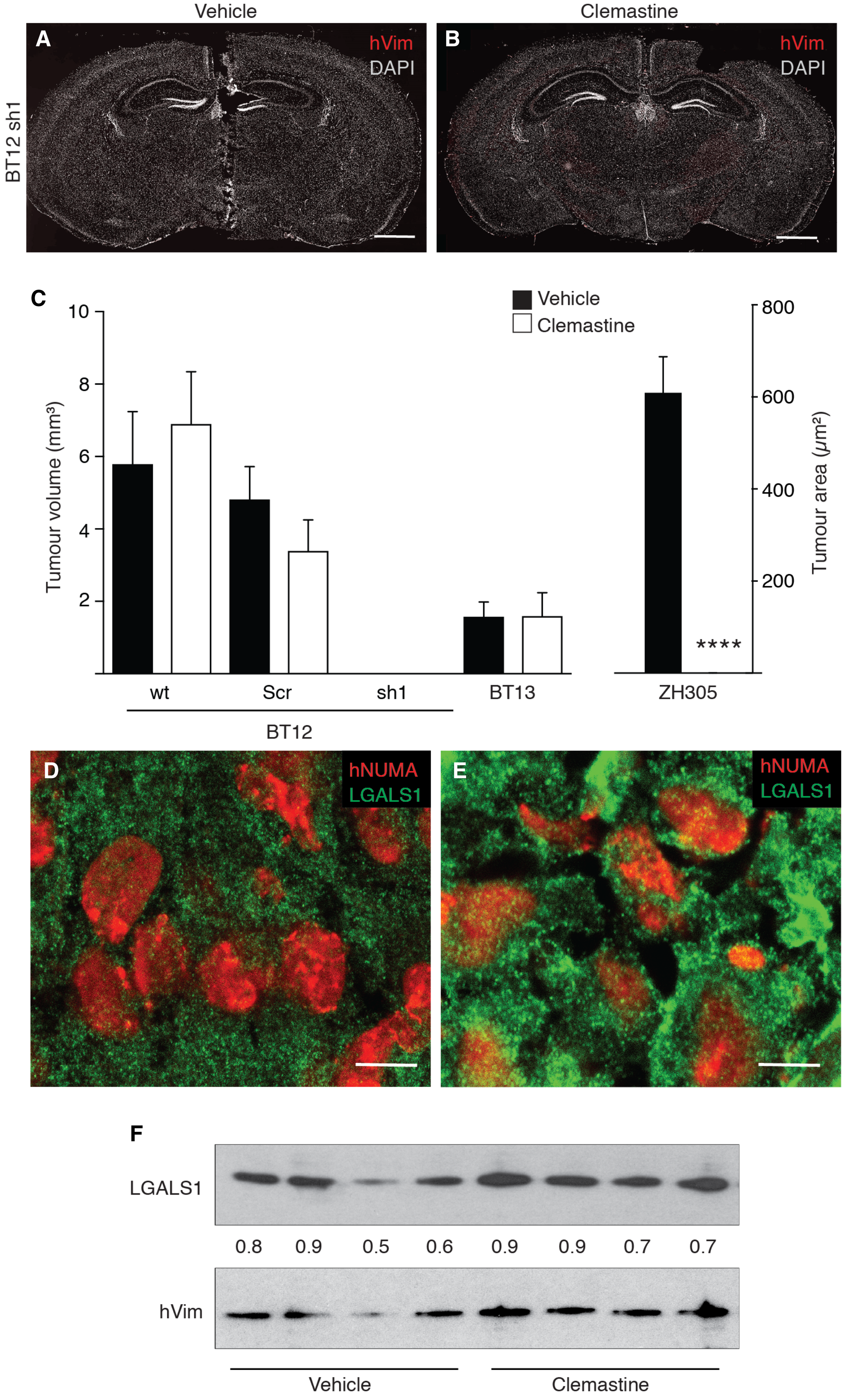
Vehicle
Clemastine
A
hVim
DAPI
B
hVim
DAPI
BT12 sh1
C
Vehicle
Clemastine
Tumour volume (mm³)
Tumour area (μm²)
10
8
6
4
2
800
600
400
200
****
wt
Scr
sh1
BT13
ZH305
BT12
D
hNUMA
LGALS1
E
hNUMA
LGALS1
F
LGALS1
0.8 0.9 0.5 0.6 0.9 0.9 0.7 0.7
hVim
Vehicle
Clemastine

**Figure EV4.**
