# Supplementary material for: Vulnerability of invasive glioblastoma cells to lysosomal membrane destabilization
Source: EMBO Mol Med. 2019 May 8;11(6):e9034. doi: 10.15252/emmm.201809034 (PMC6554674; doi:10.15252/emmm.201809034)

Source data for Expanded view Fig EV3

A
BT12 shMDGI
LGALS1

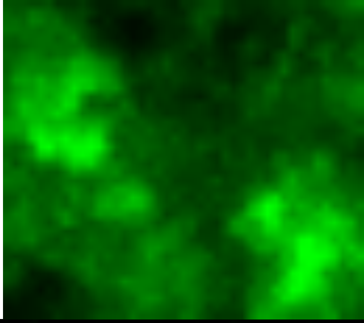

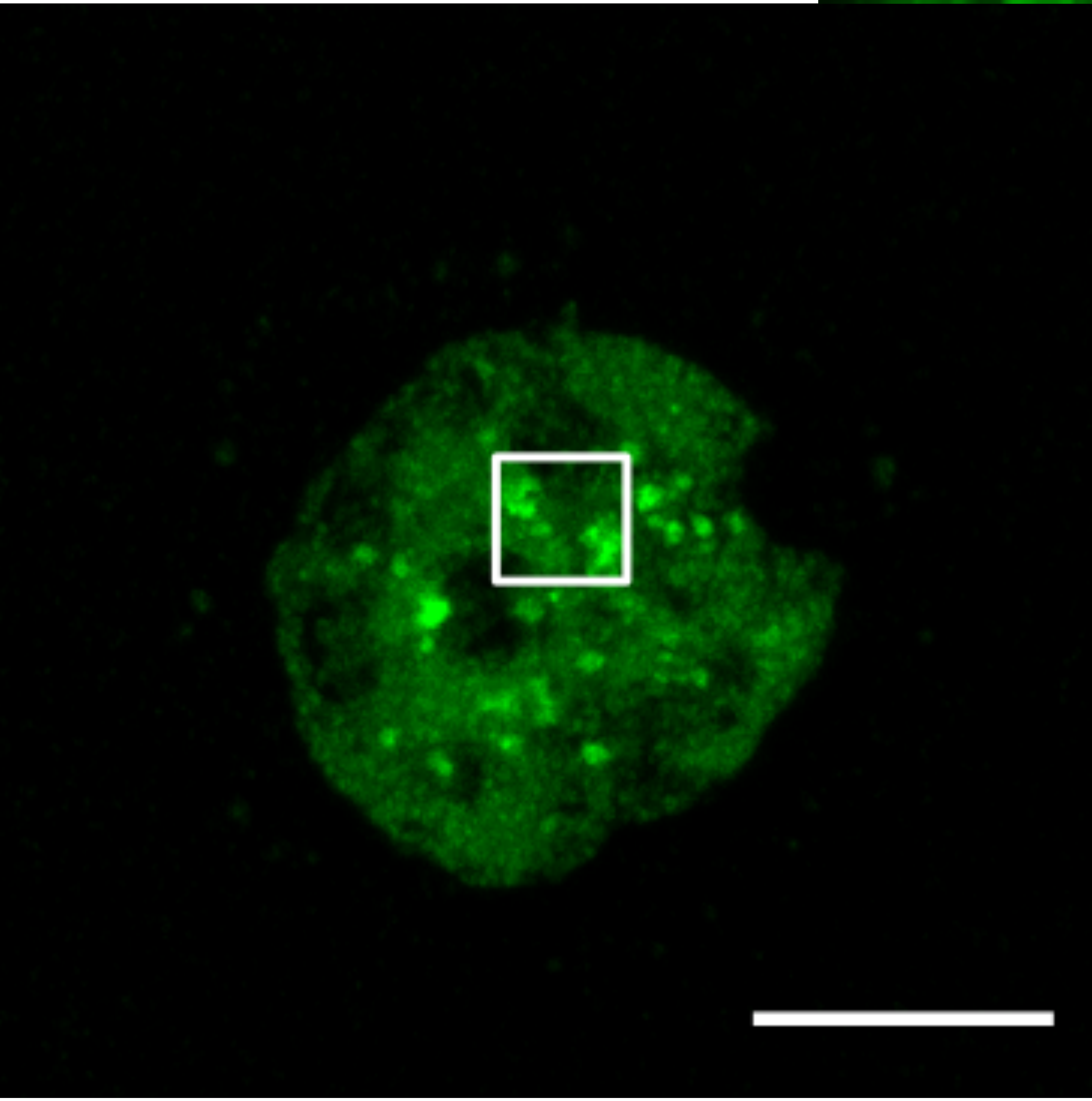

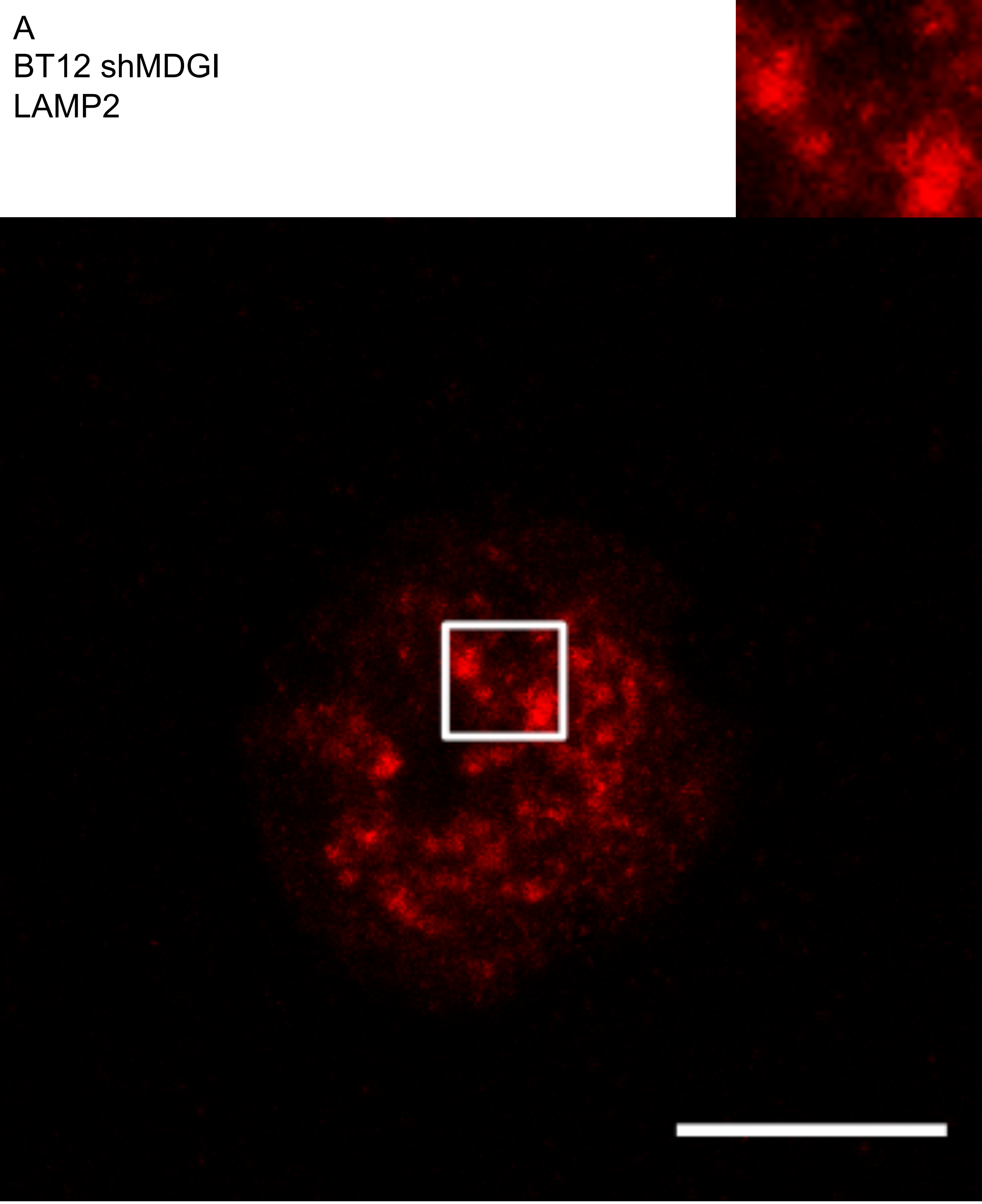
A
BT12 shMDGI
LAMP2

A
BT12 shMDGI
Merge

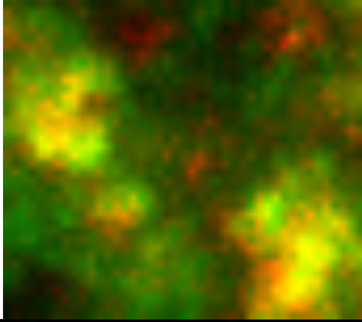

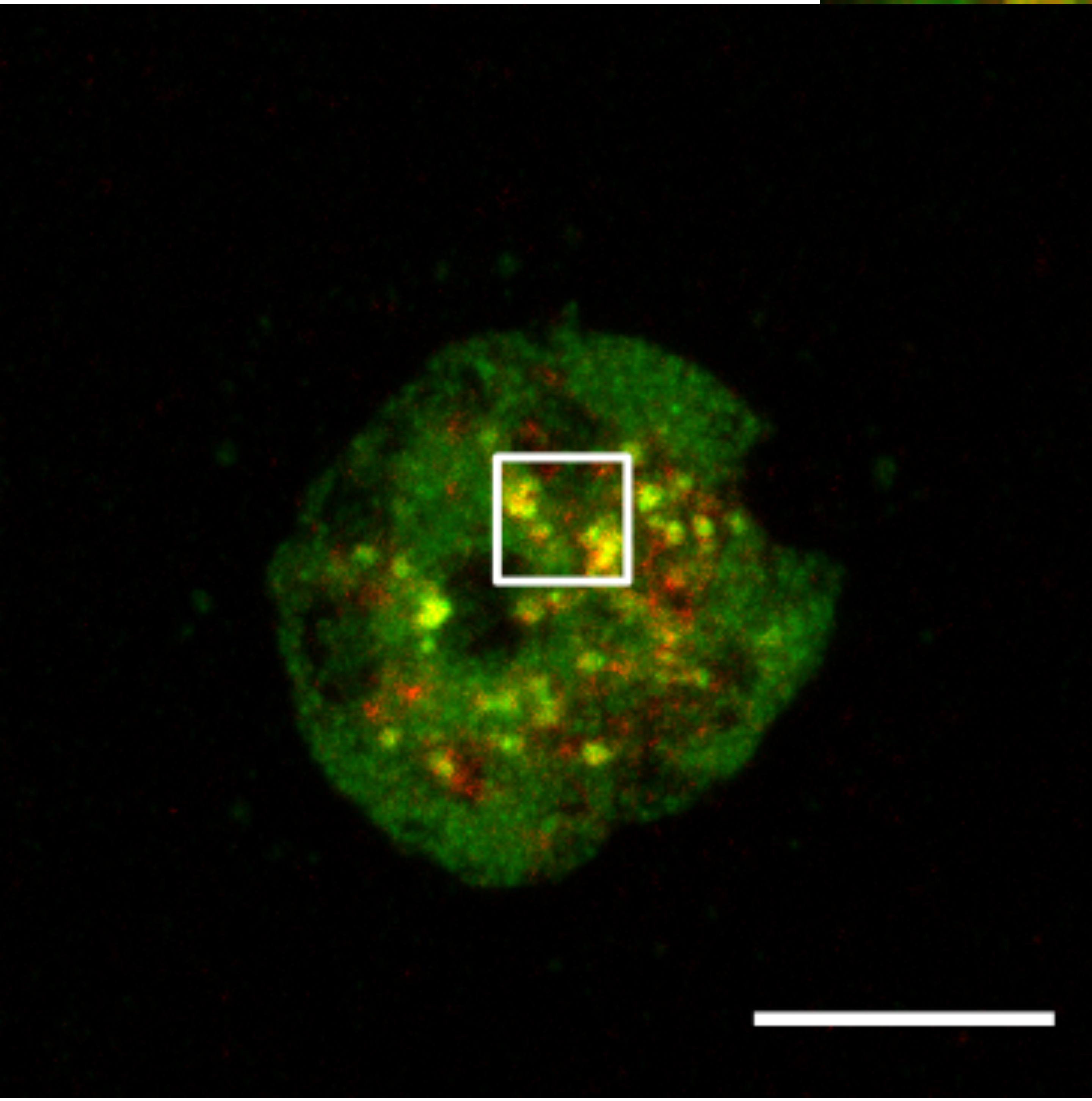

A
BT13 shMDGI
LGALS1

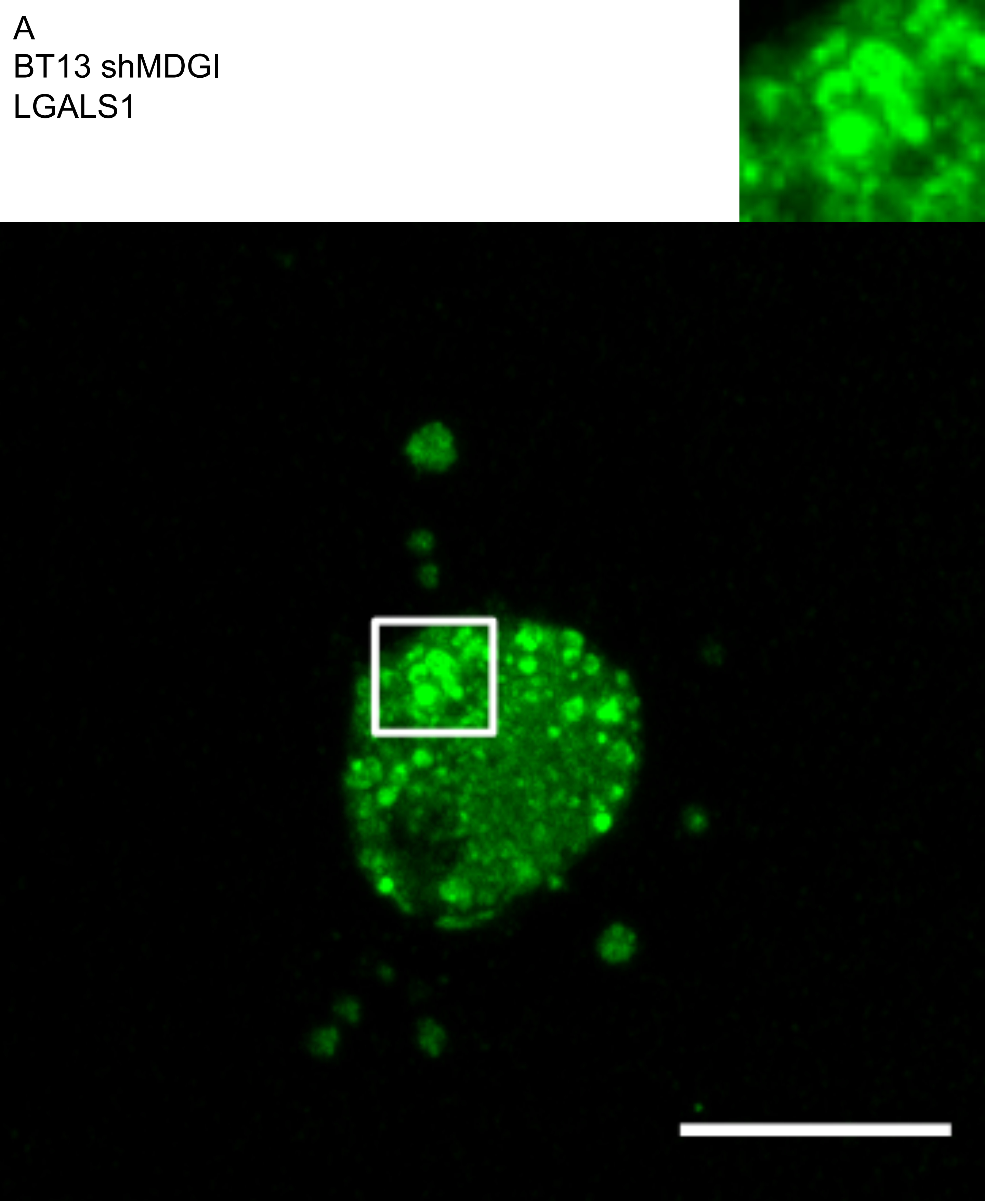

A
BT13 shMDGI
LAMP2

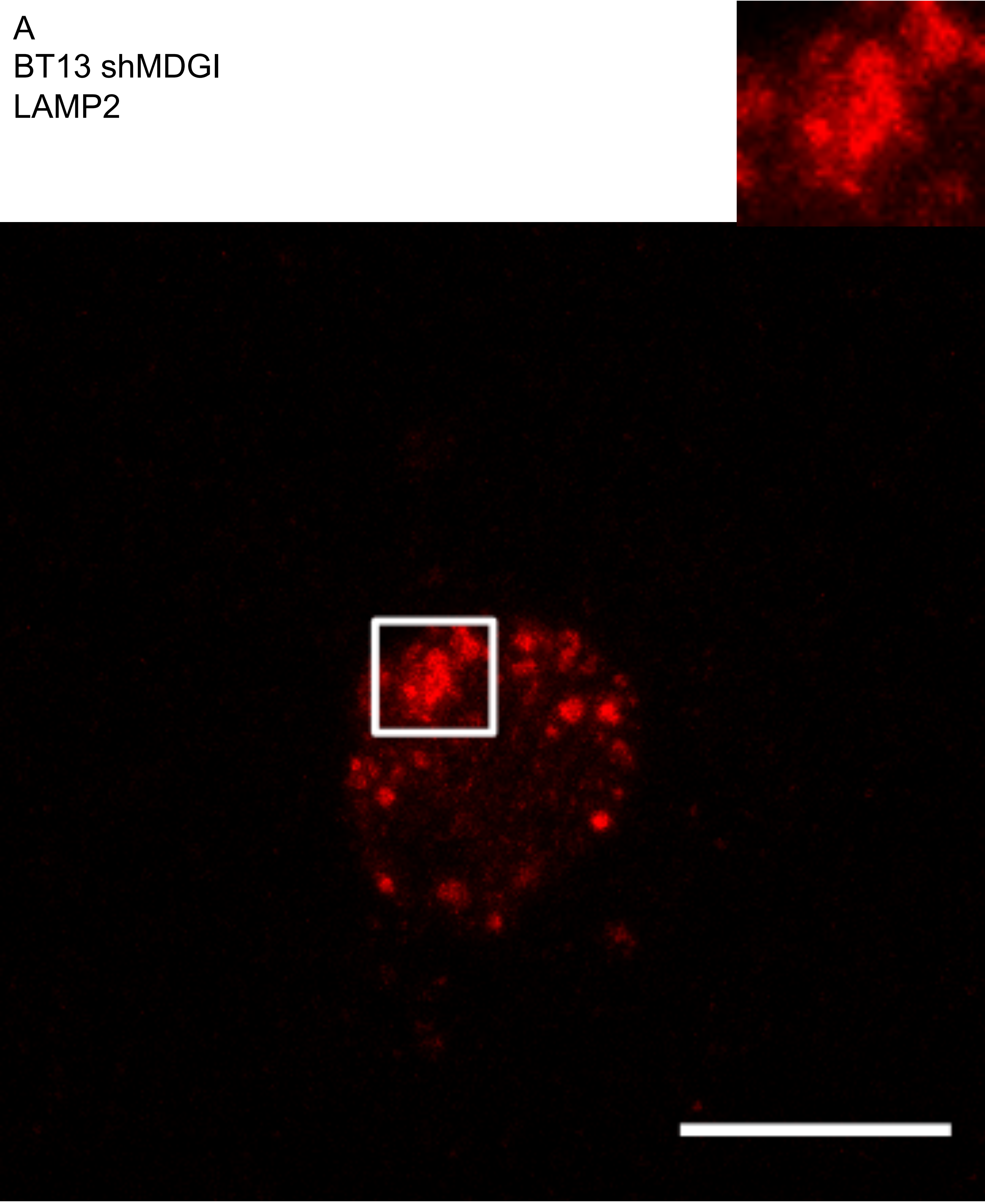

A
BT13 shMDGI
Merge

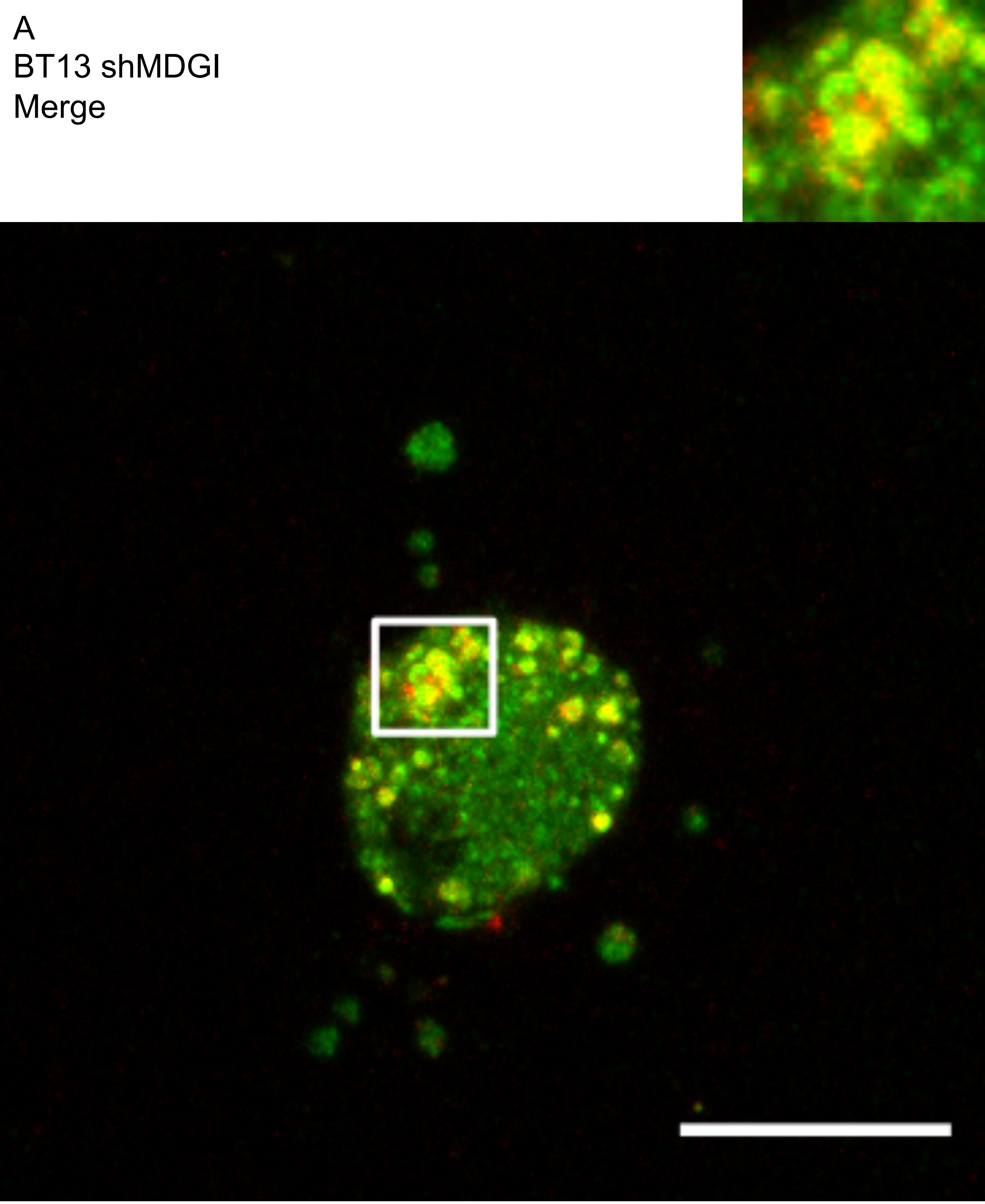

B

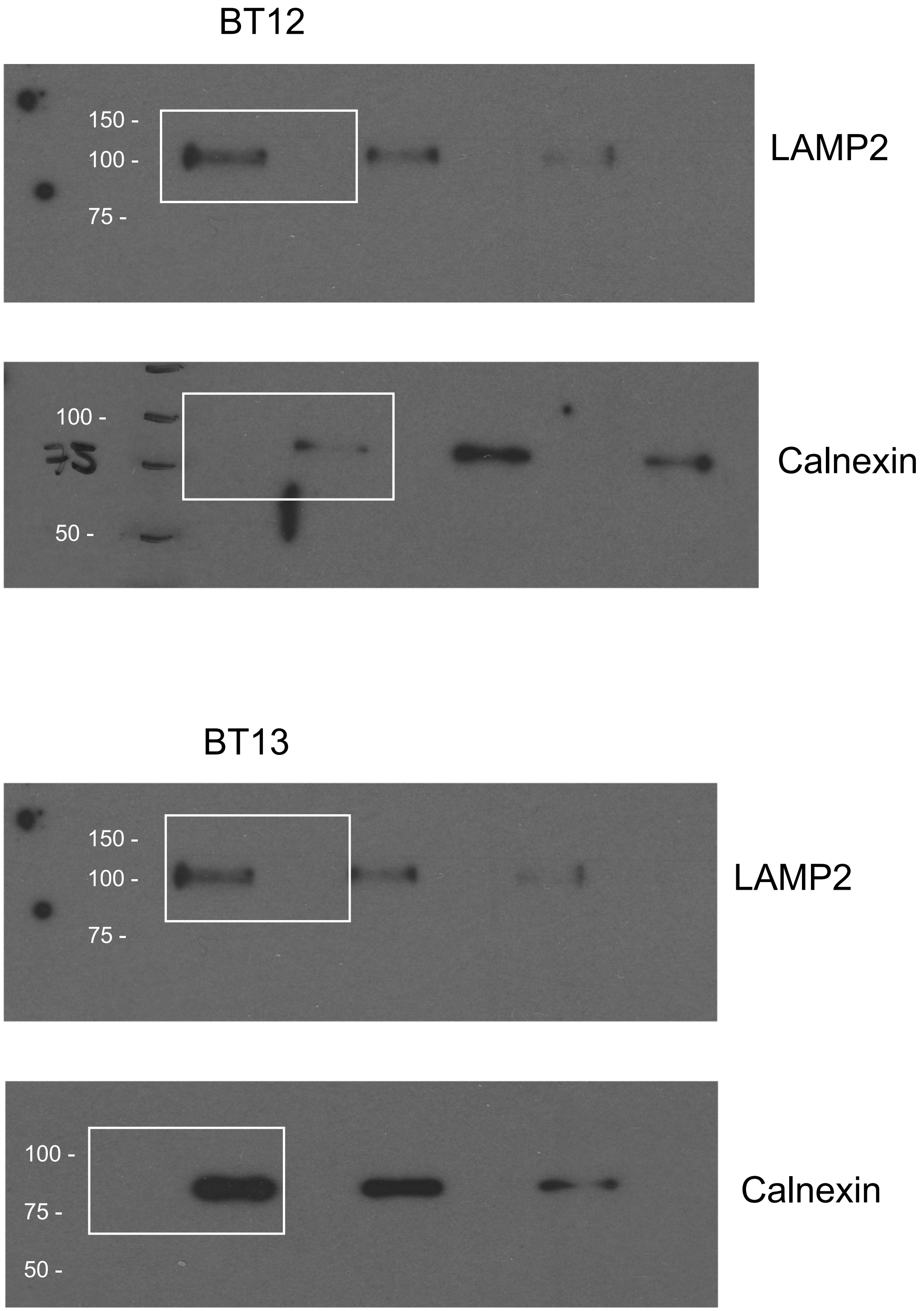
BT12
150 -
100 -
75 -
LAMP2
100 -
75
50 -
Calnexin
BT13
150 -
100 -
75 -
LAMP2
100 -
75 -
50 -
Calnexin

D

BT12 + clemastine

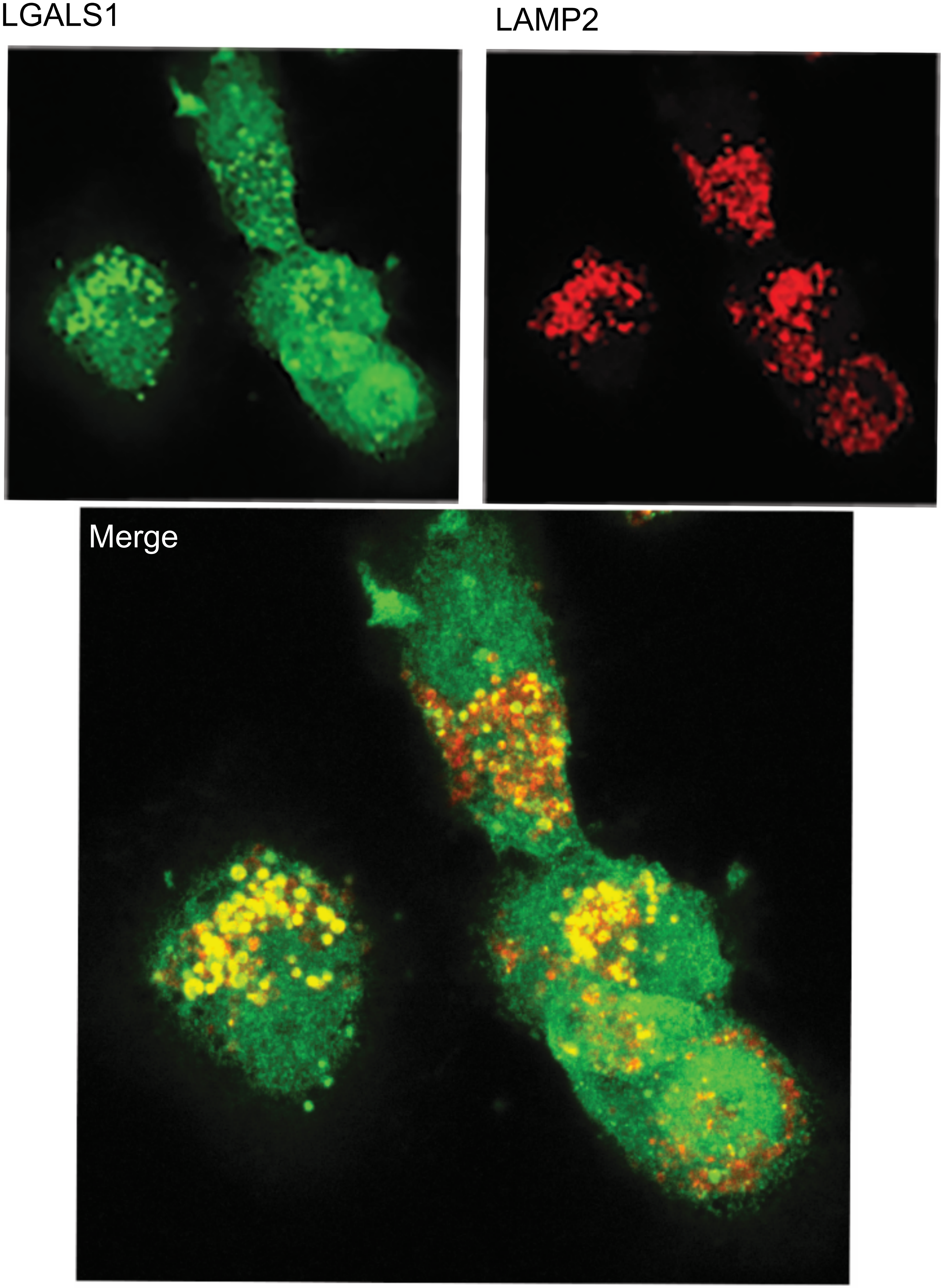

Supplement: Supplementary file 3 — Source Data for Expanded View and Appendix [file EMMM-11-e9034-s009.zip › 9034_EV_Appendix_SD/Figure_EV3_Source.pdf]
