# Supplementary material for: Vulnerability of invasive glioblastoma cells to lysosomal membrane destabilization
Source: EMBO Mol Med. 2019 May 8;11(6):e9034. doi: 10.15252/emmm.201809034 (PMC6554674; doi:10.15252/emmm.201809034)

Source data for Appendix figures

Appendix Fig S2D

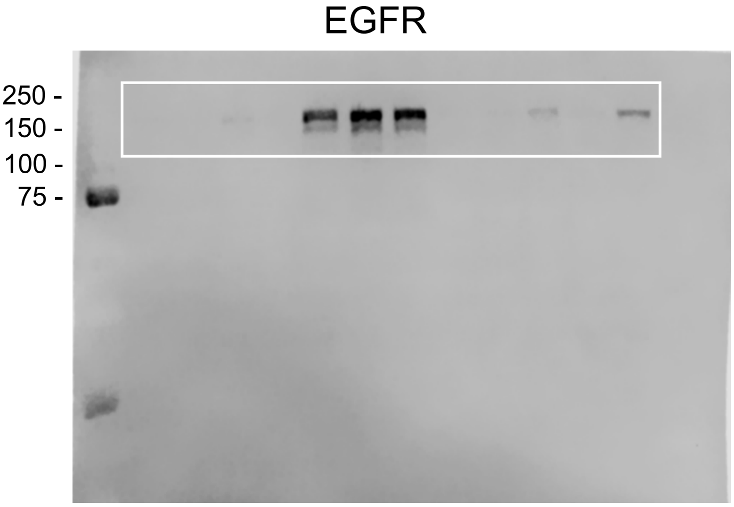

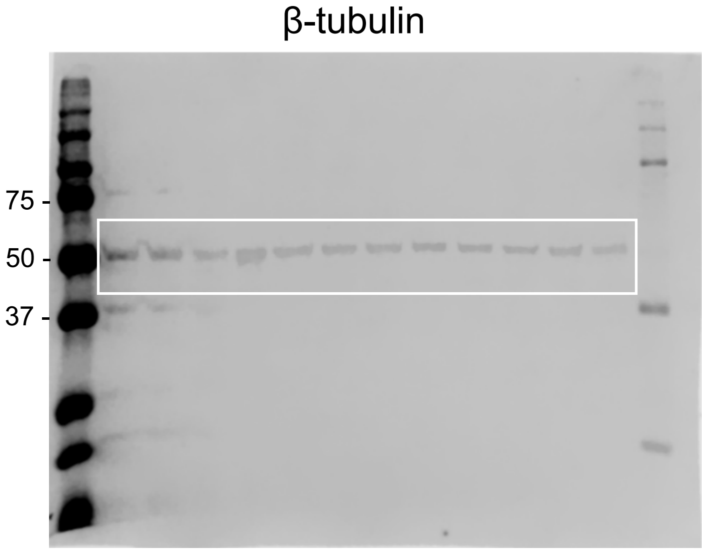

Appendix Fig S2G

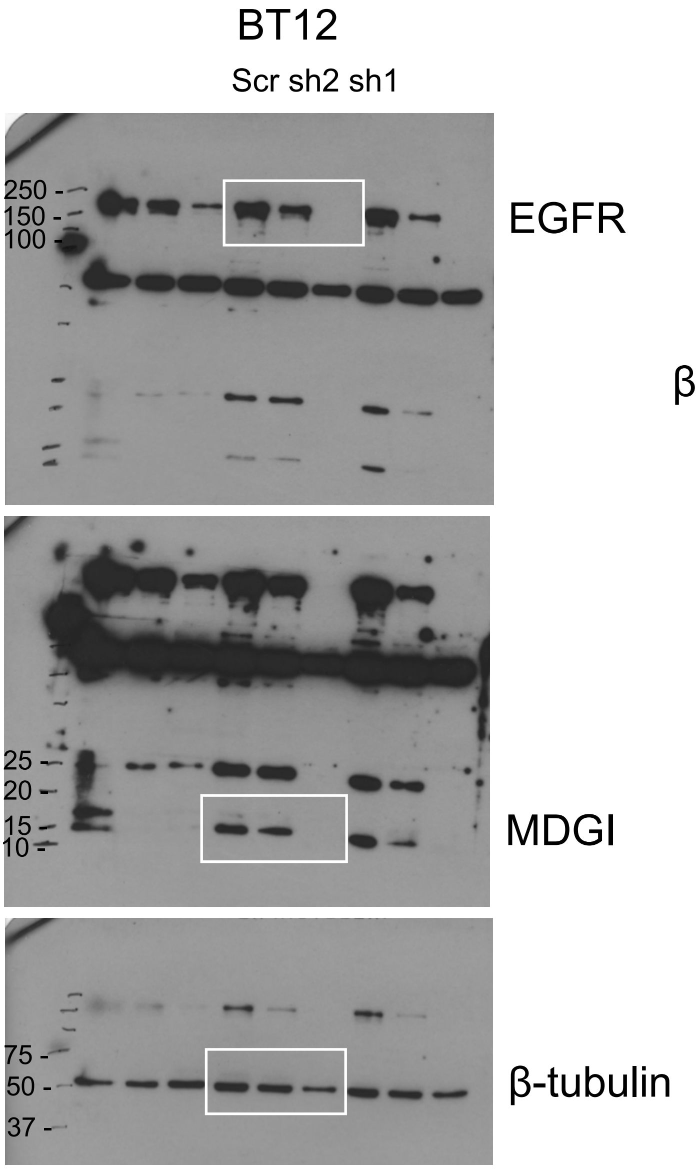

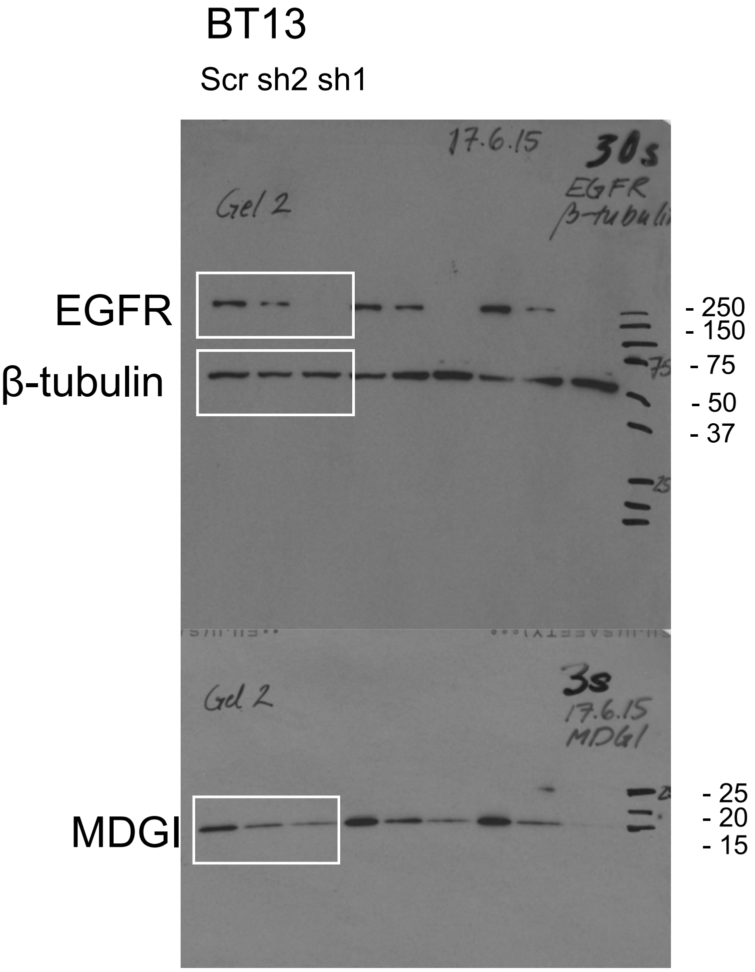

Appendix Fig S3A

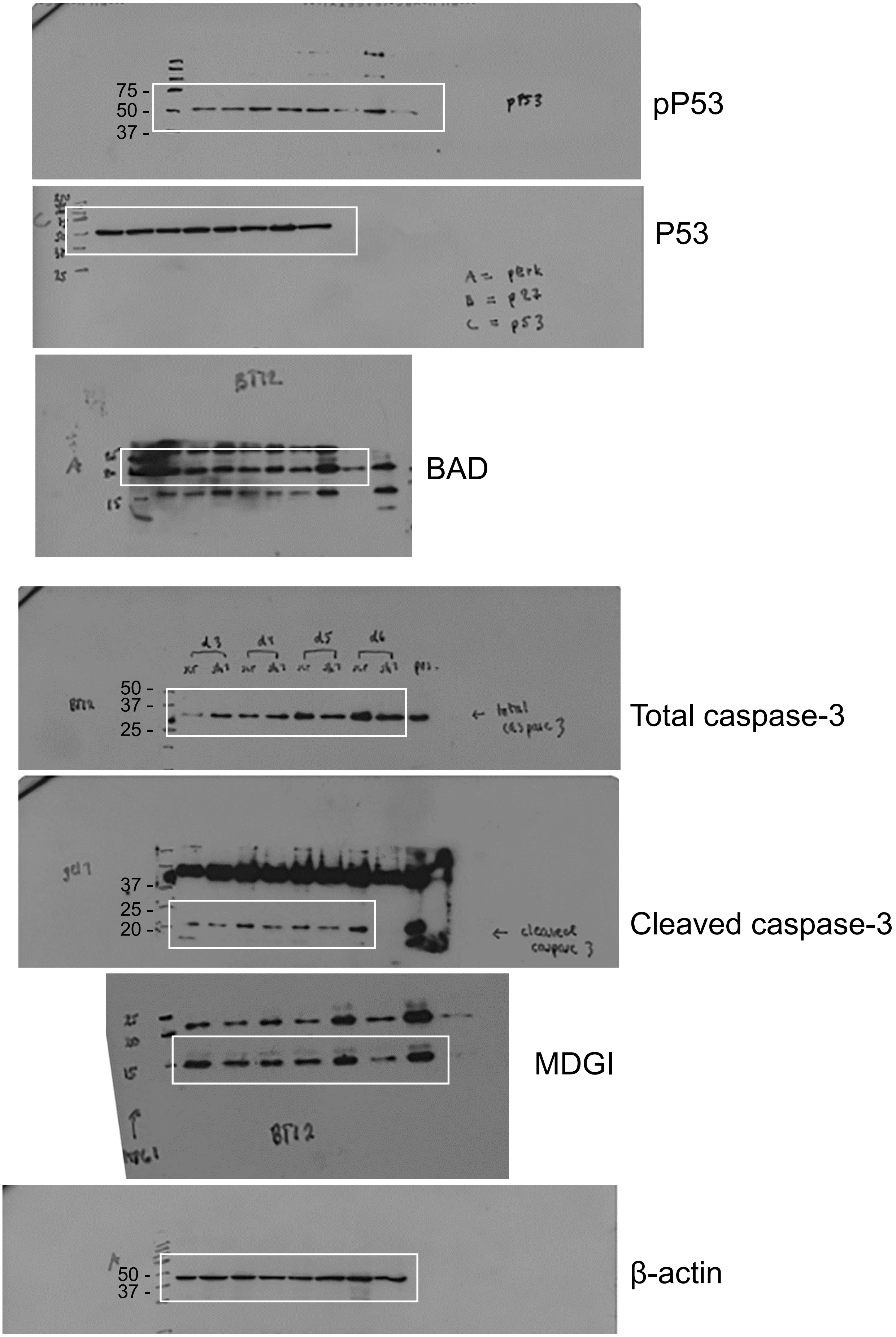

Appendix Fig S3B

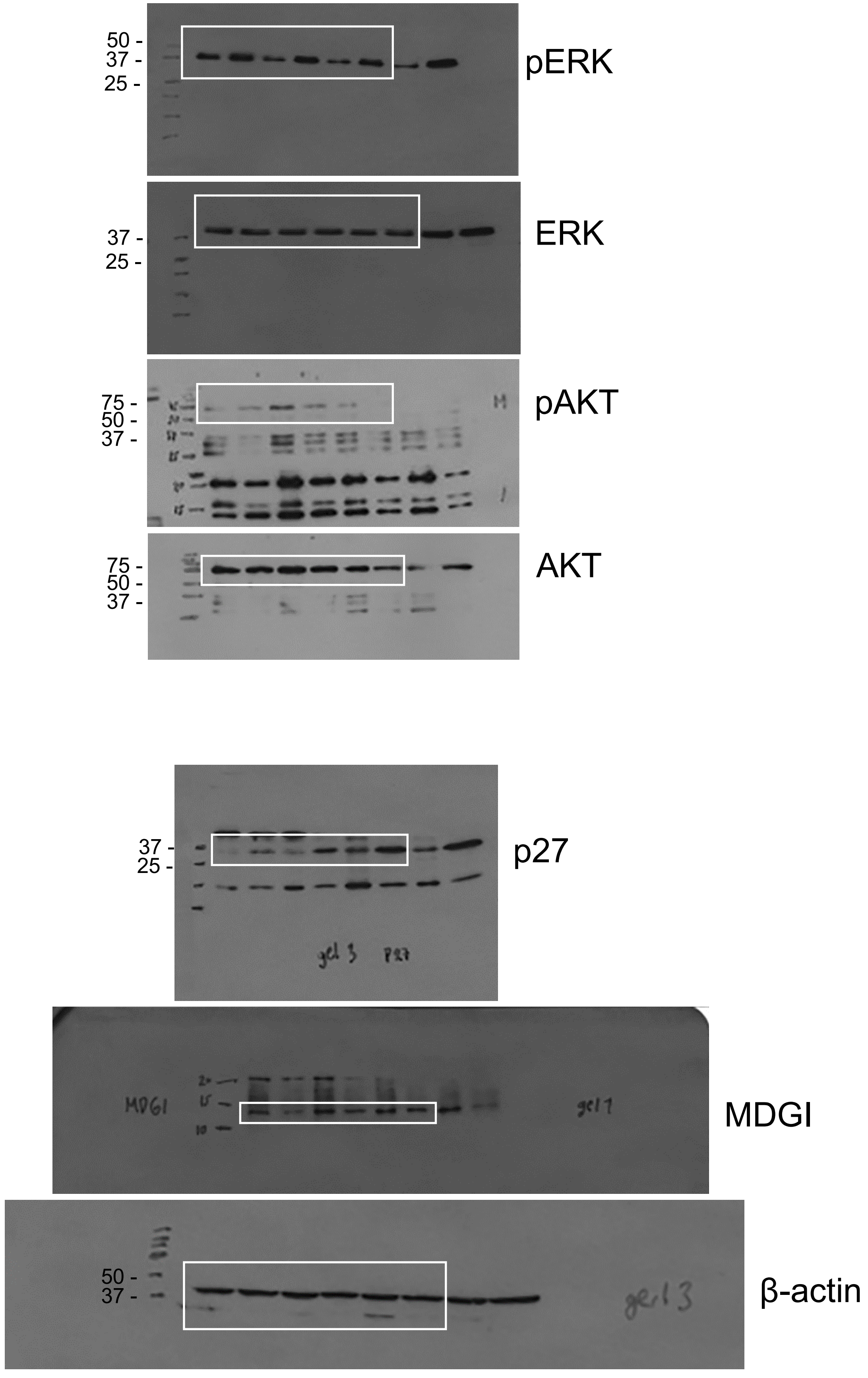

Appendix Fig S4A

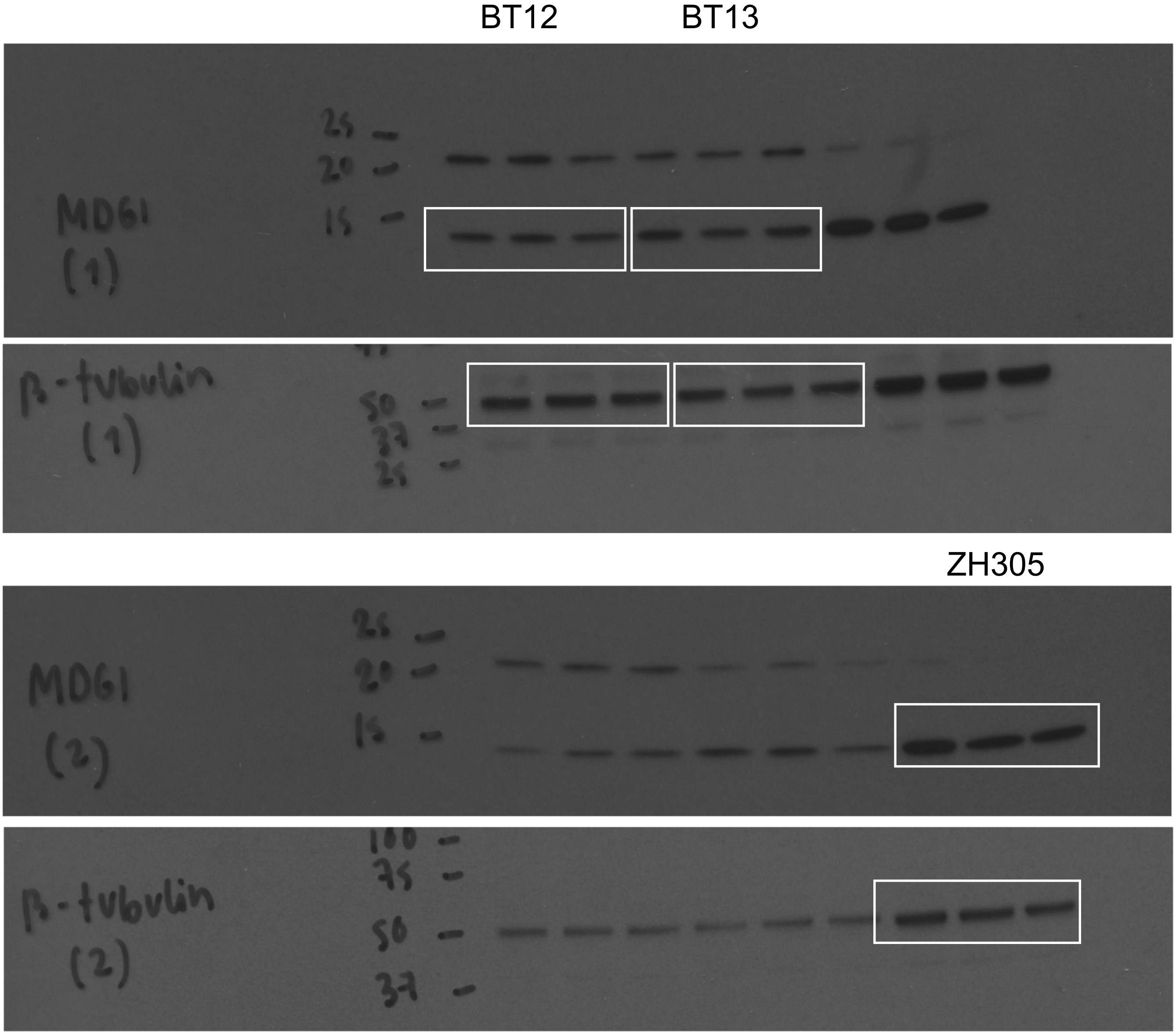

Supplement: Supplementary file 3 — Source Data for Expanded View and Appendix [file EMMM-11-e9034-s009.zip › 9034_EV_Appendix_SD/Appendix_Source_Data.pdf]
