# Supplementary figures and images for: Vulnerability of invasive glioblastoma cells to lysosomal membrane destabilization
Source: EMBO Mol Med. 2019 May 8;11(6):e9034. doi: 10.15252/emmm.201809034 (PMC6554674; doi:10.15252/emmm.201809034)

A

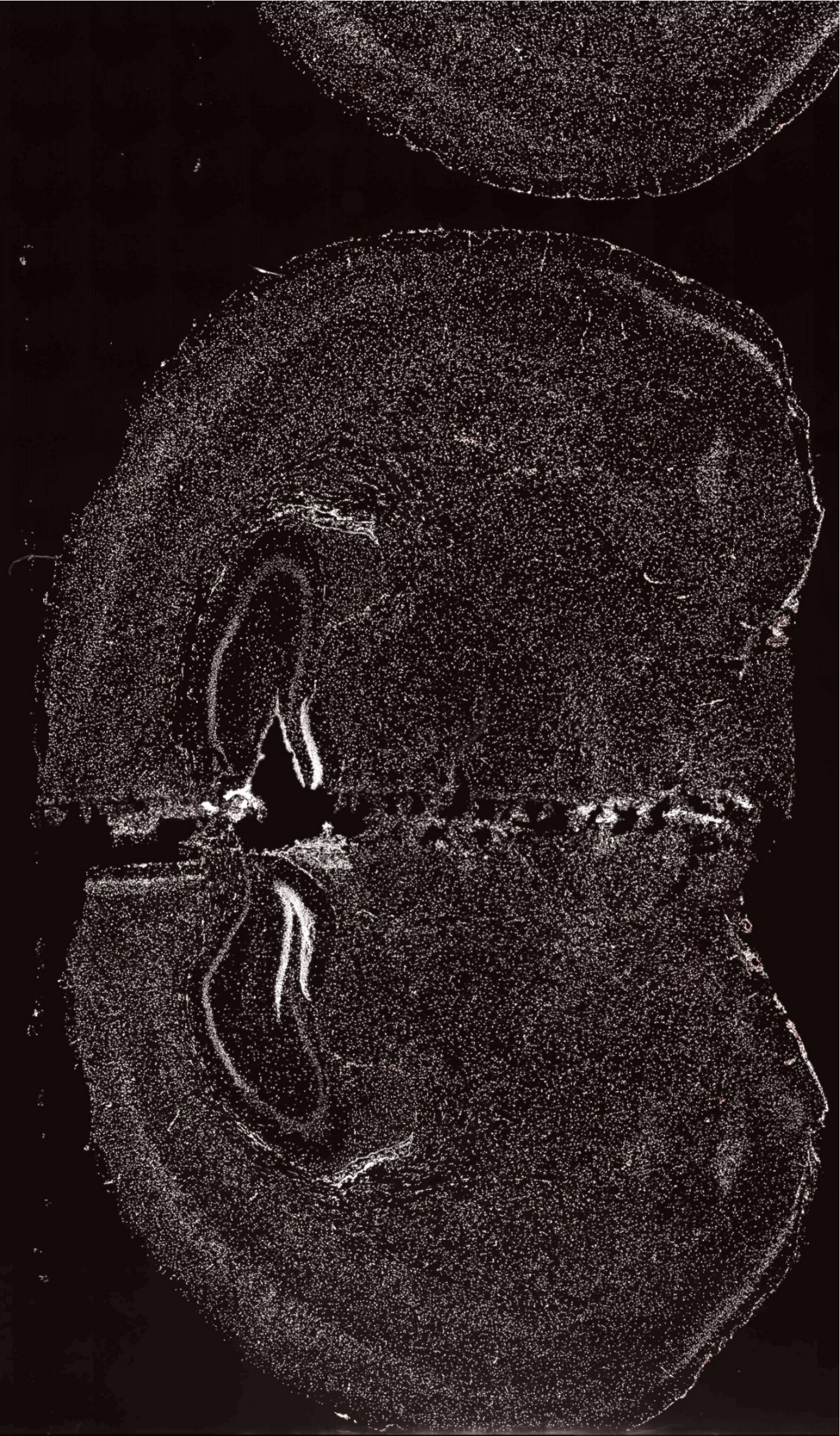

B

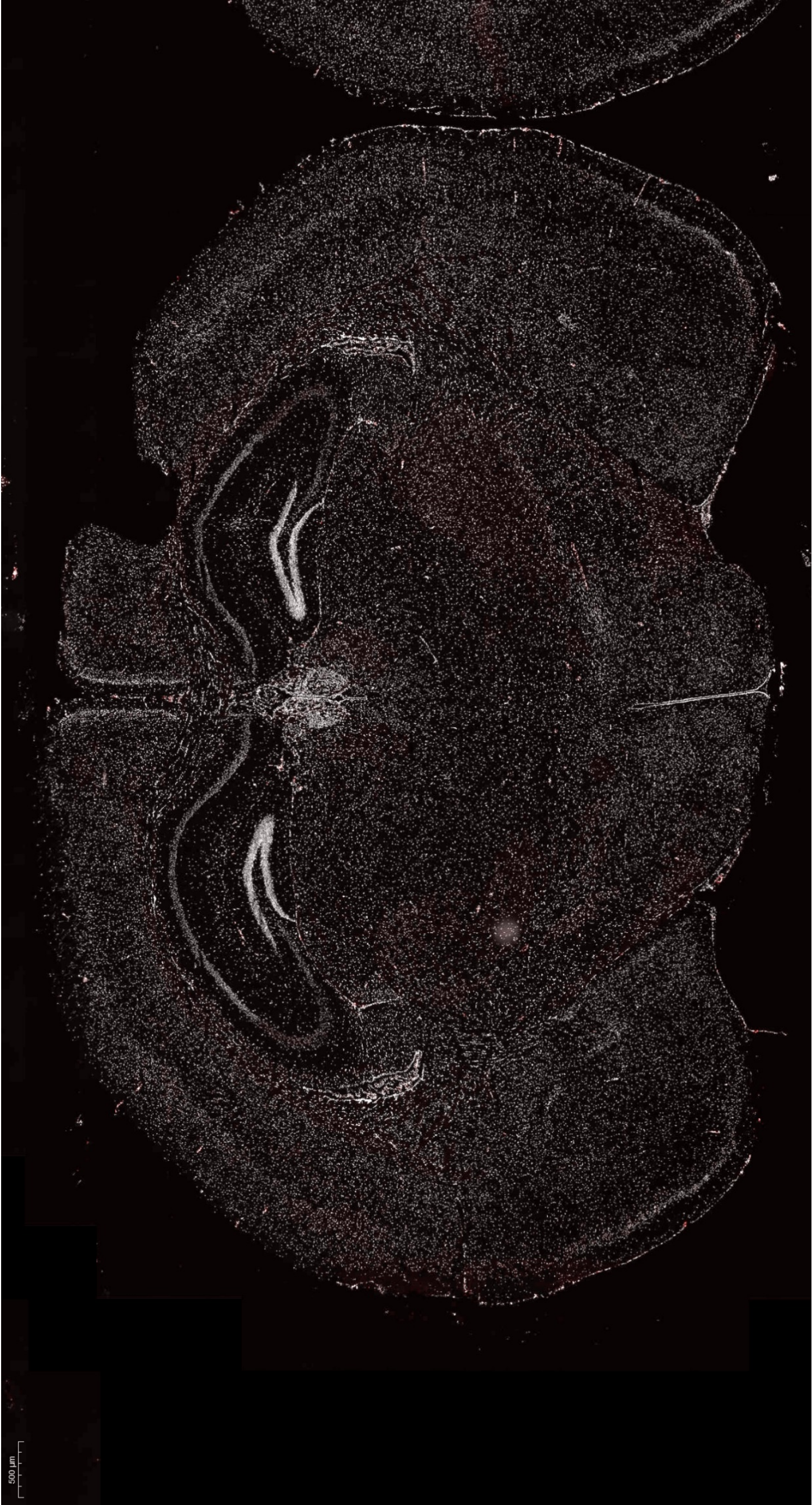

D

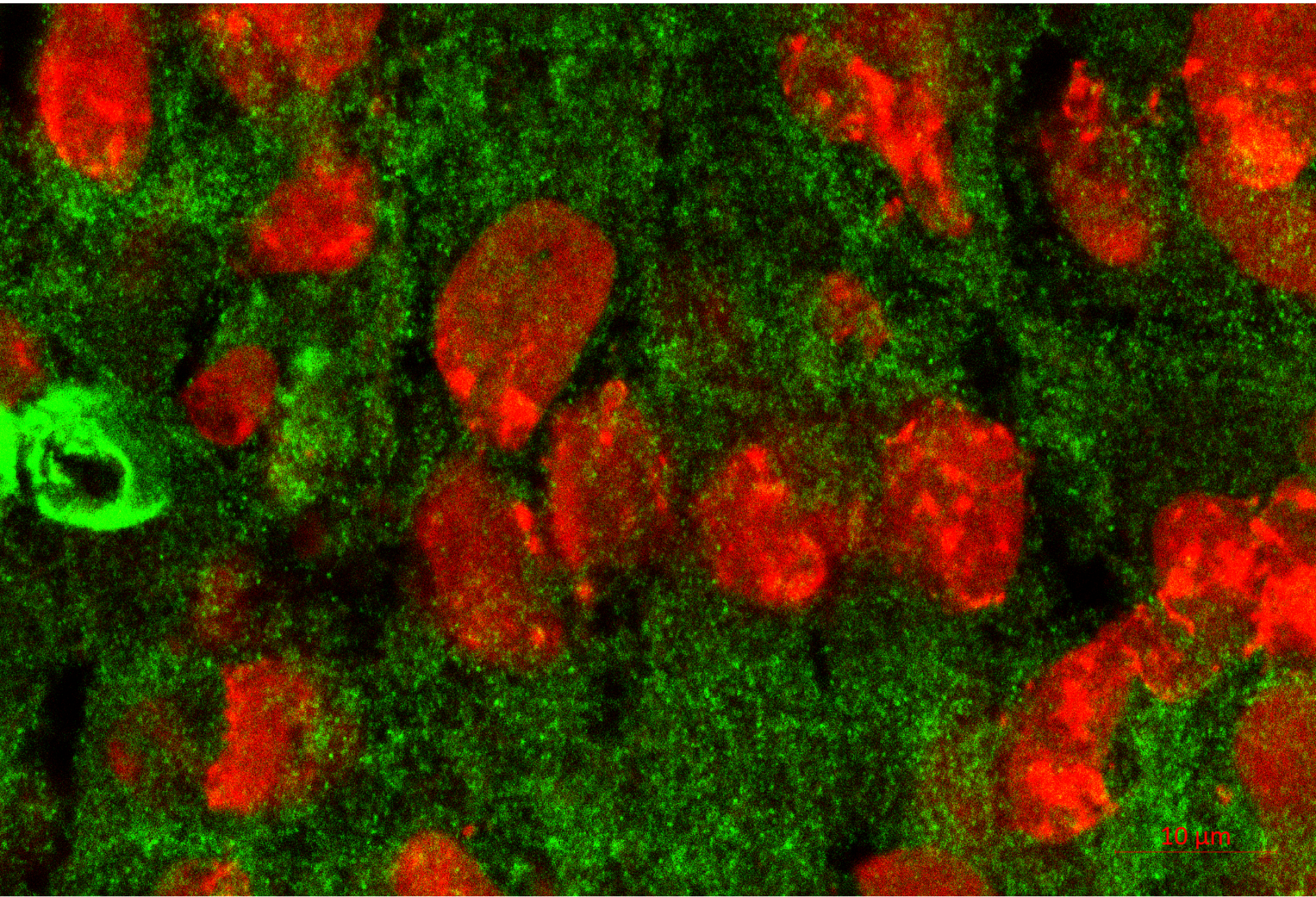

E

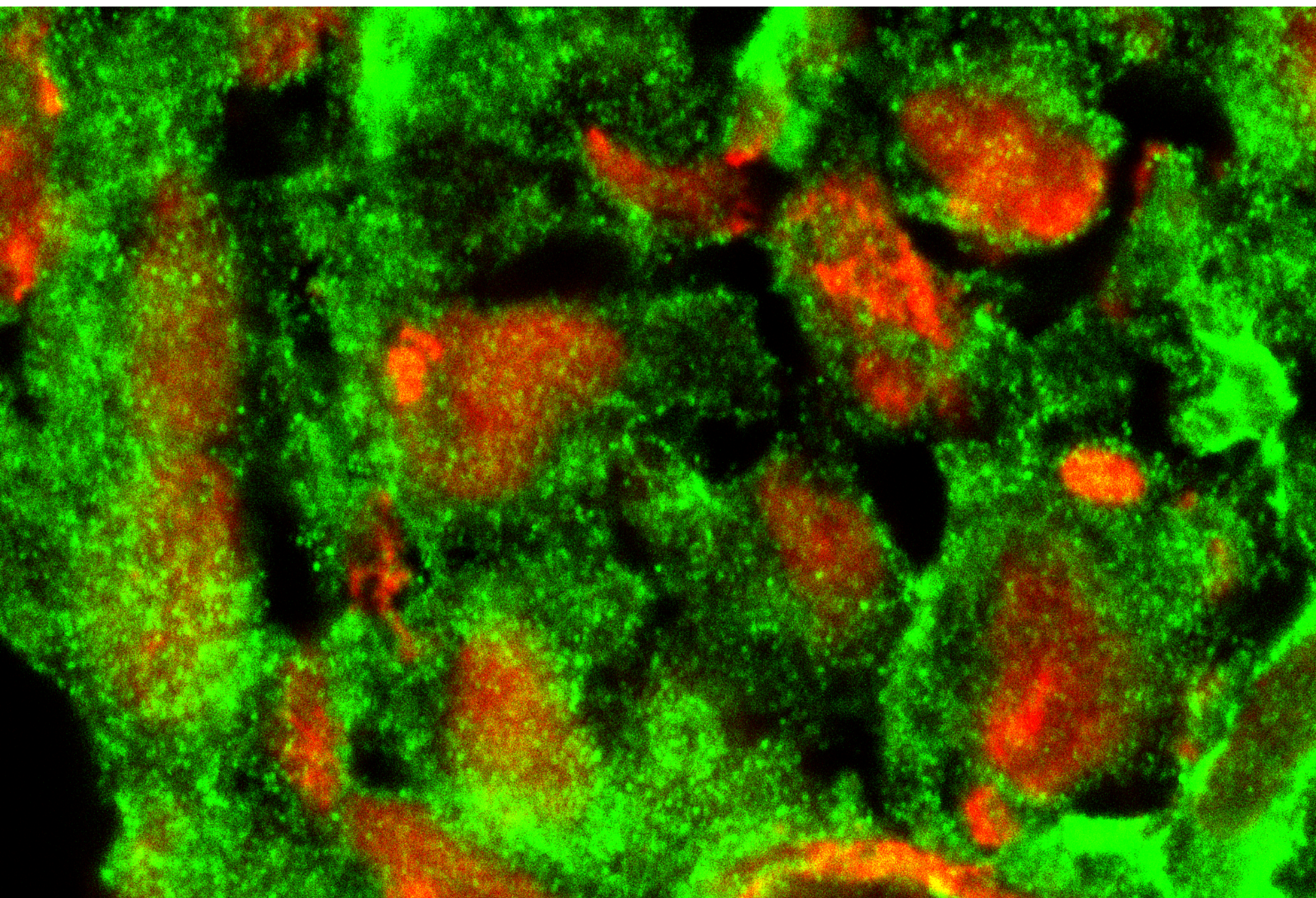

F

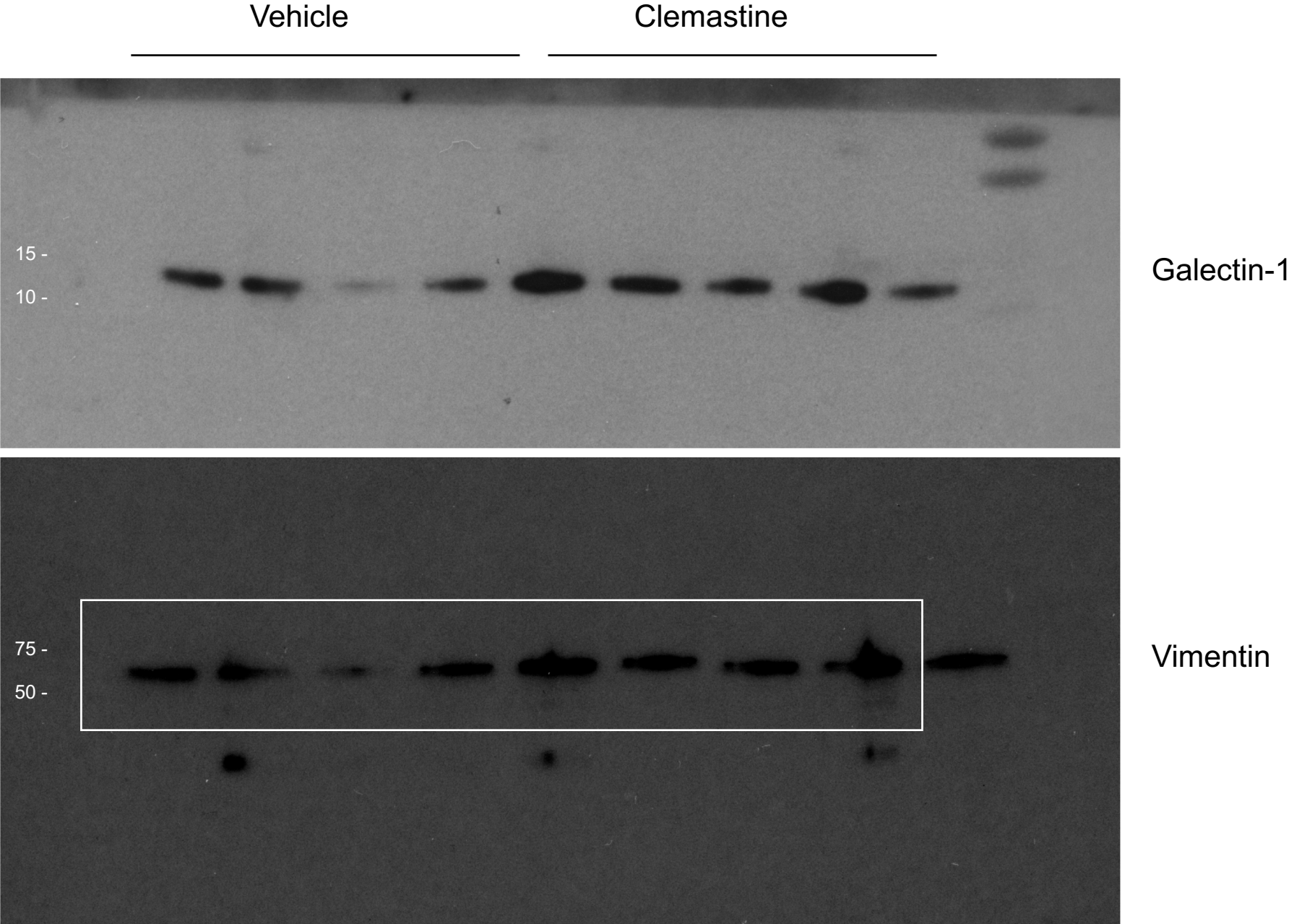

Supplement: Supplementary file 3 — Source Data for Expanded View and Appendix [file EMMM-11-e9034-s009.zip › 9034_EV_Appendix_SD/Figure_EV4_Source.pdf]

B  
GFP

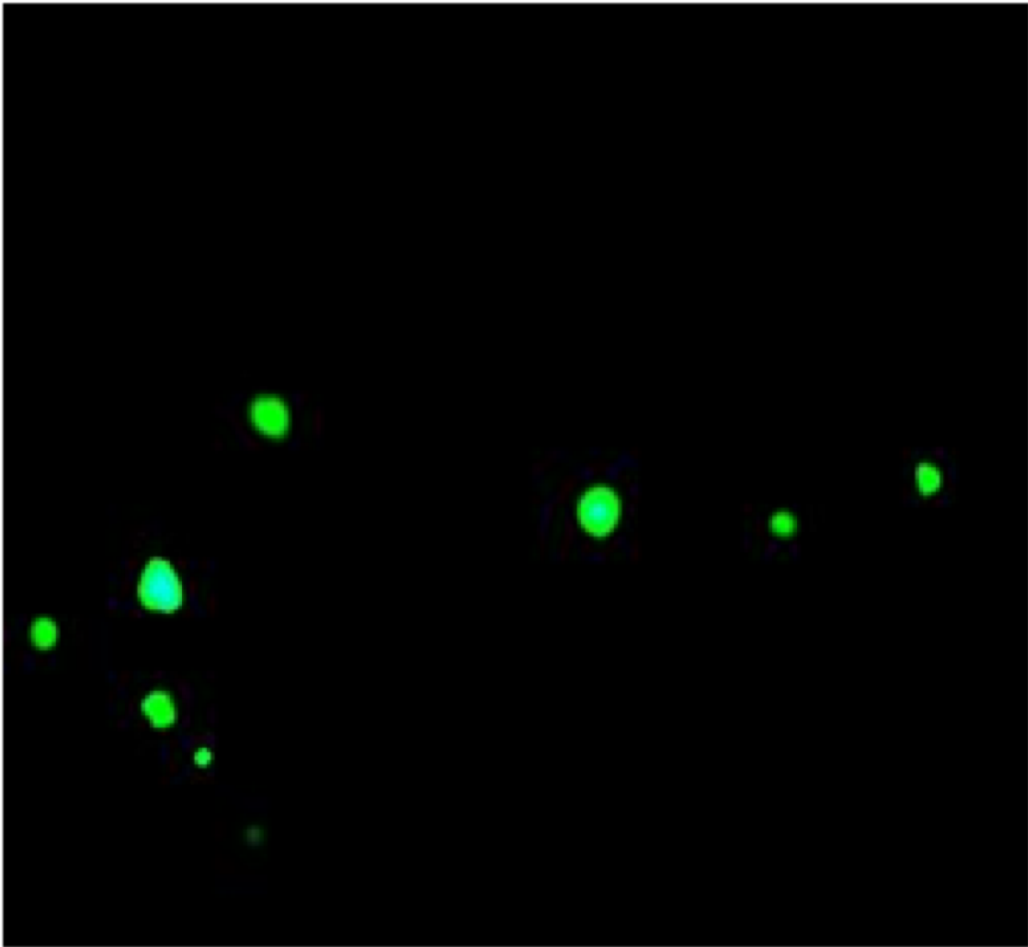

GFP-MDGI

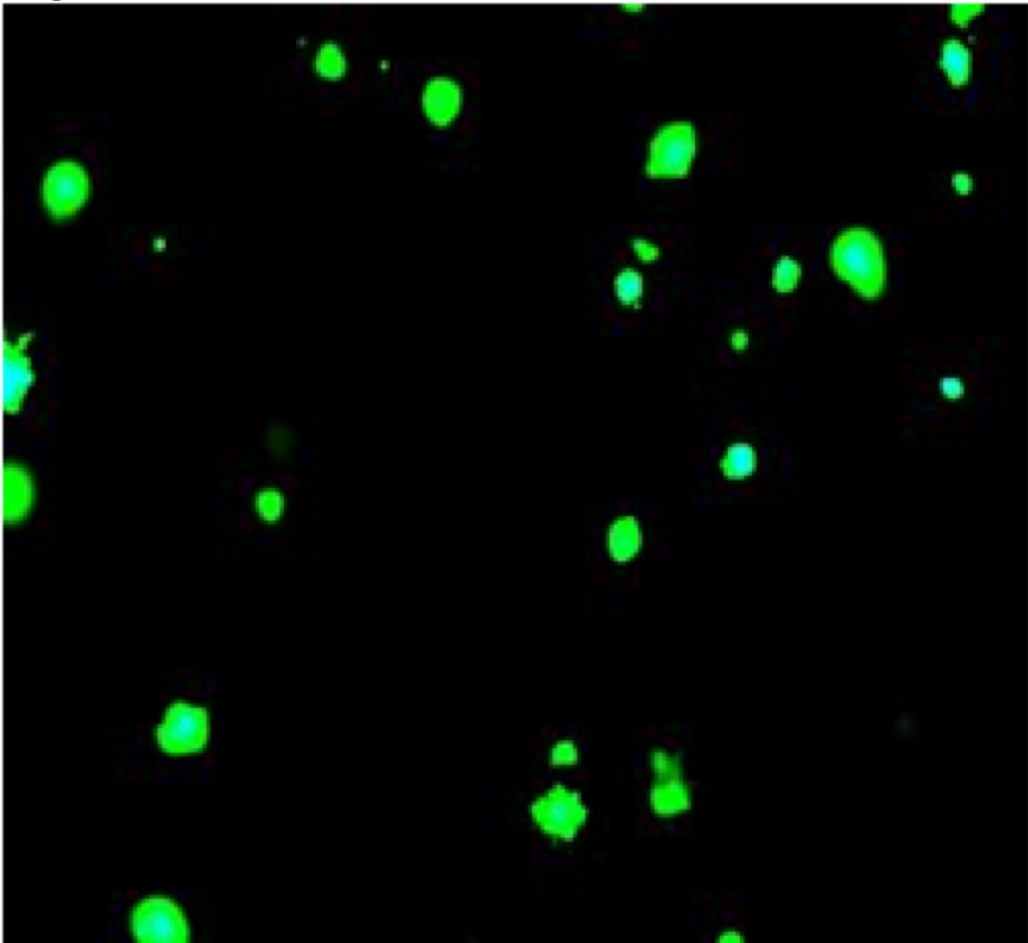

D  
GFP d0

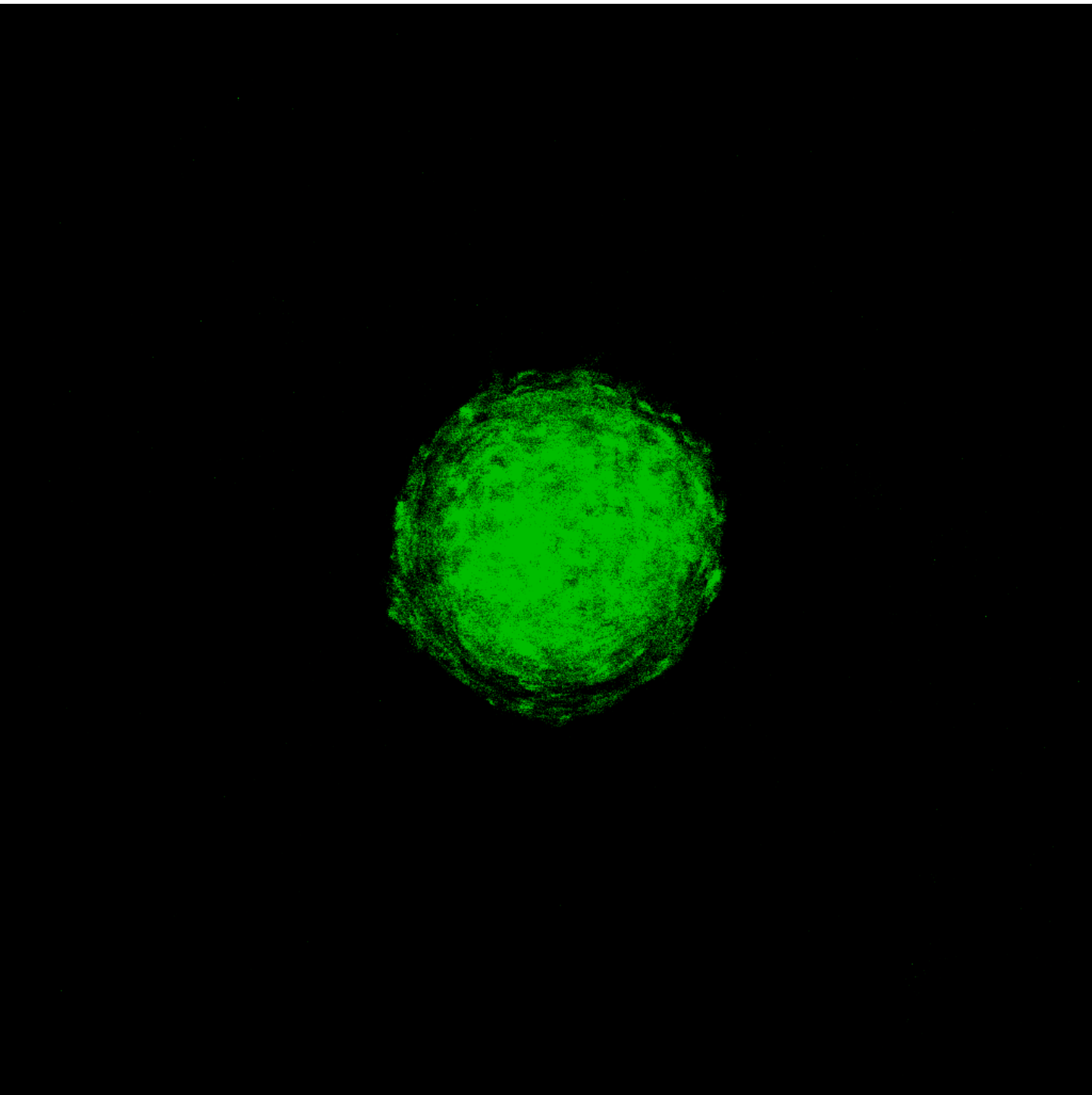

D  
GFP d2

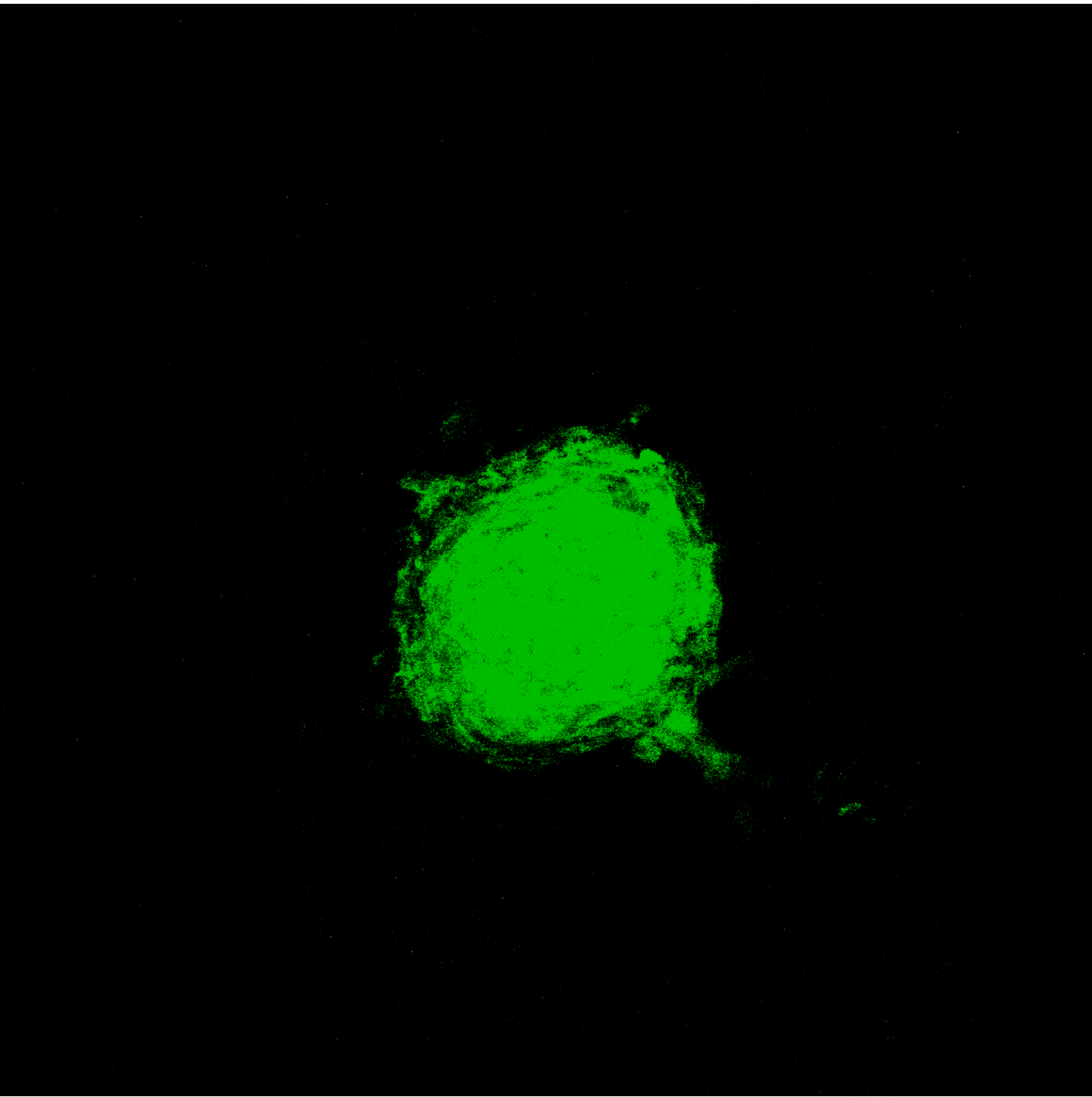

D  
GFP d5

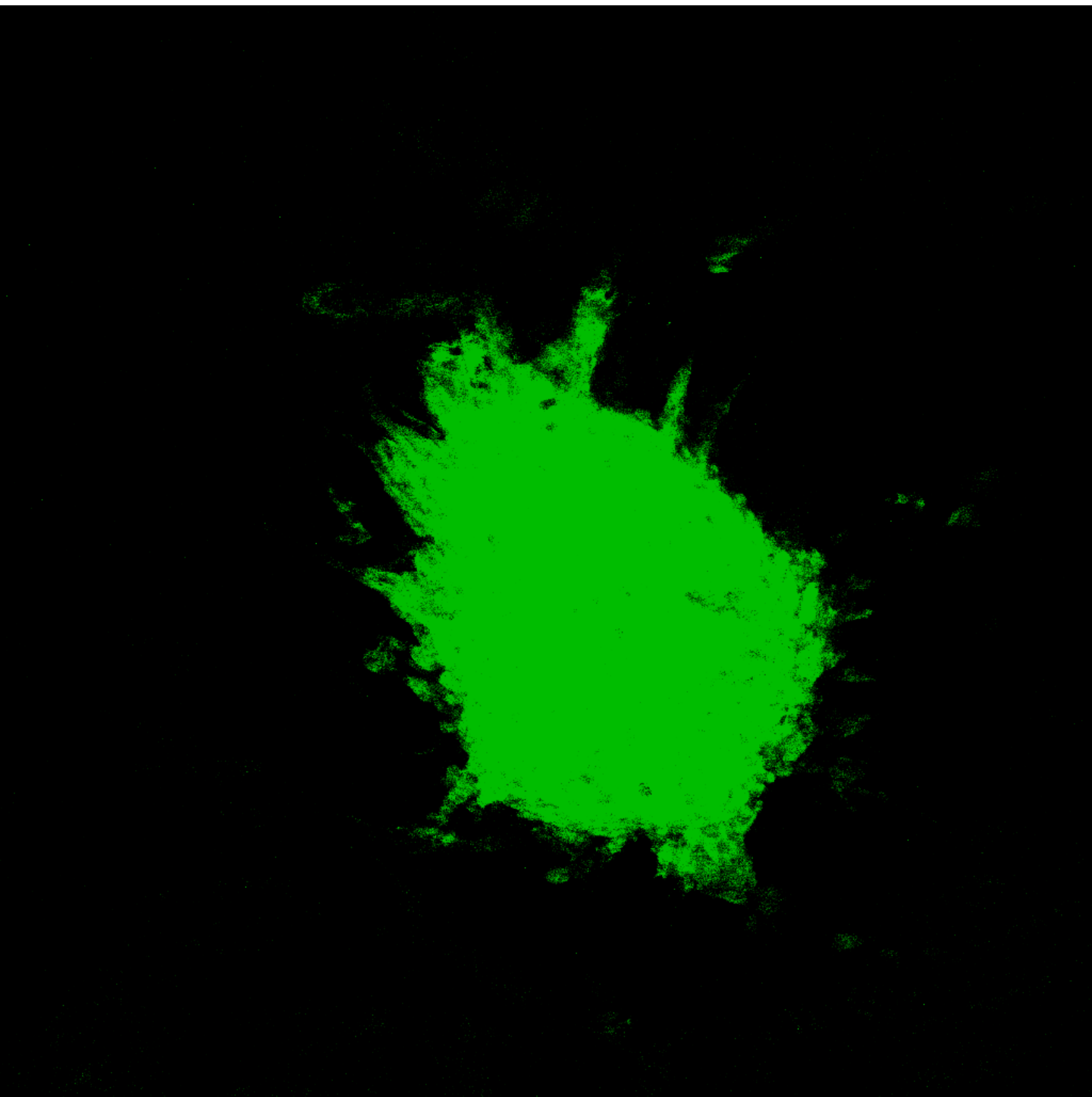

D  
GFP-MDGI d0

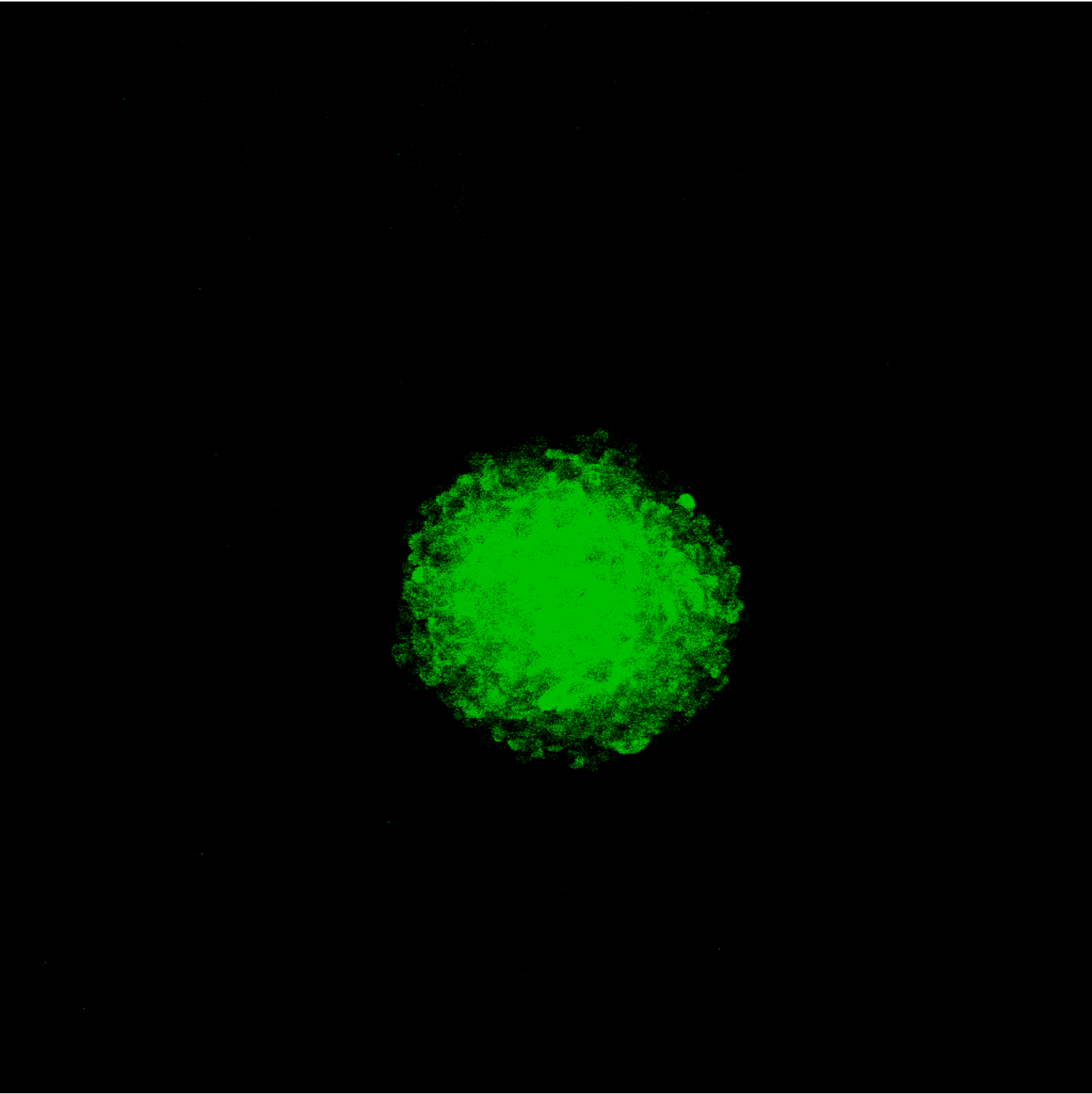

D  
GFP-MDGI d2

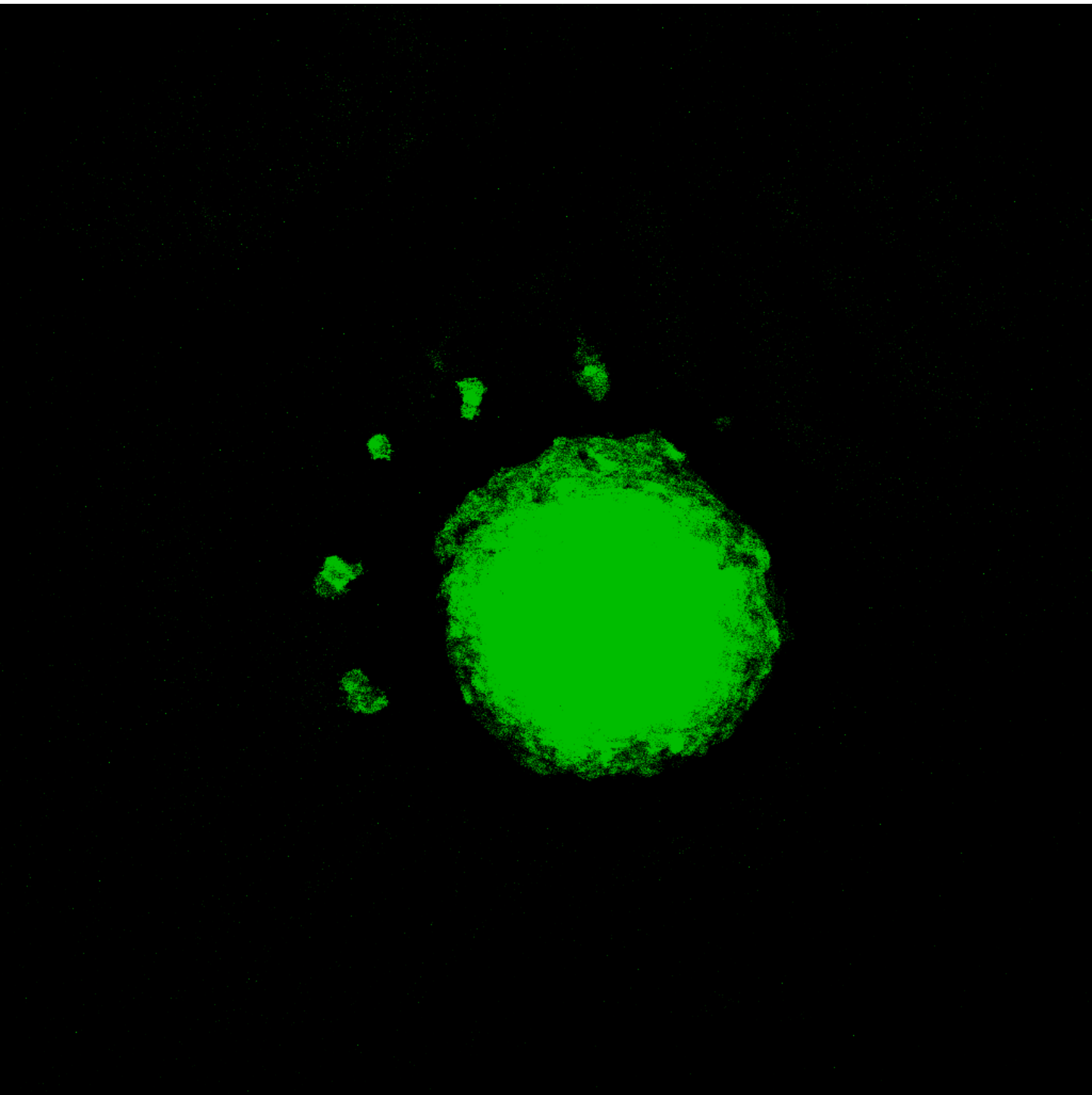

D  
GFP-MDGI d5

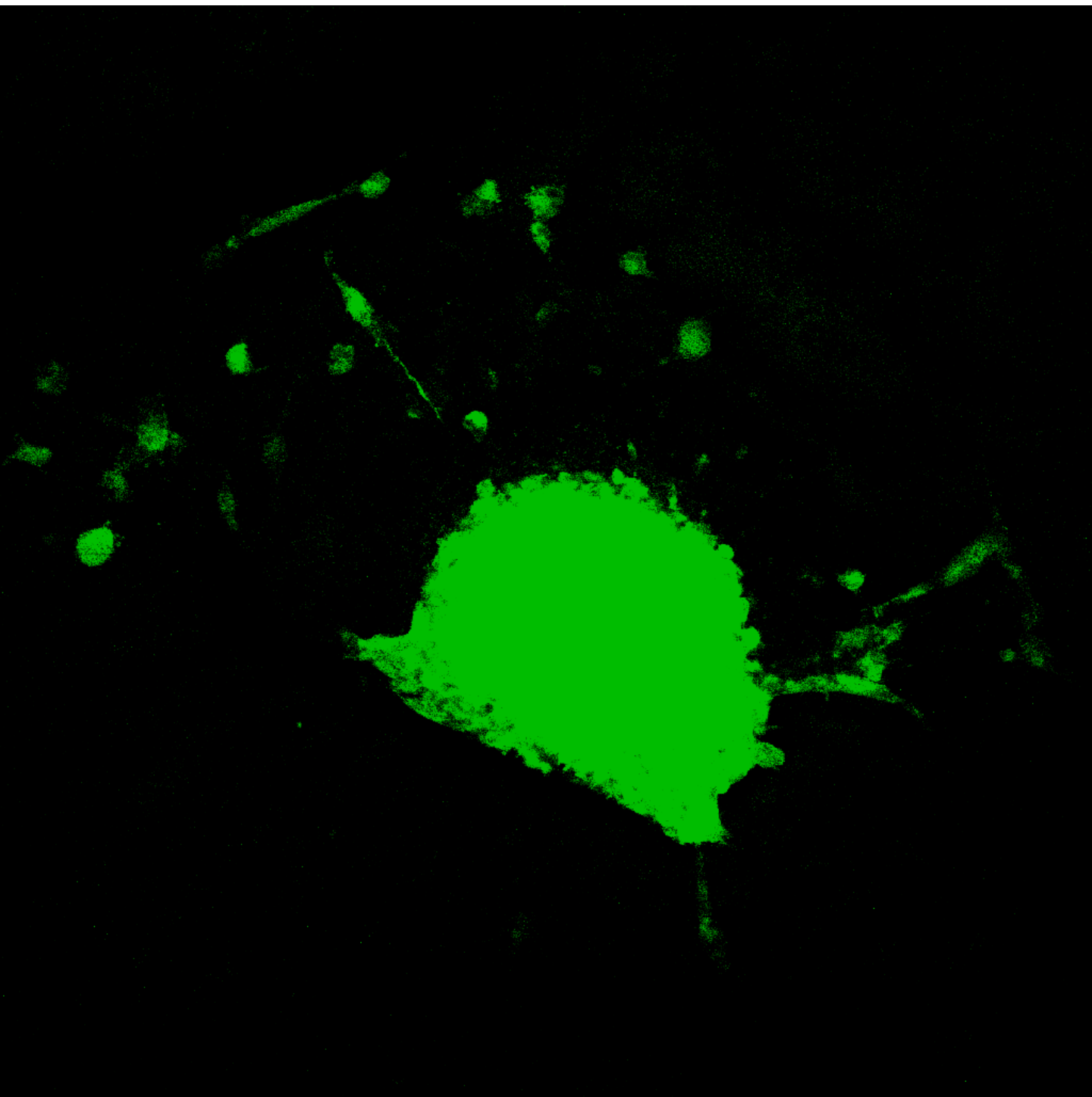

Supplement: Supplementary file 3 — Source Data for Expanded View and Appendix [file EMMM-11-e9034-s009.zip › 9034_EV_Appendix_SD/Figure_EV1_Source.pdf]

# Source data for Expanded view Fig EV2

A

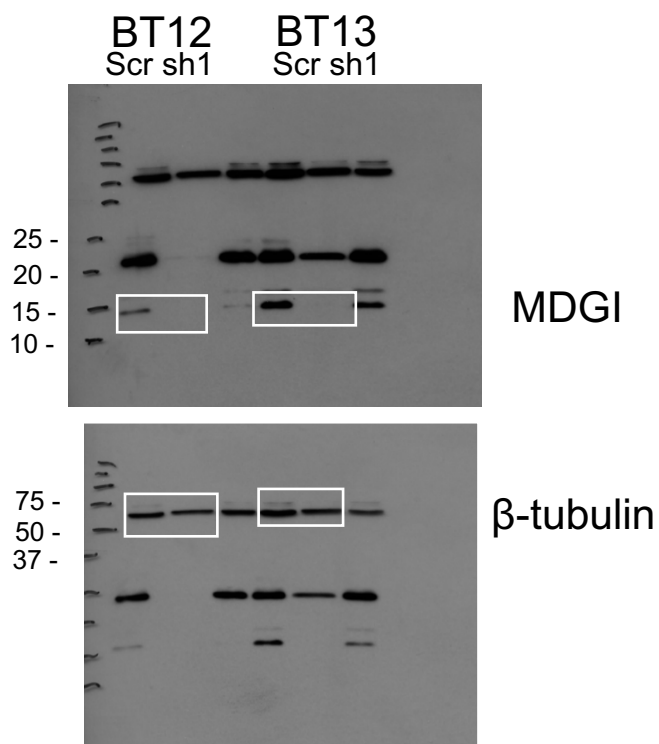

B

BT12  
Scr sh2

BT13  
Scr sh2

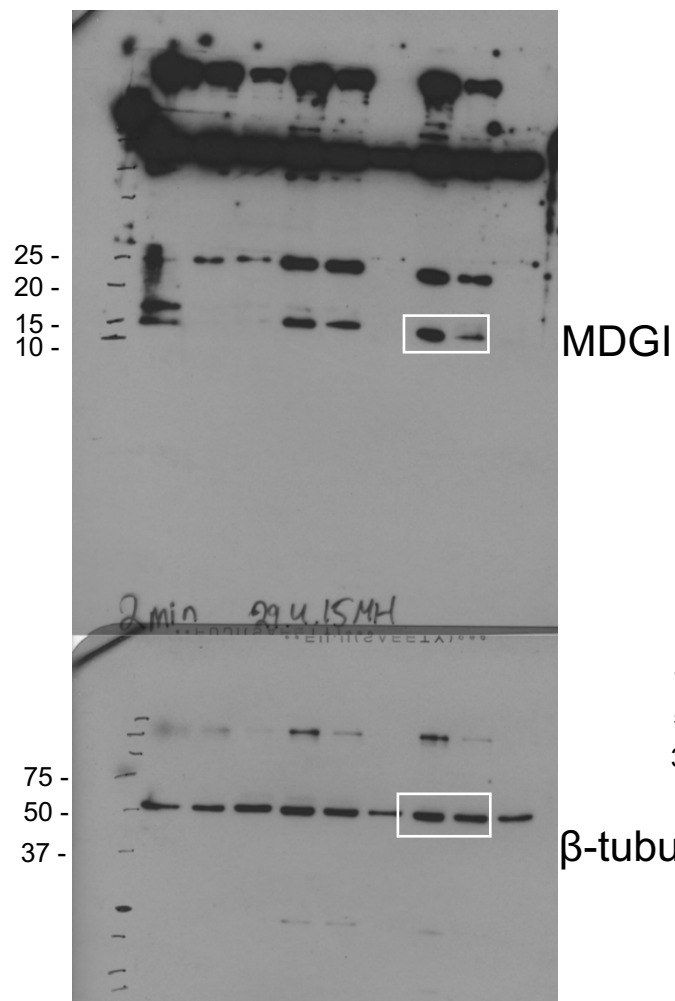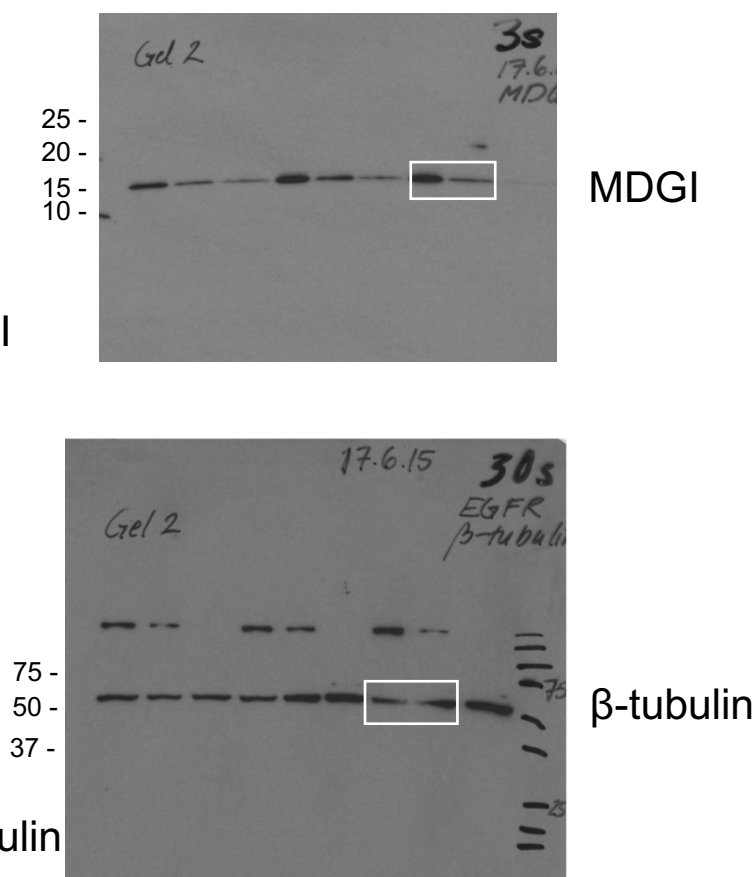

Supplement: Supplementary file 3 — Source Data for Expanded View and Appendix [file EMMM-11-e9034-s009.zip › 9034_EV_Appendix_SD/Figure_EV2_Source.pdf]

10x

40x

A

Control

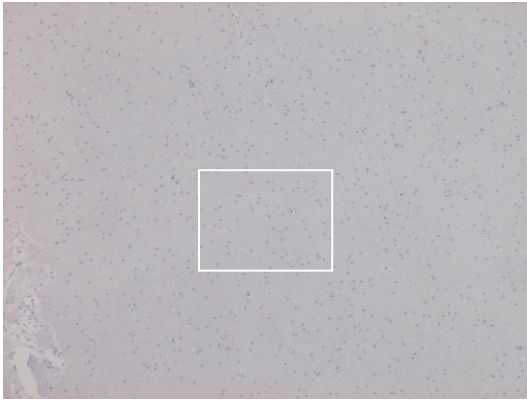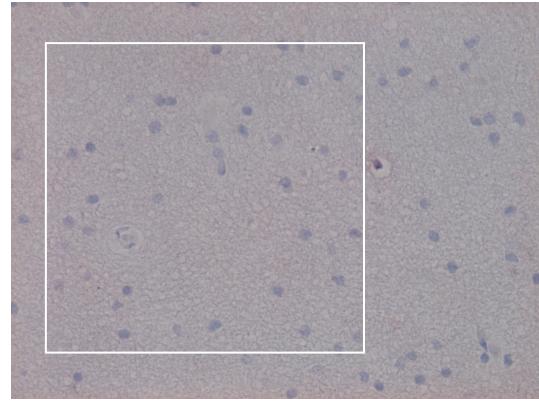

Grade II

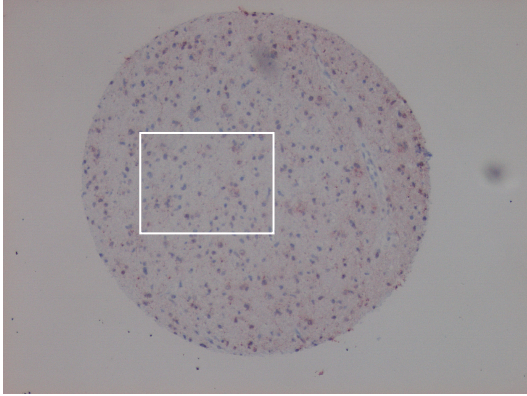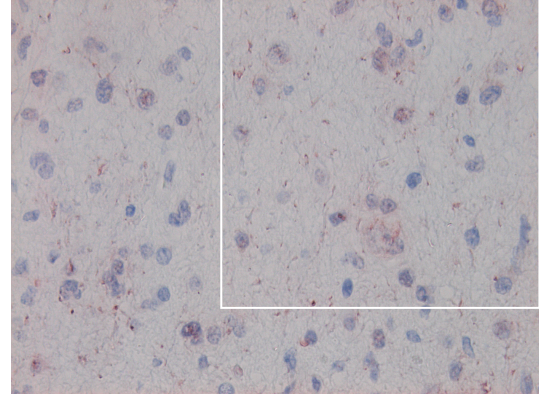

Grade III

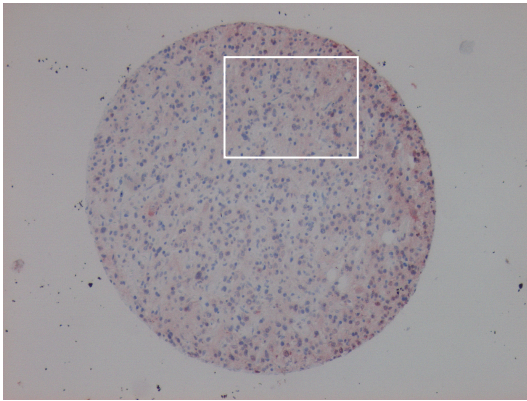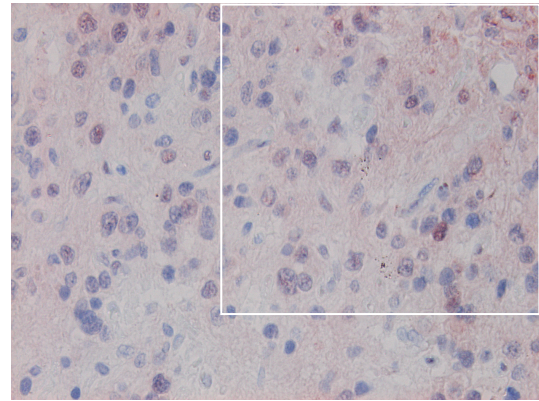

Grade IV

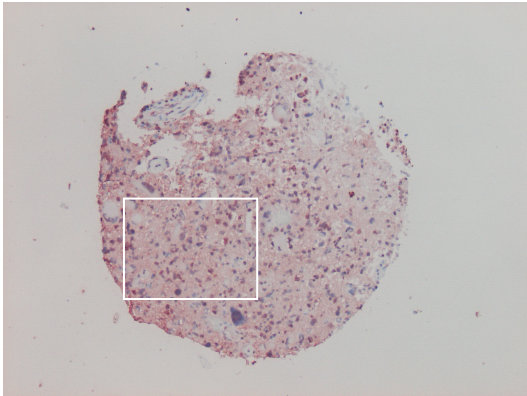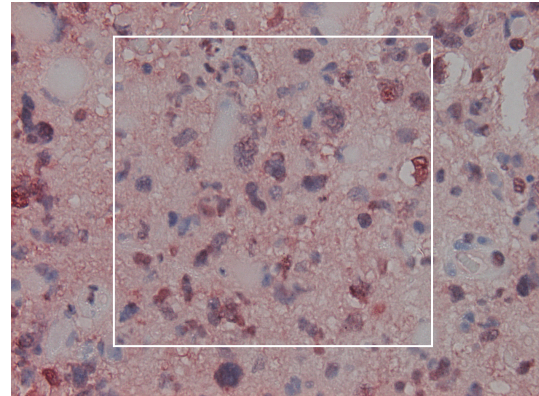

F

Glioma stem cells

Adherent

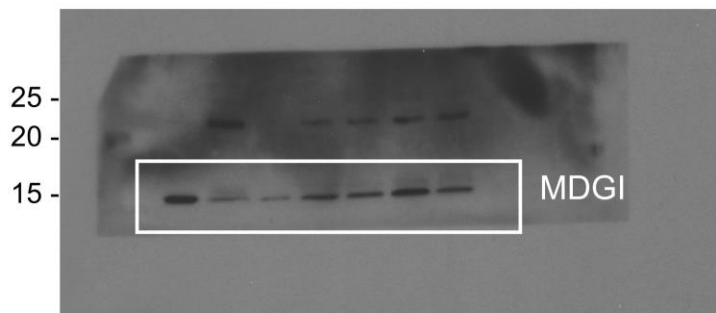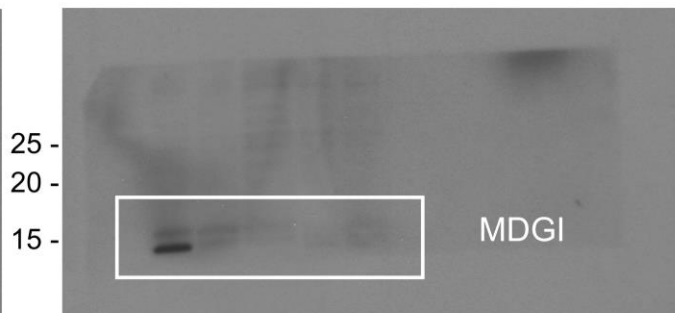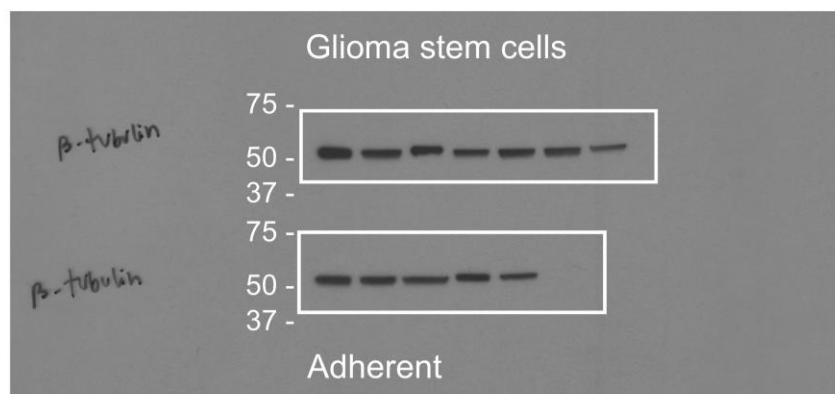

G

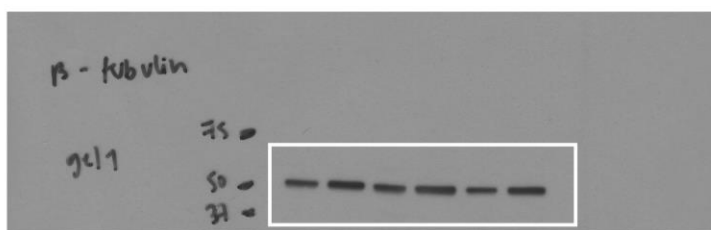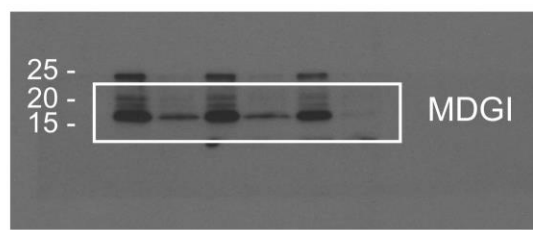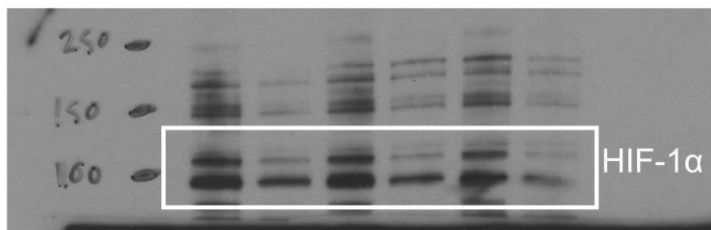

H

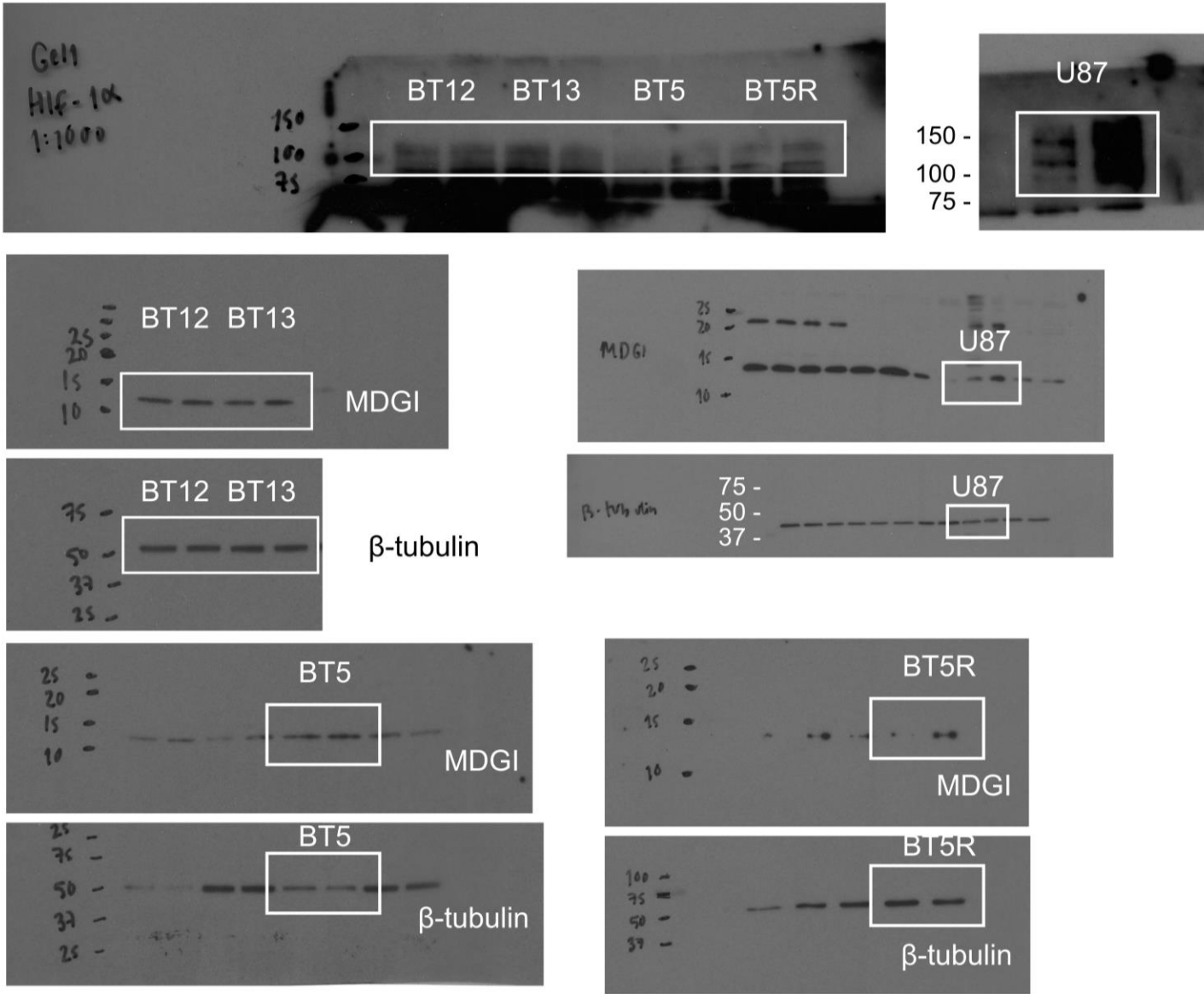

Supplement: Supplementary file 5 — Source Data for Figure 1 [file EMMM-11-e9034-s003.pdf]

Source data for Fig 2

A

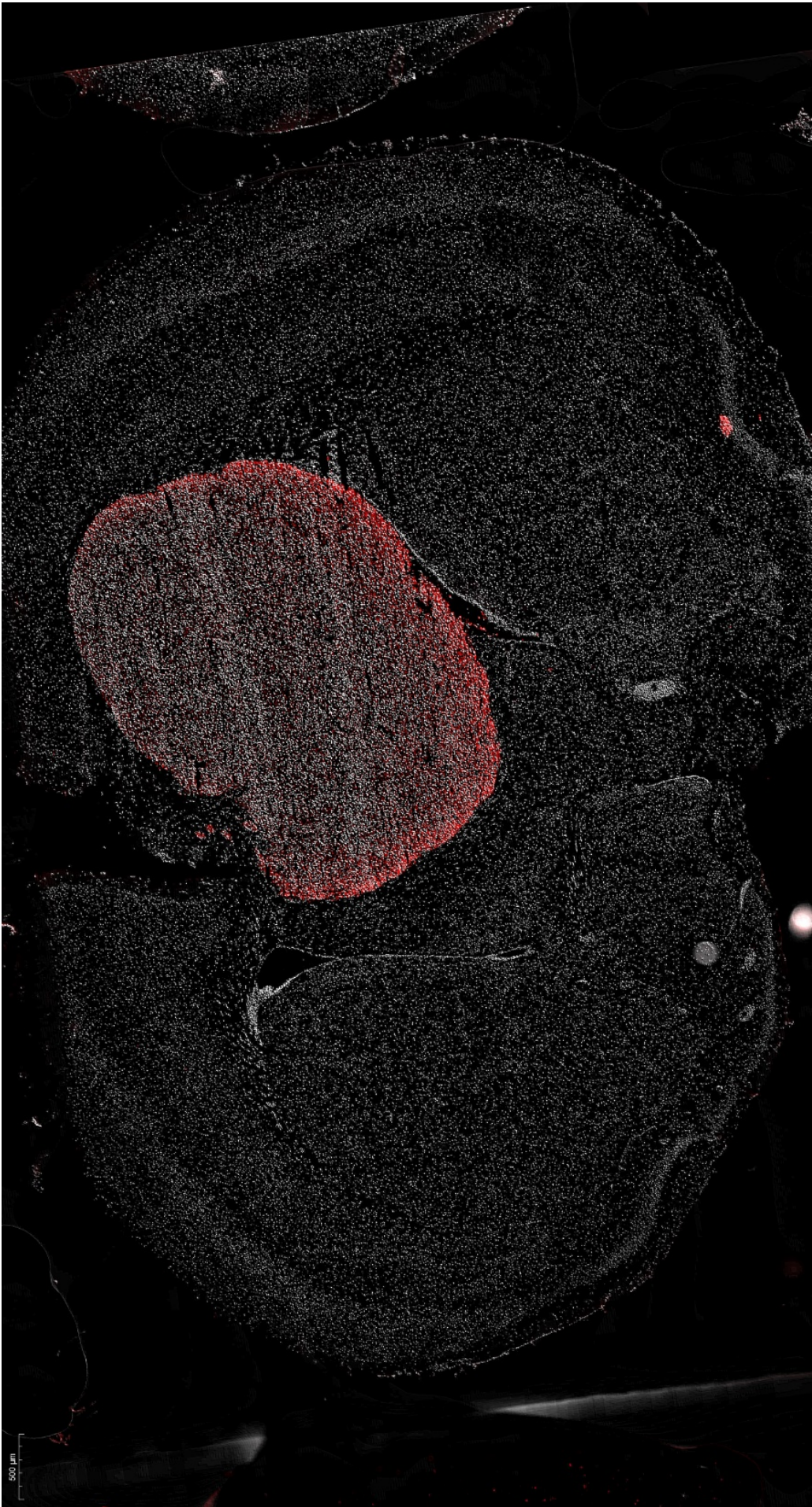

B

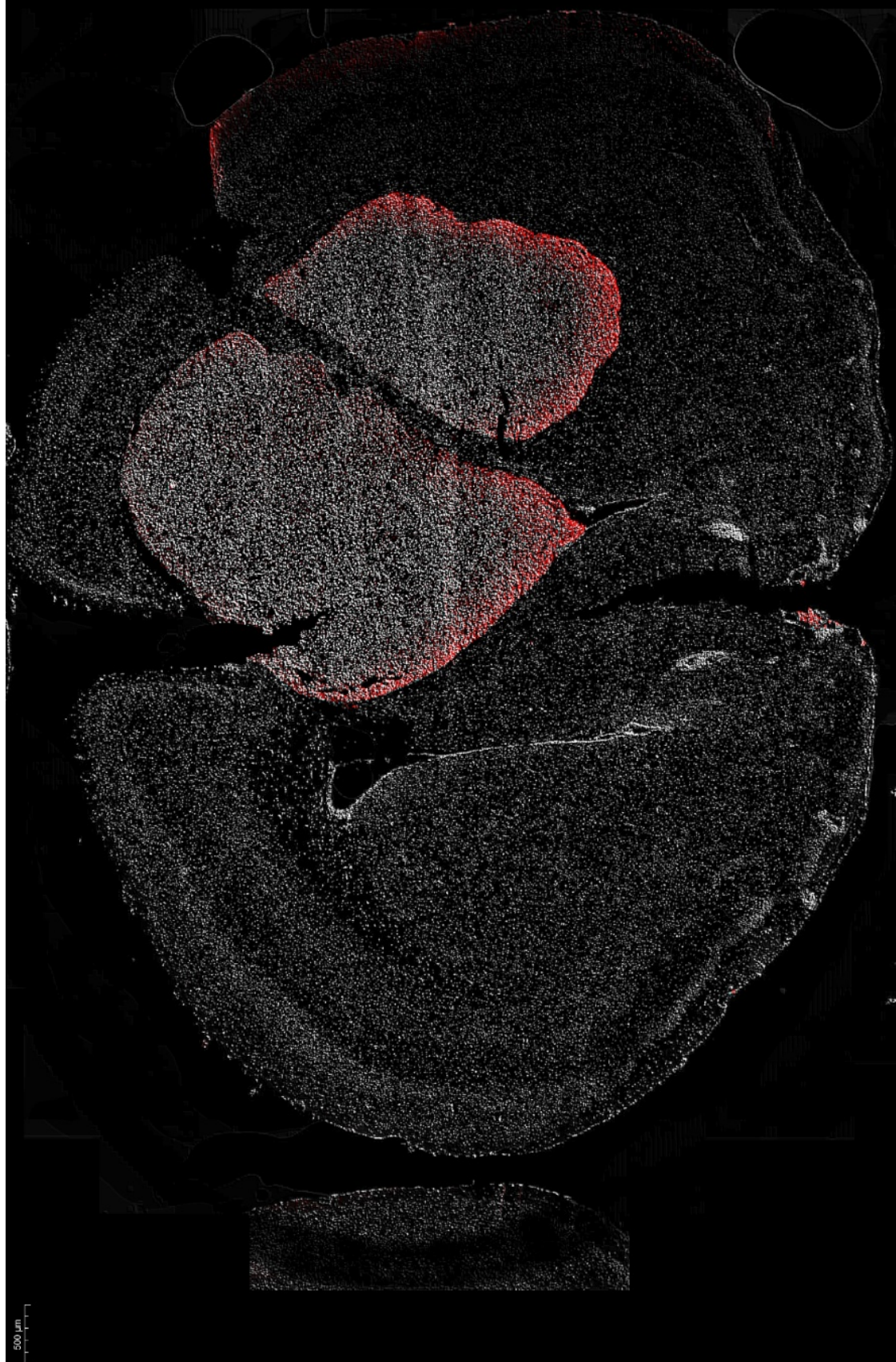

C

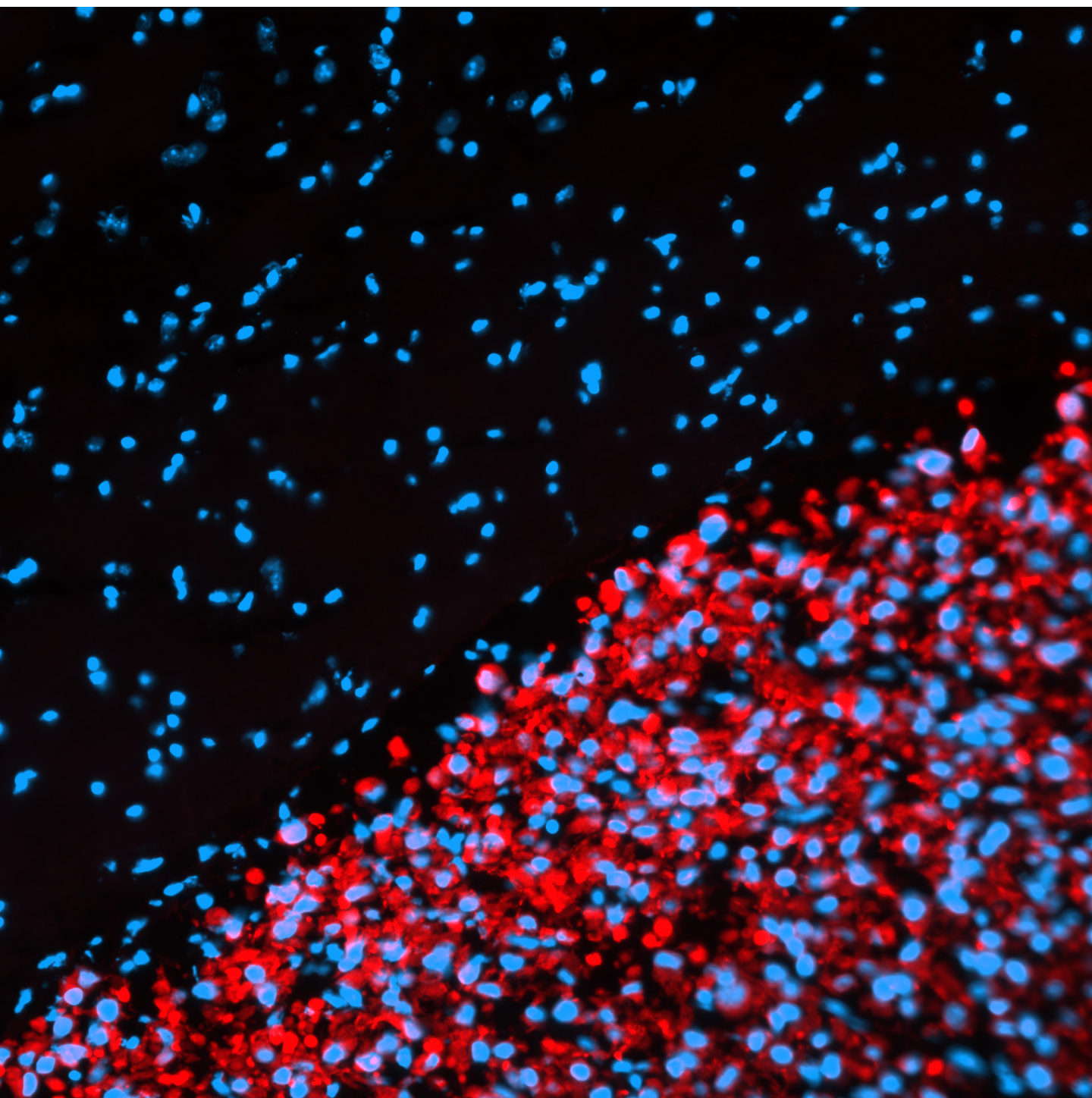

D

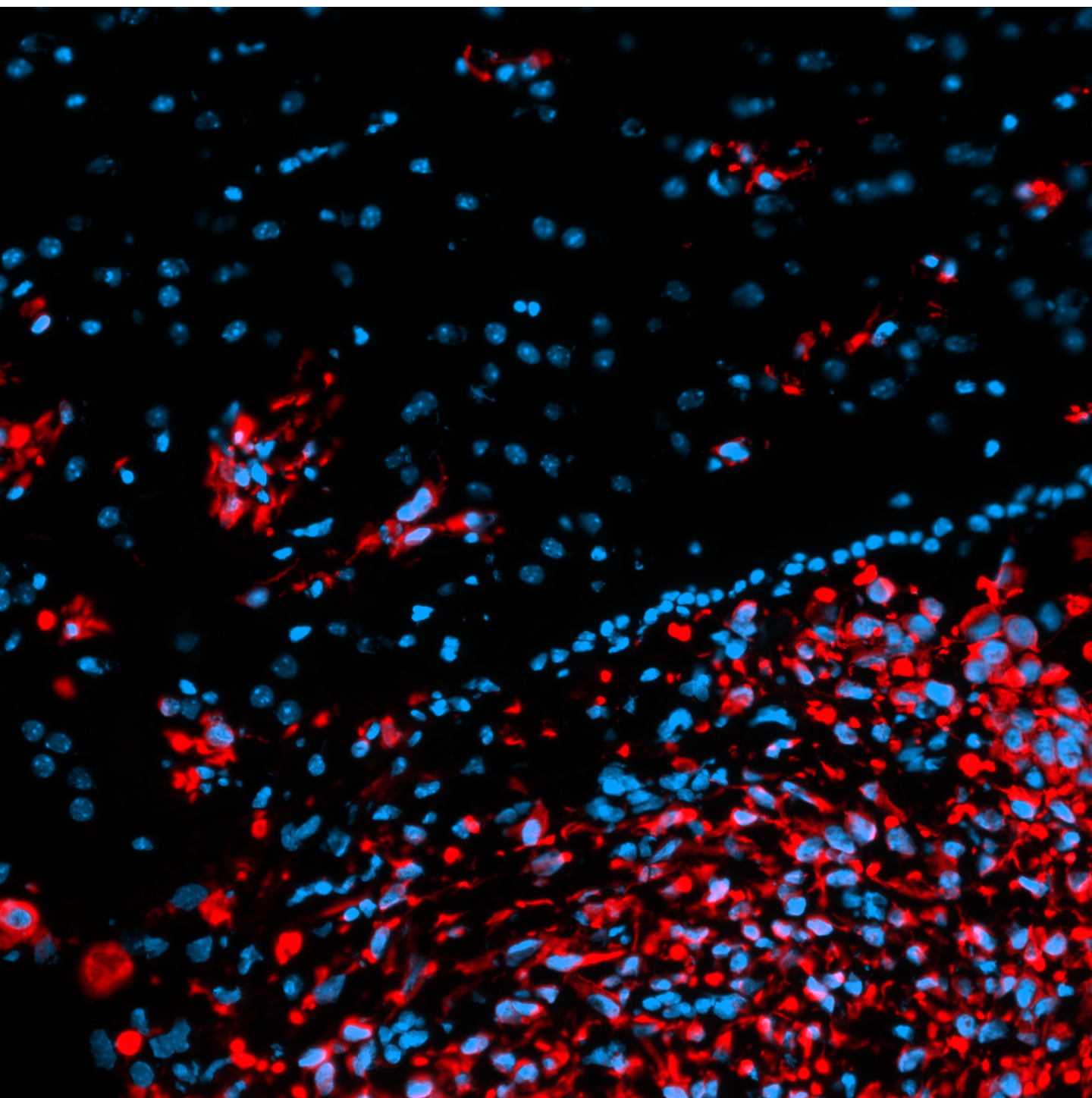

E

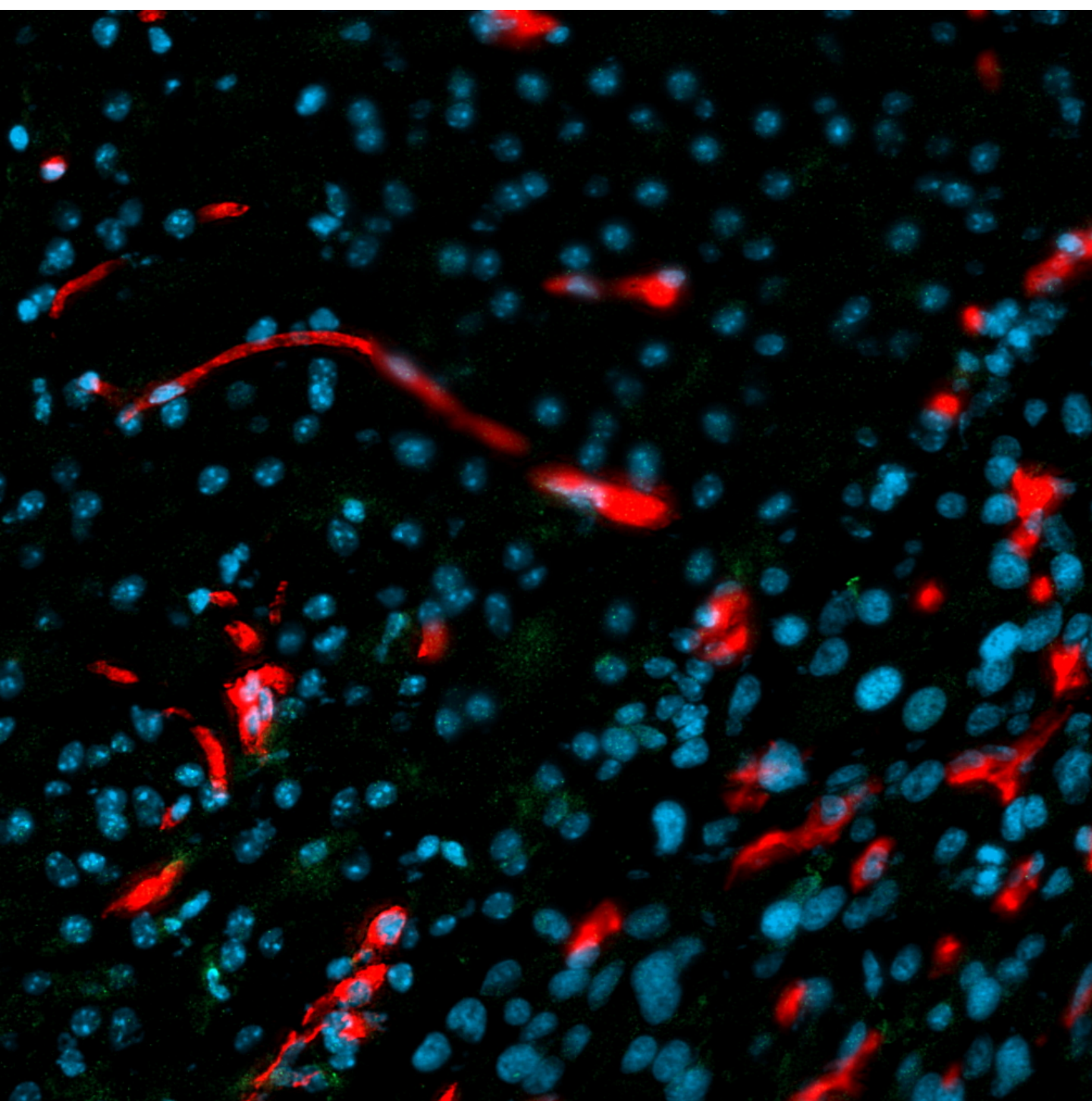

F

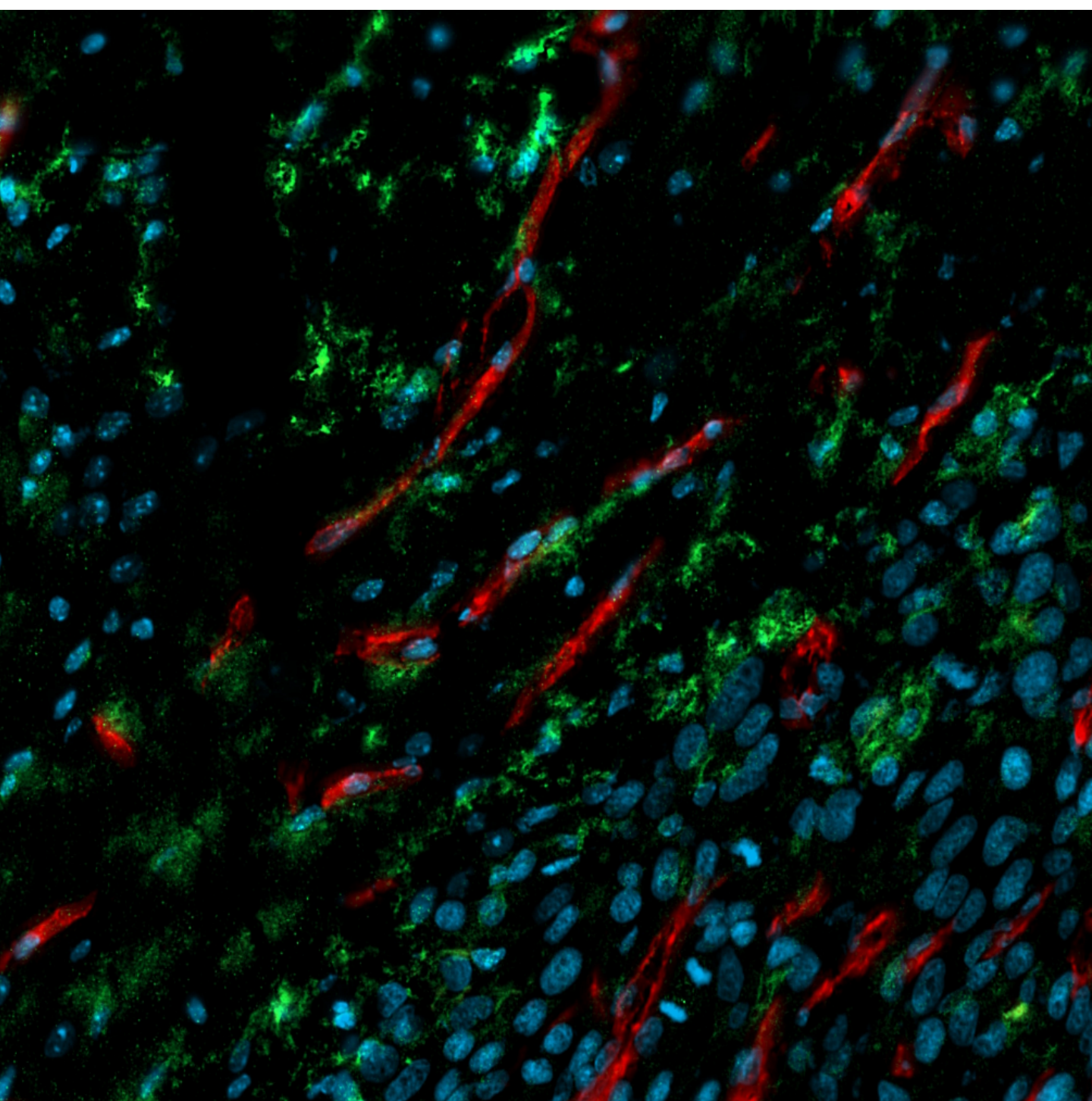

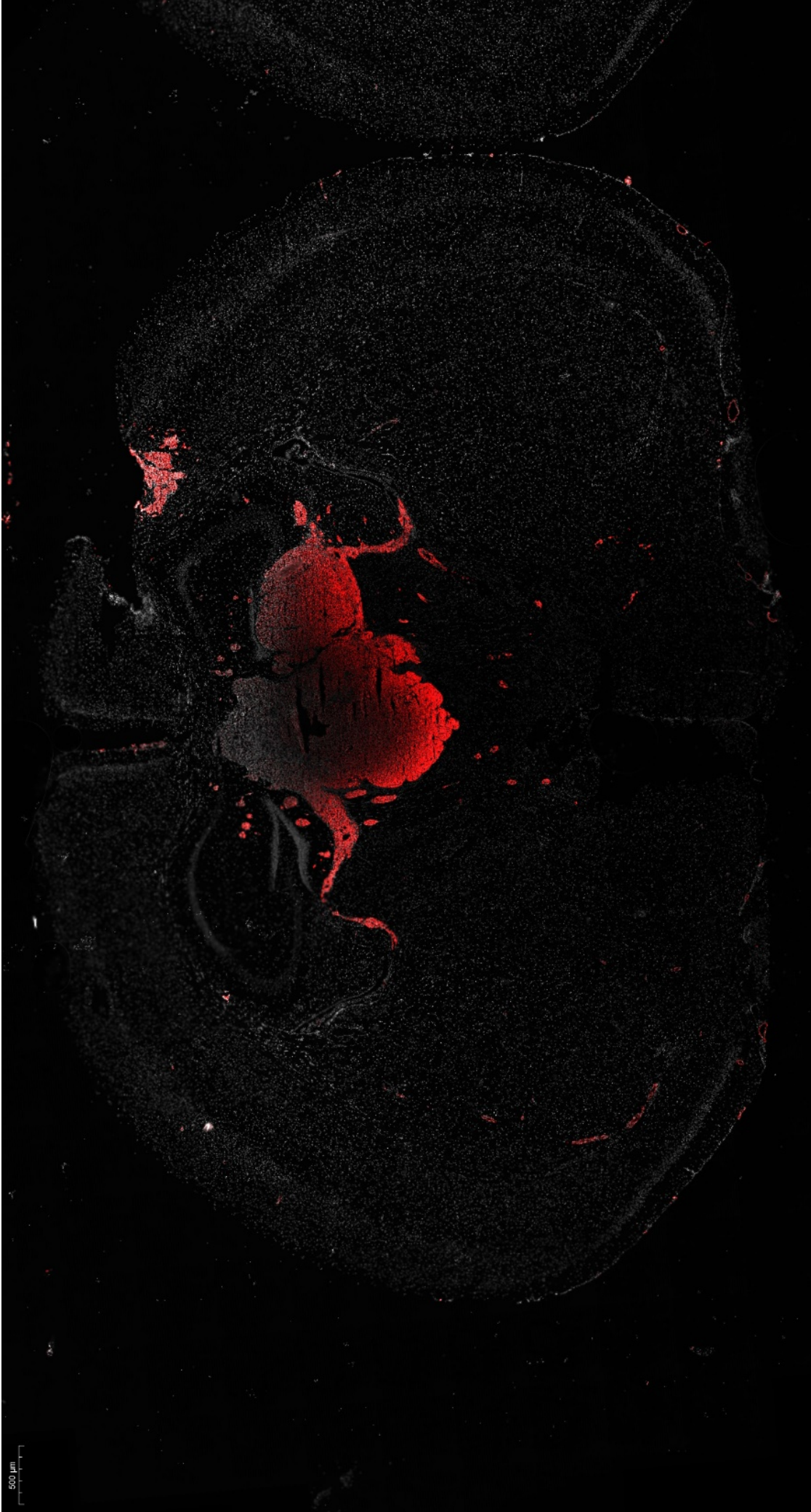

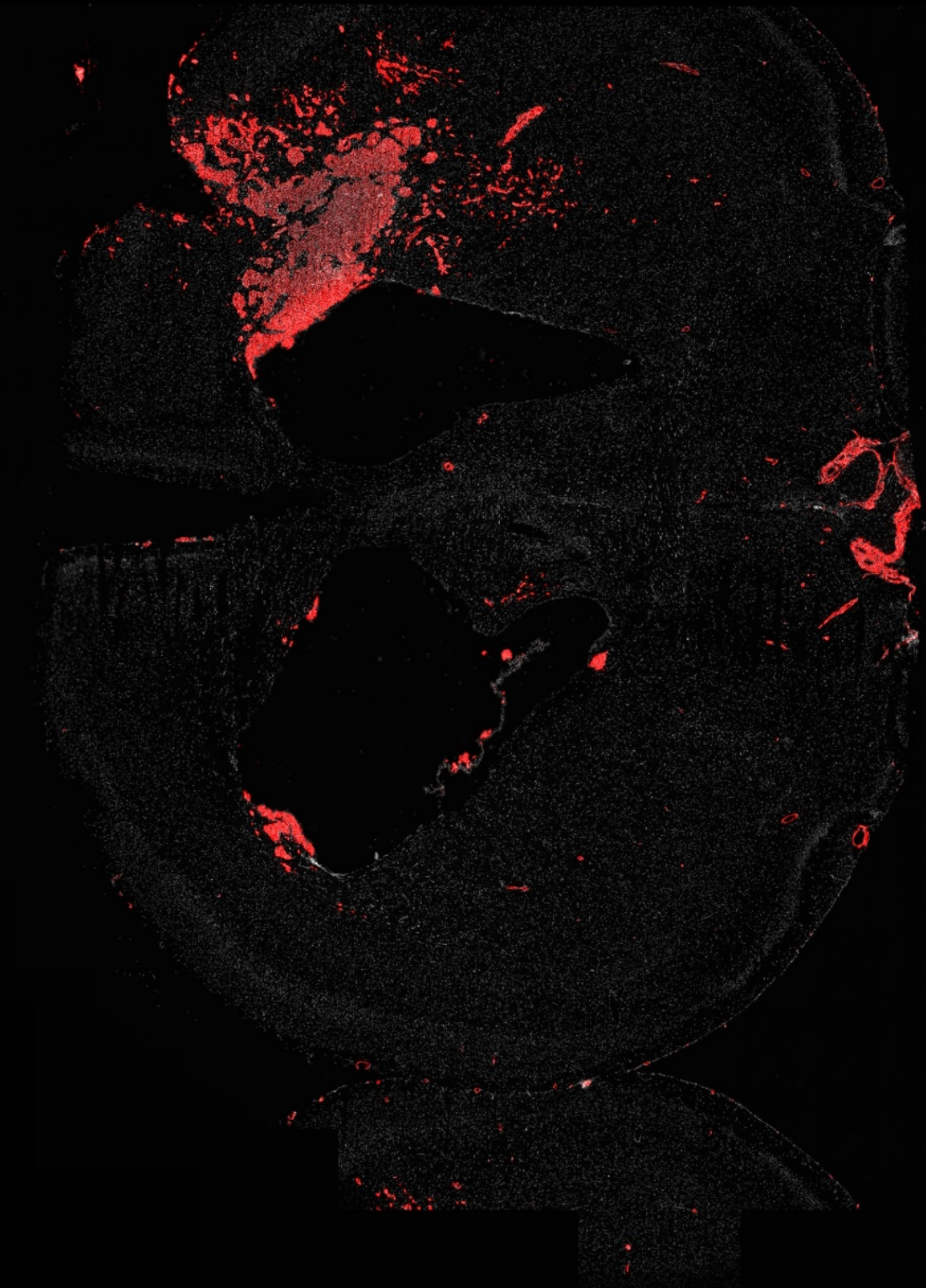

500  $\mu$ m

K

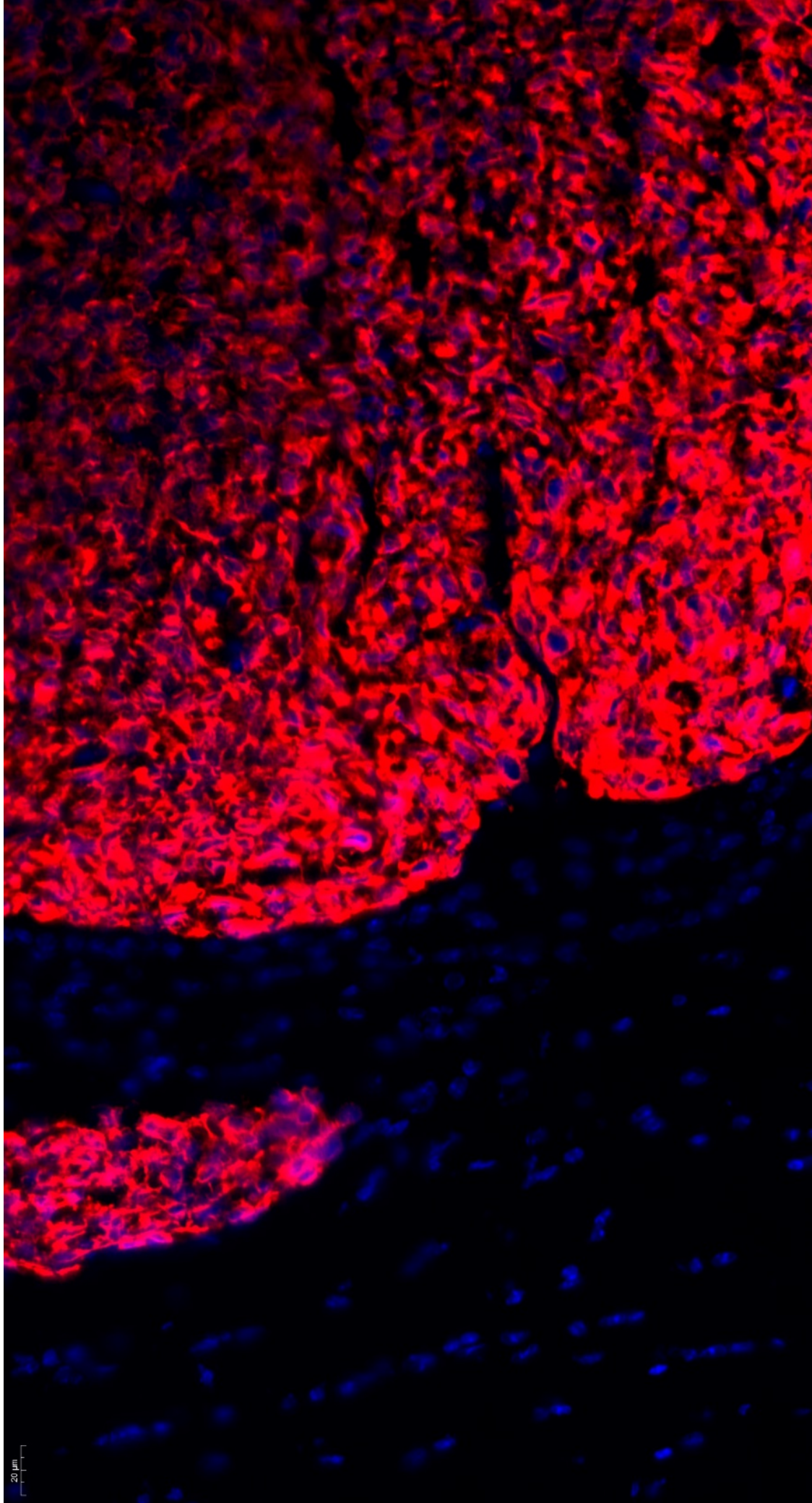

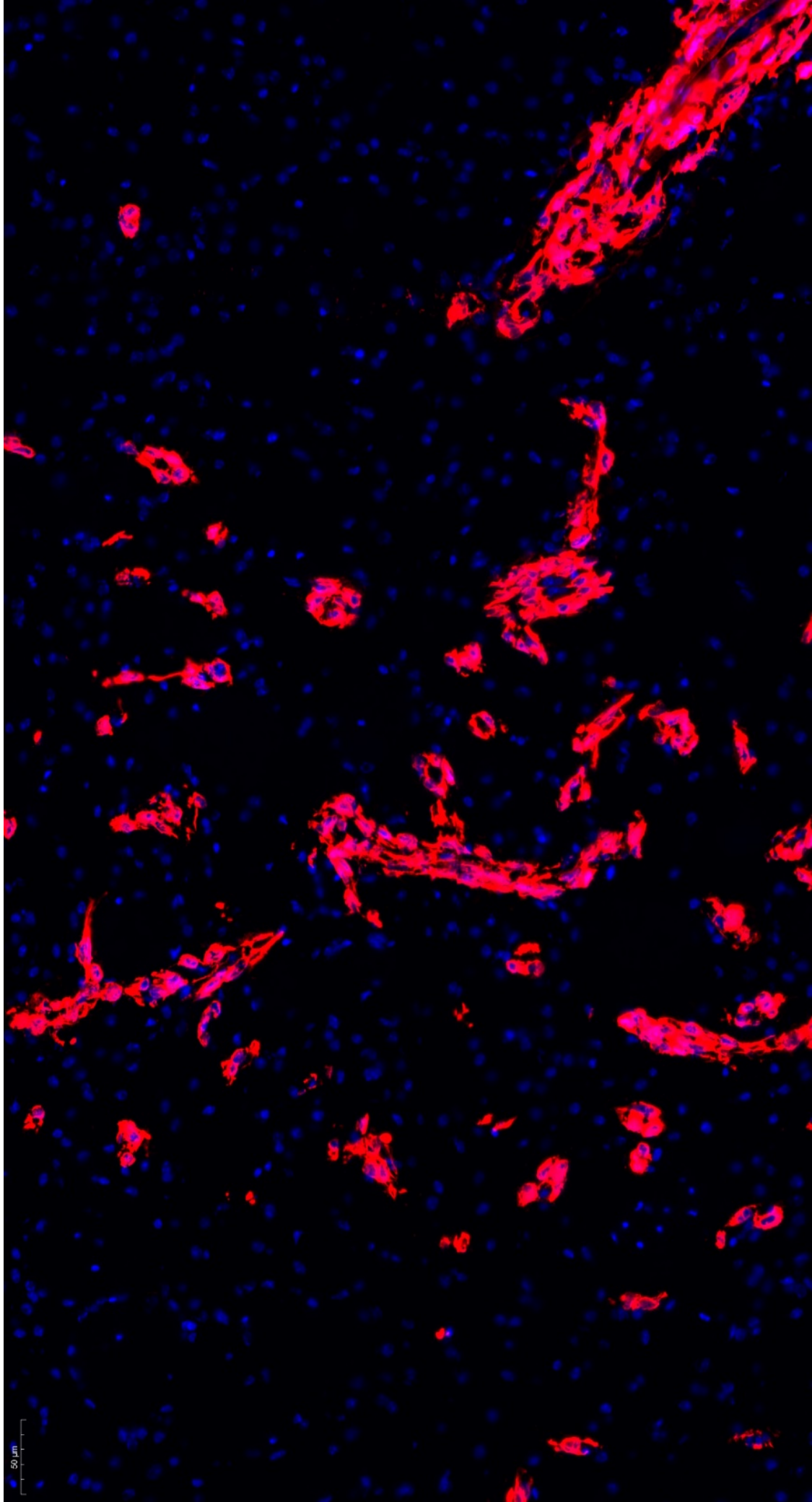

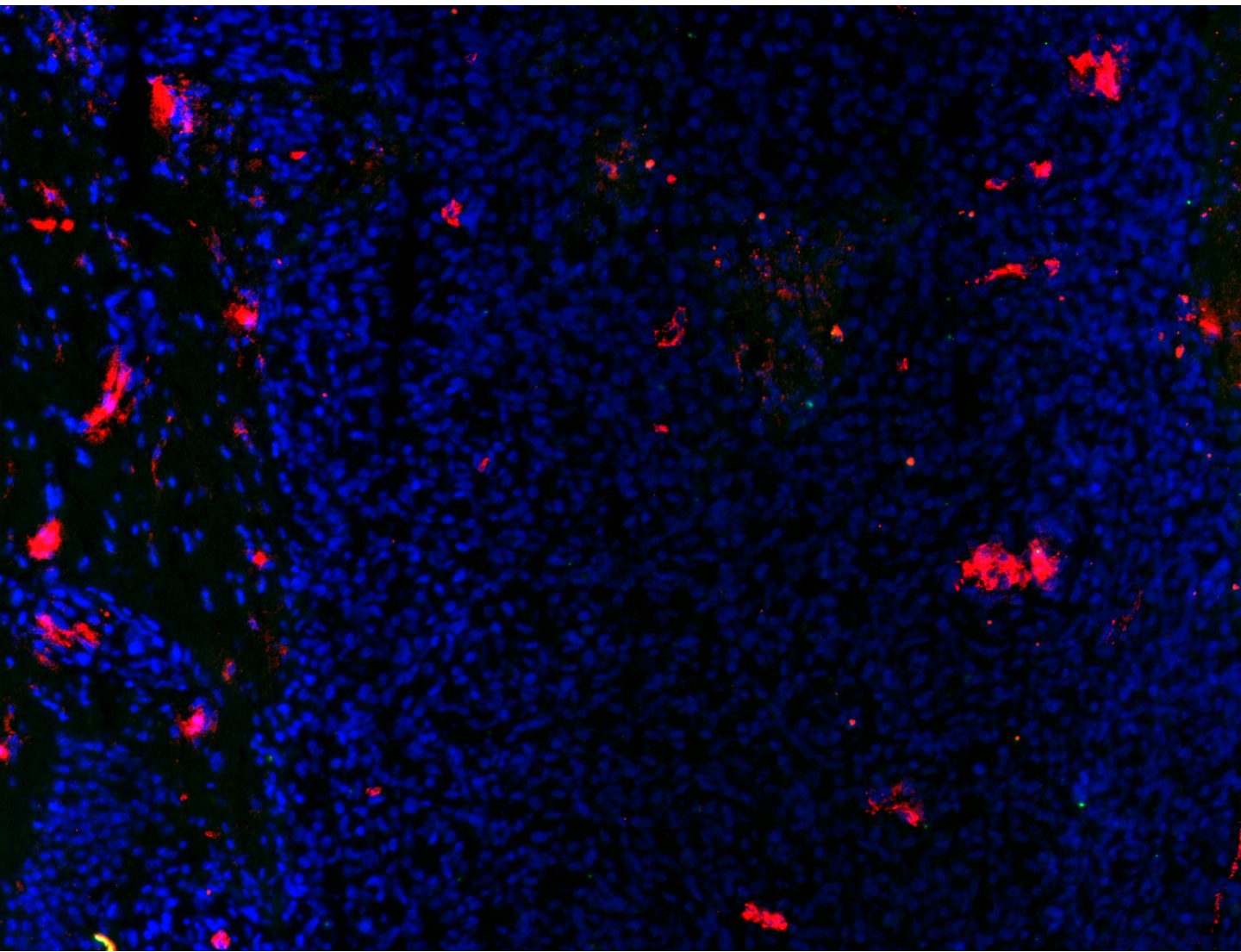

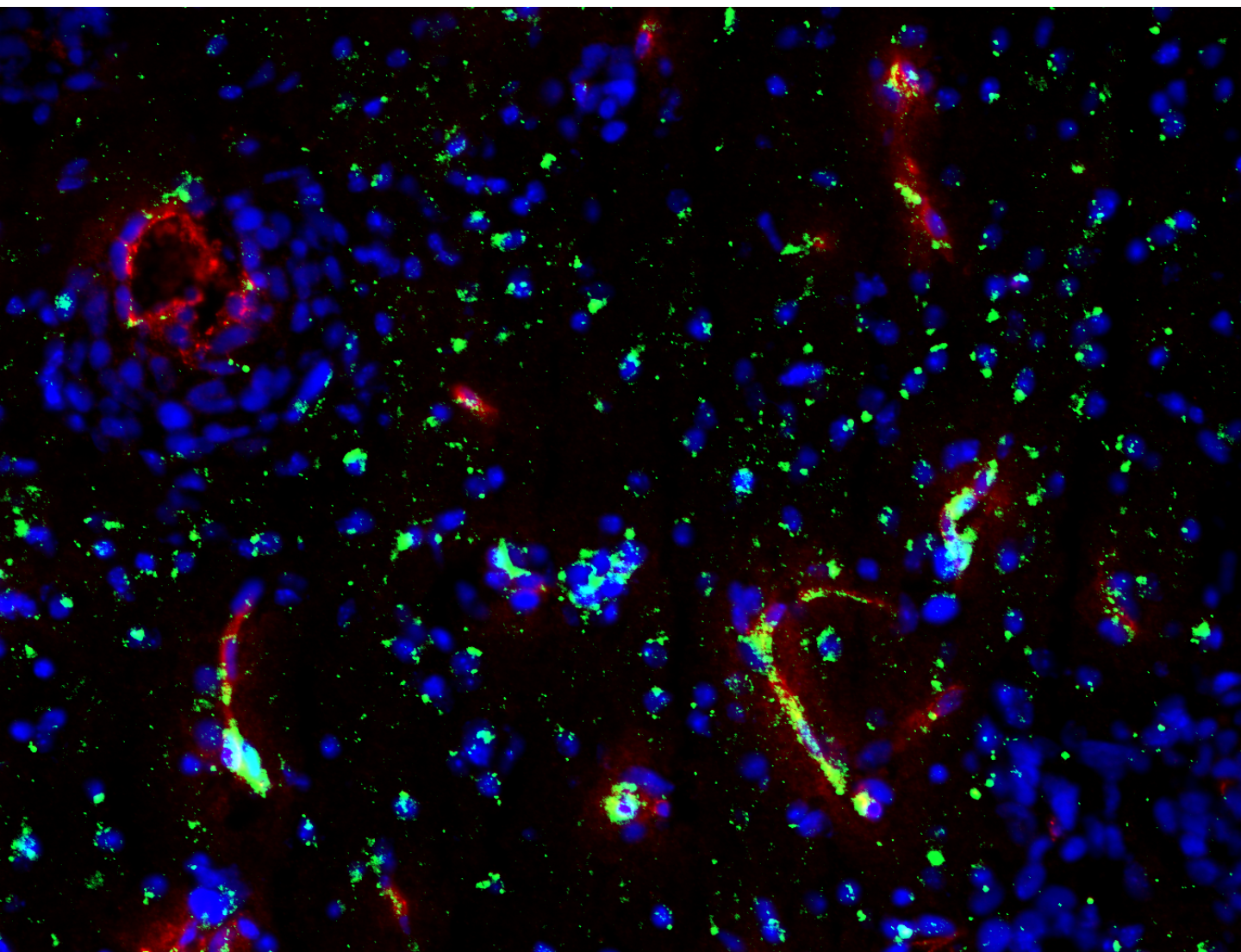

Supplement: Supplementary file 6 — Source Data for Figure 2 [file EMMM-11-e9034-s004.pdf]

C

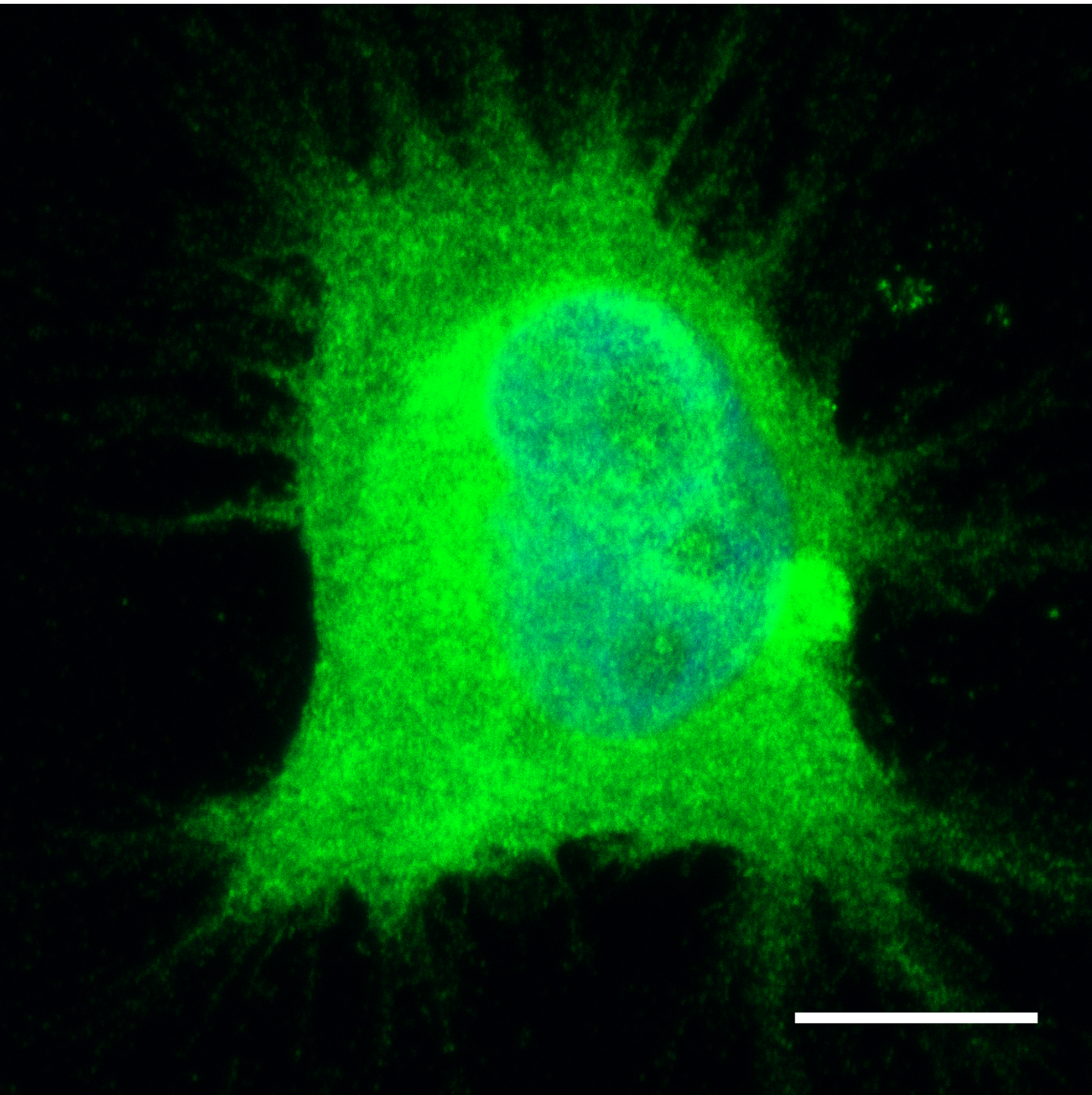

D

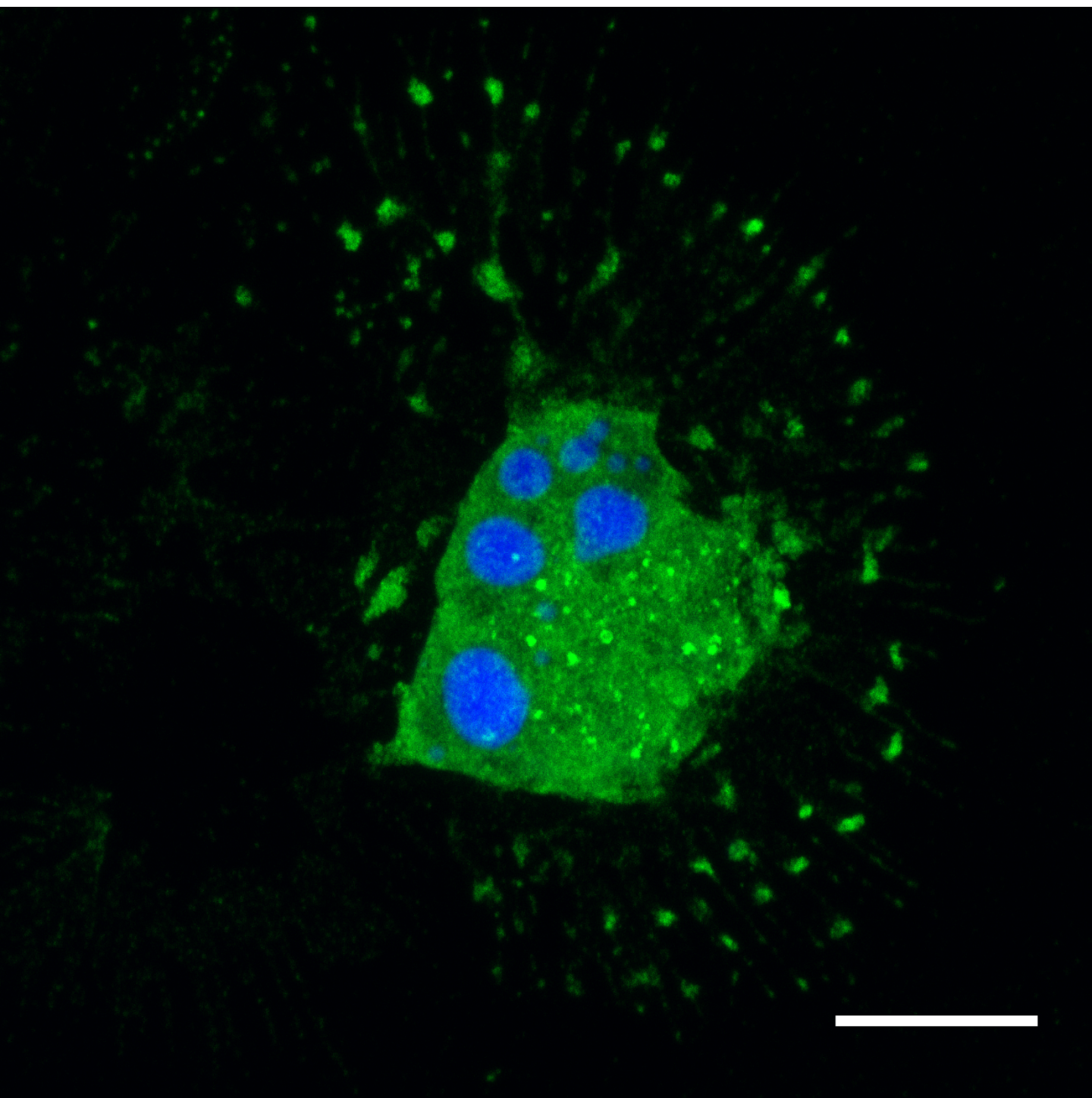

E

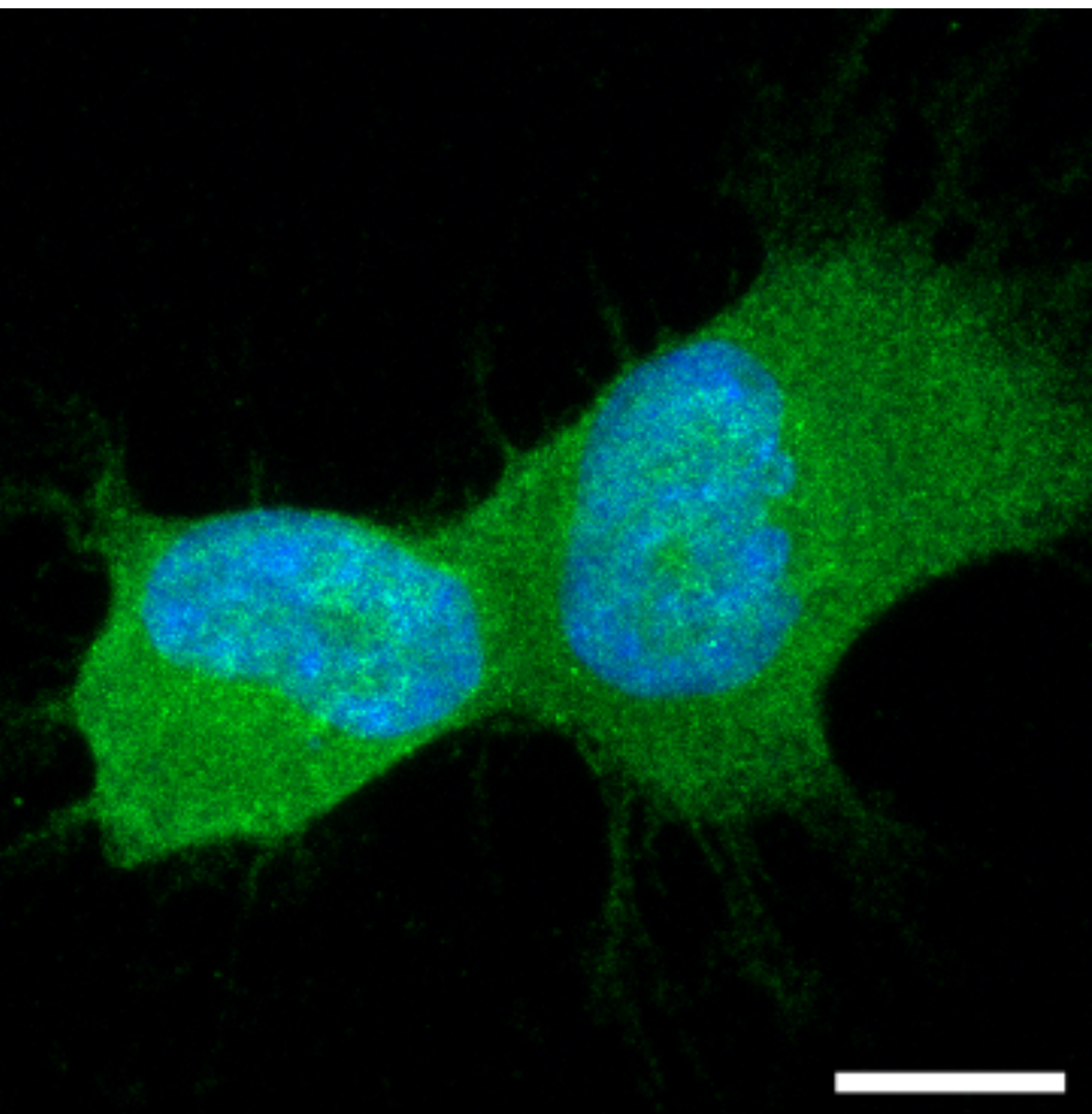

F

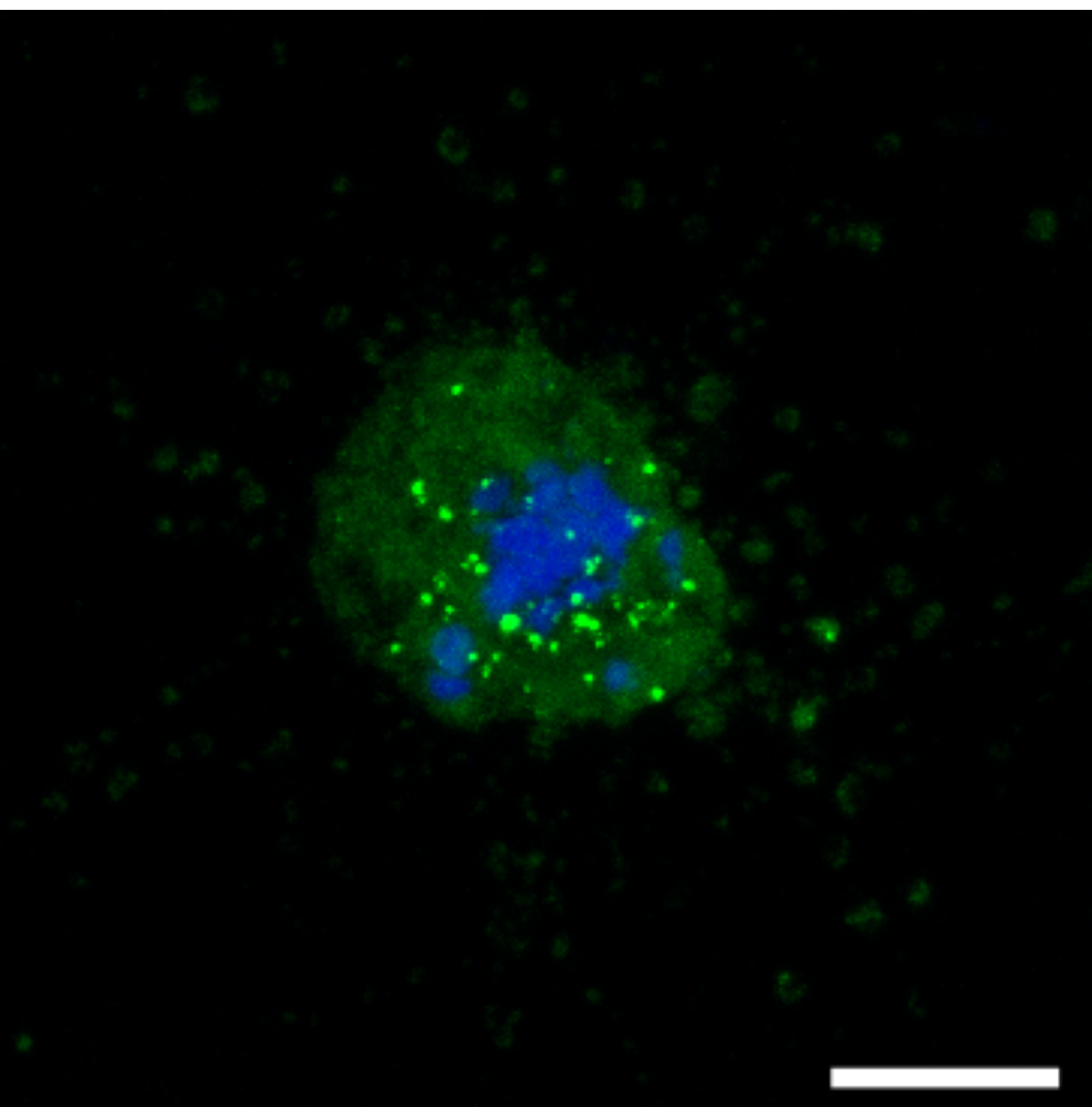

G

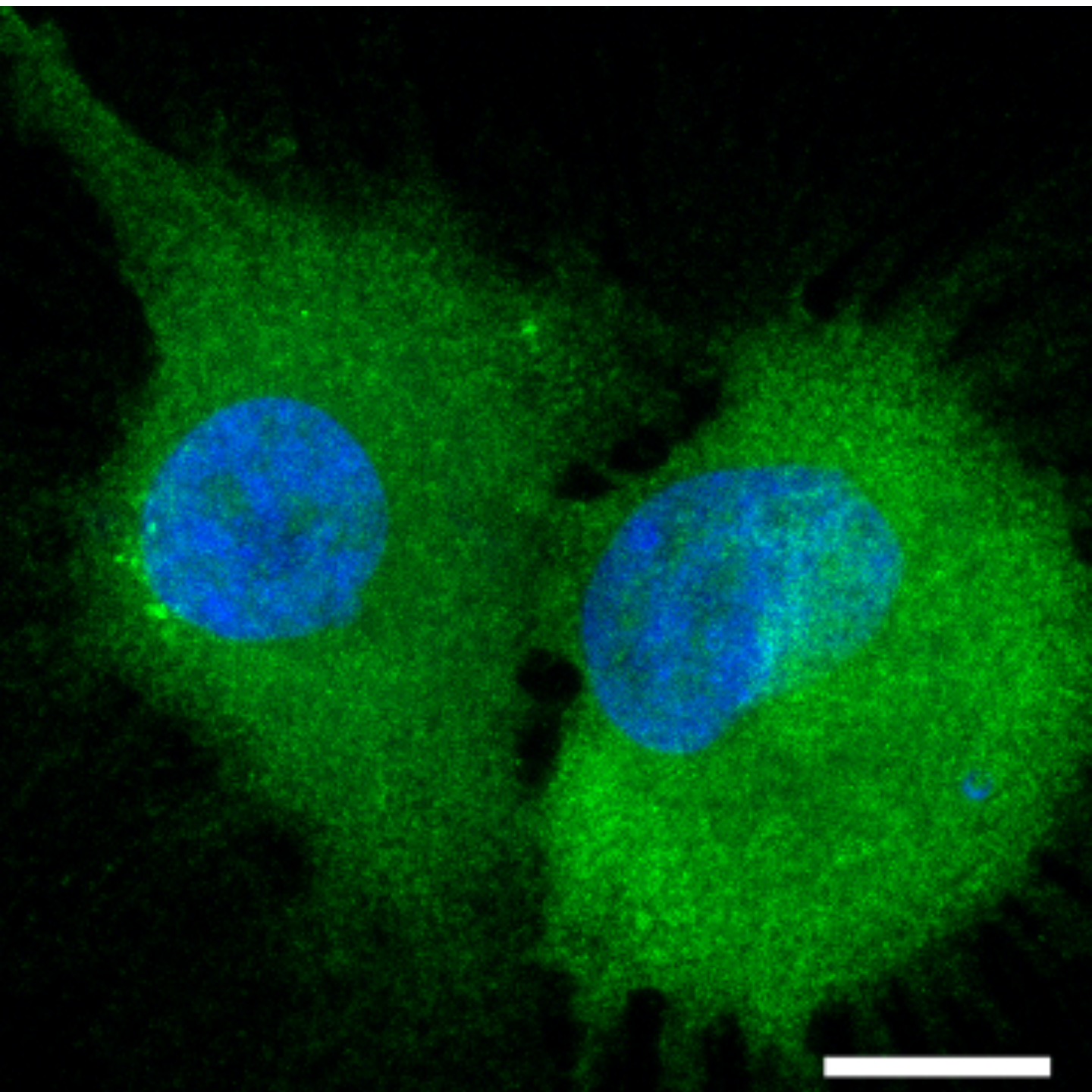

H

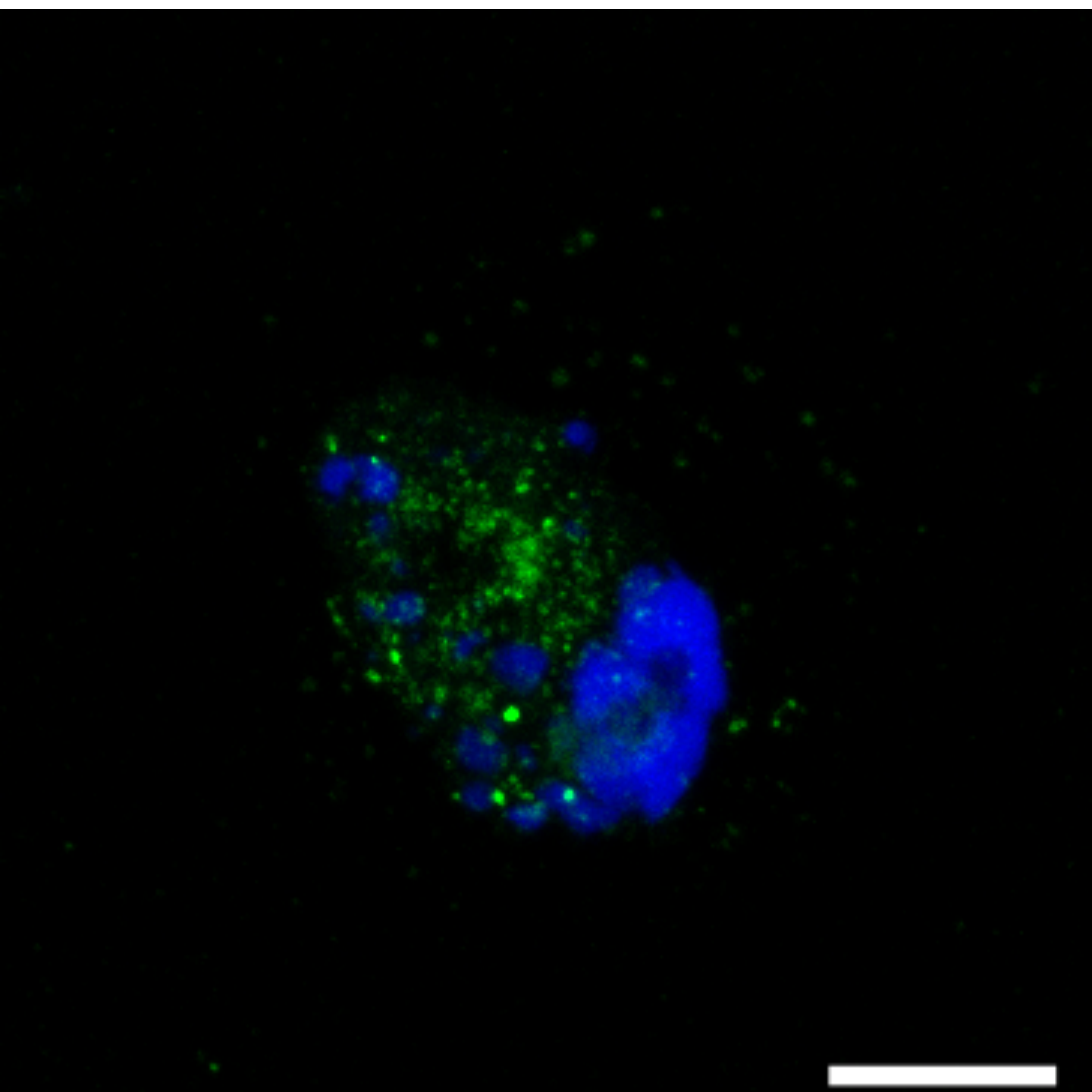

Supplement: Supplementary file 8 — Source Data for Figure 4 [file EMMM-11-e9034-s006.pdf]

C BT12 control

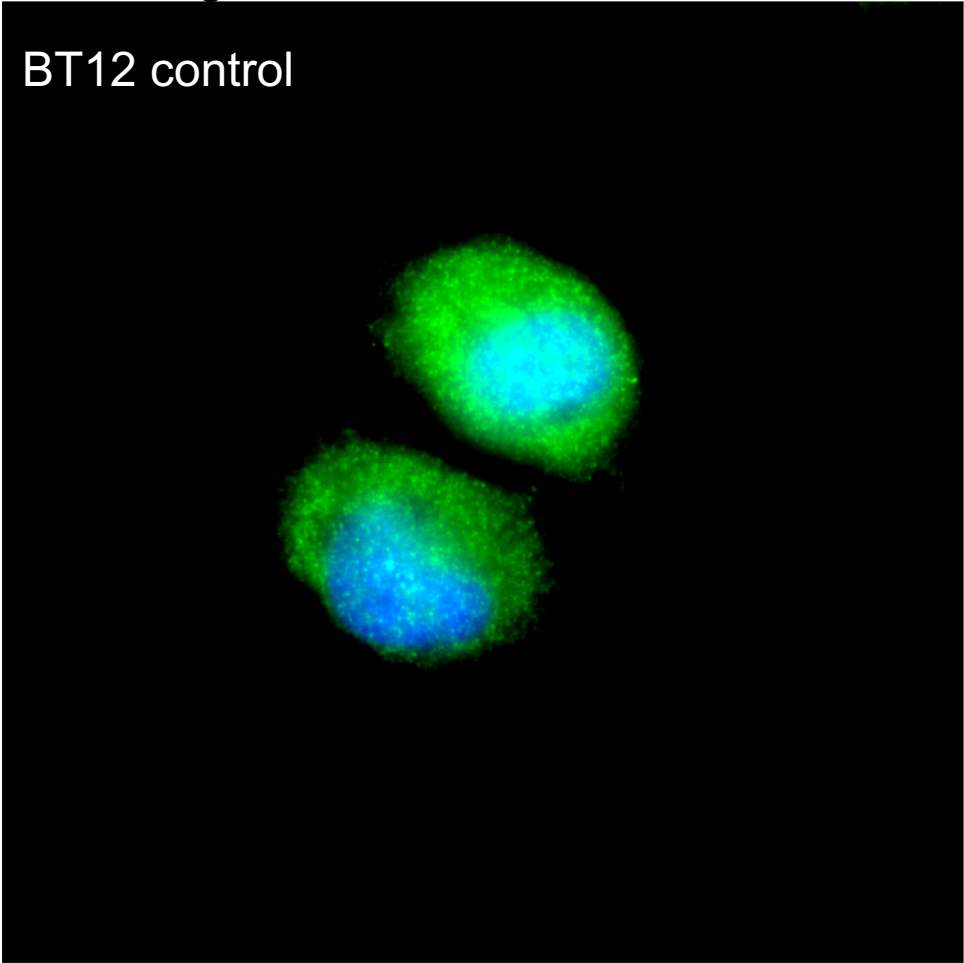

BT12 clemastine

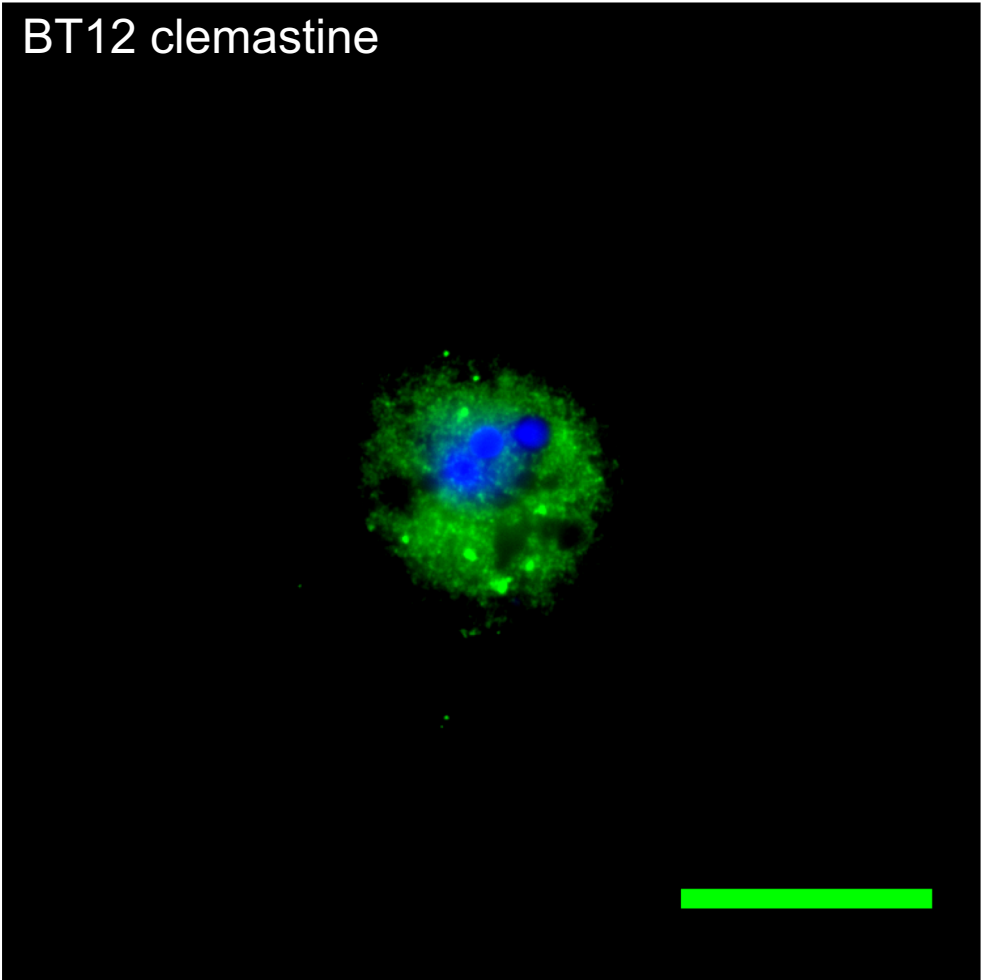

C  
BT13 control

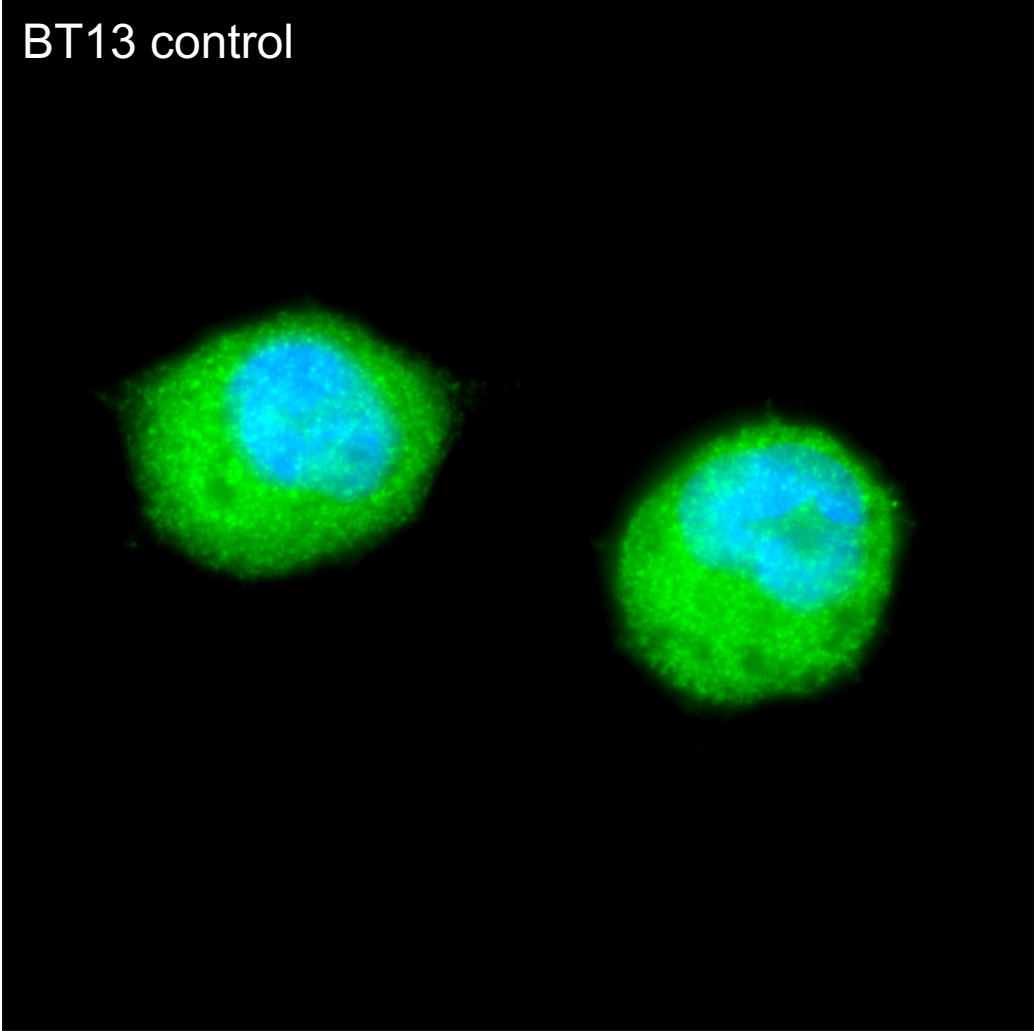

BT13 clemastine

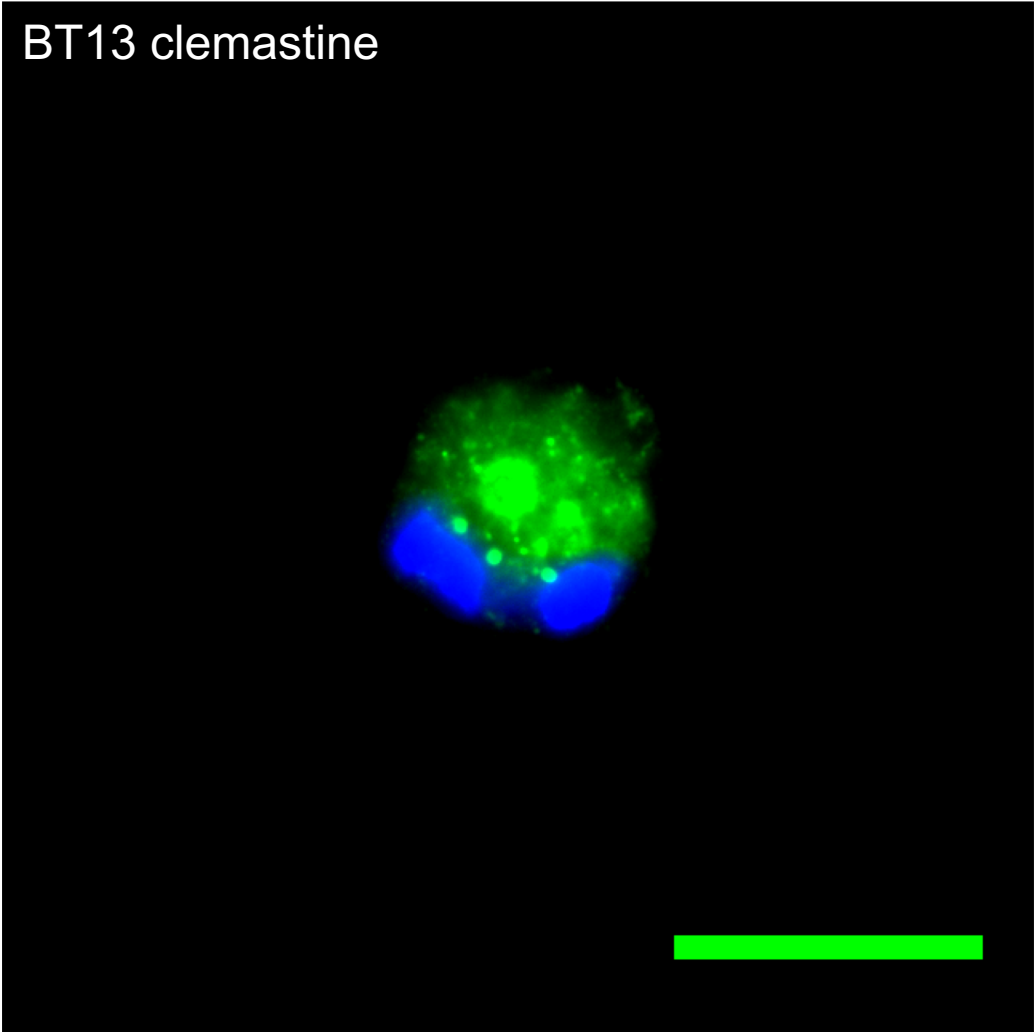

C      ZH305 control

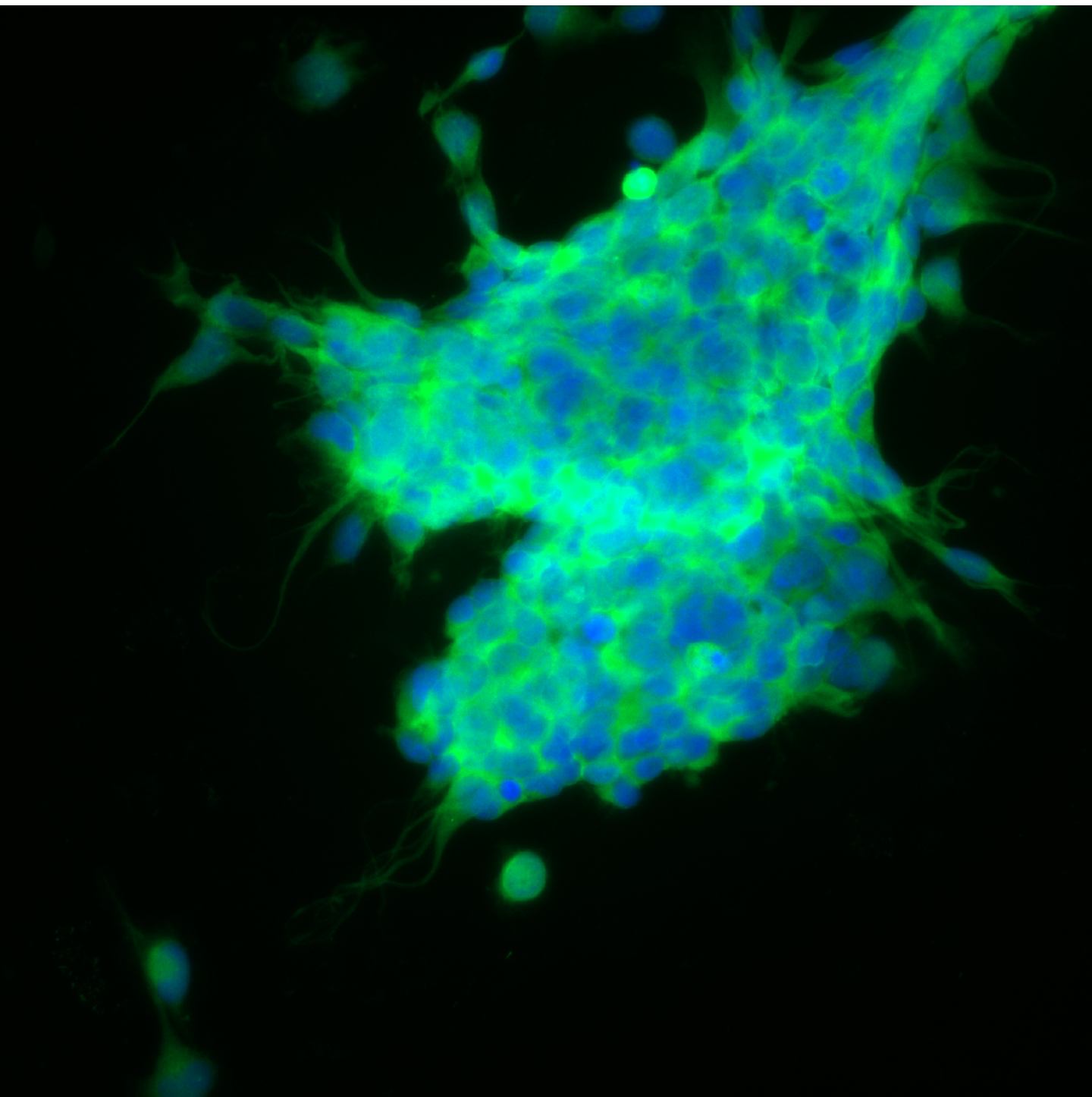

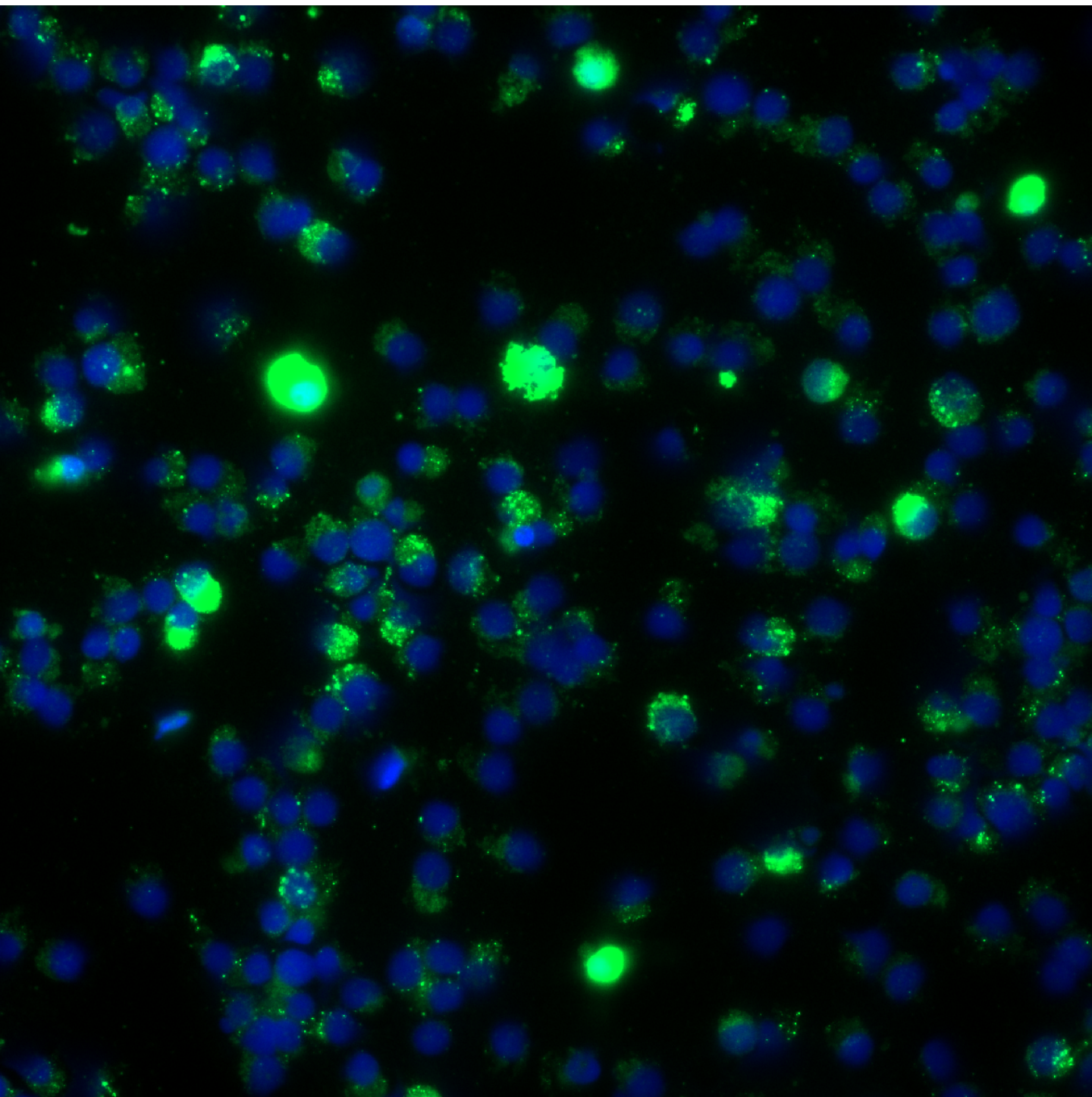

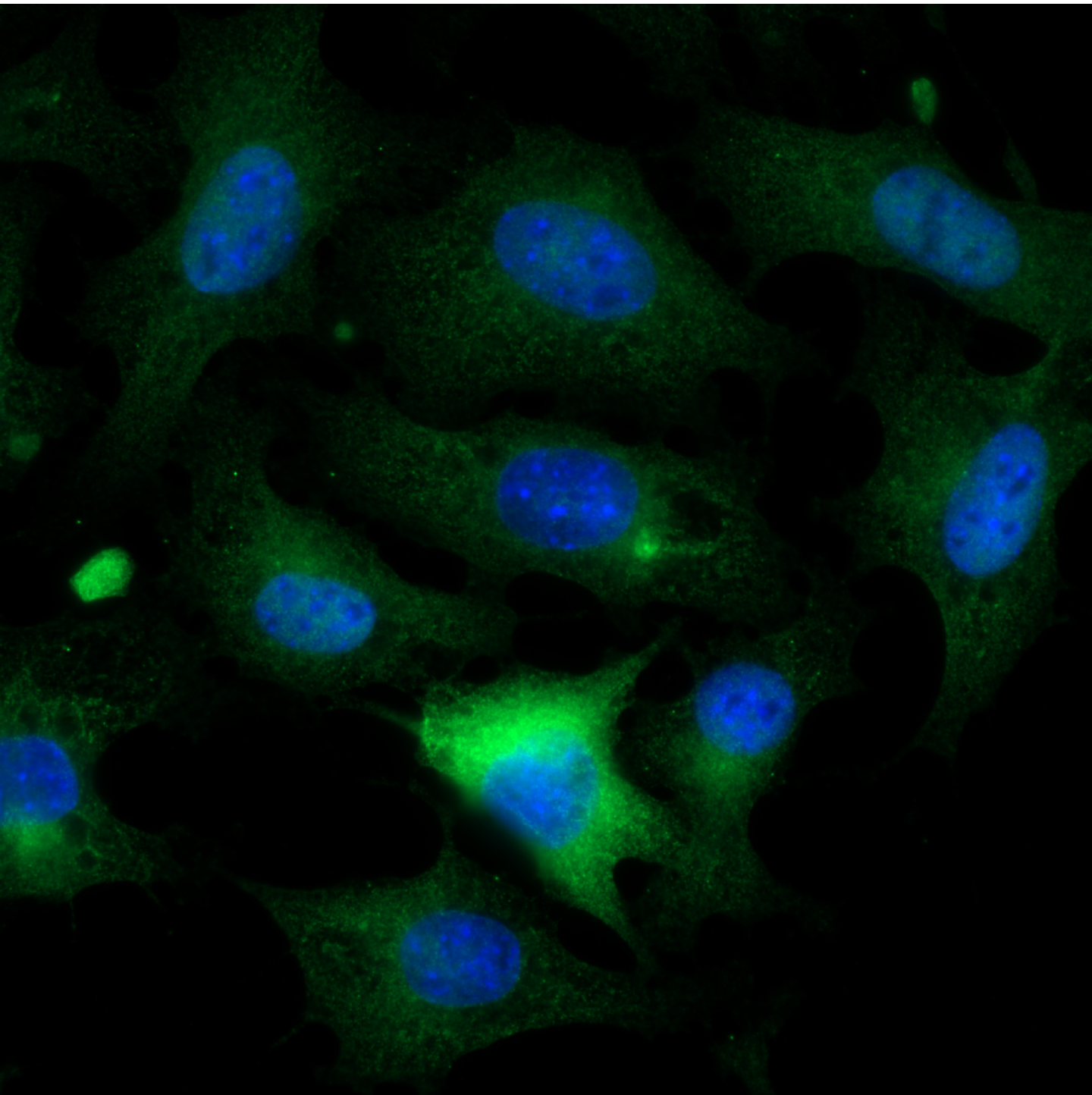

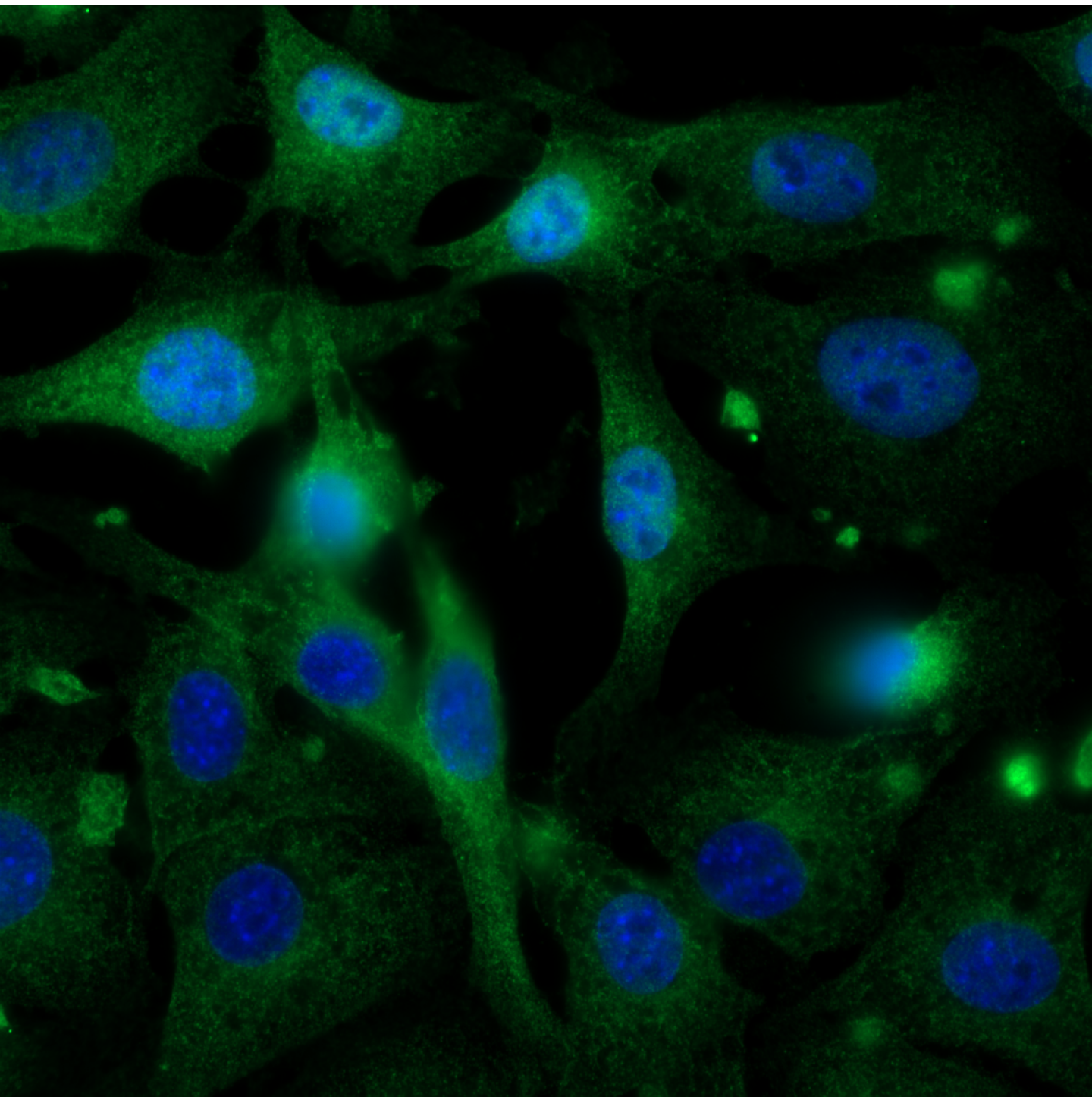

D NHA control

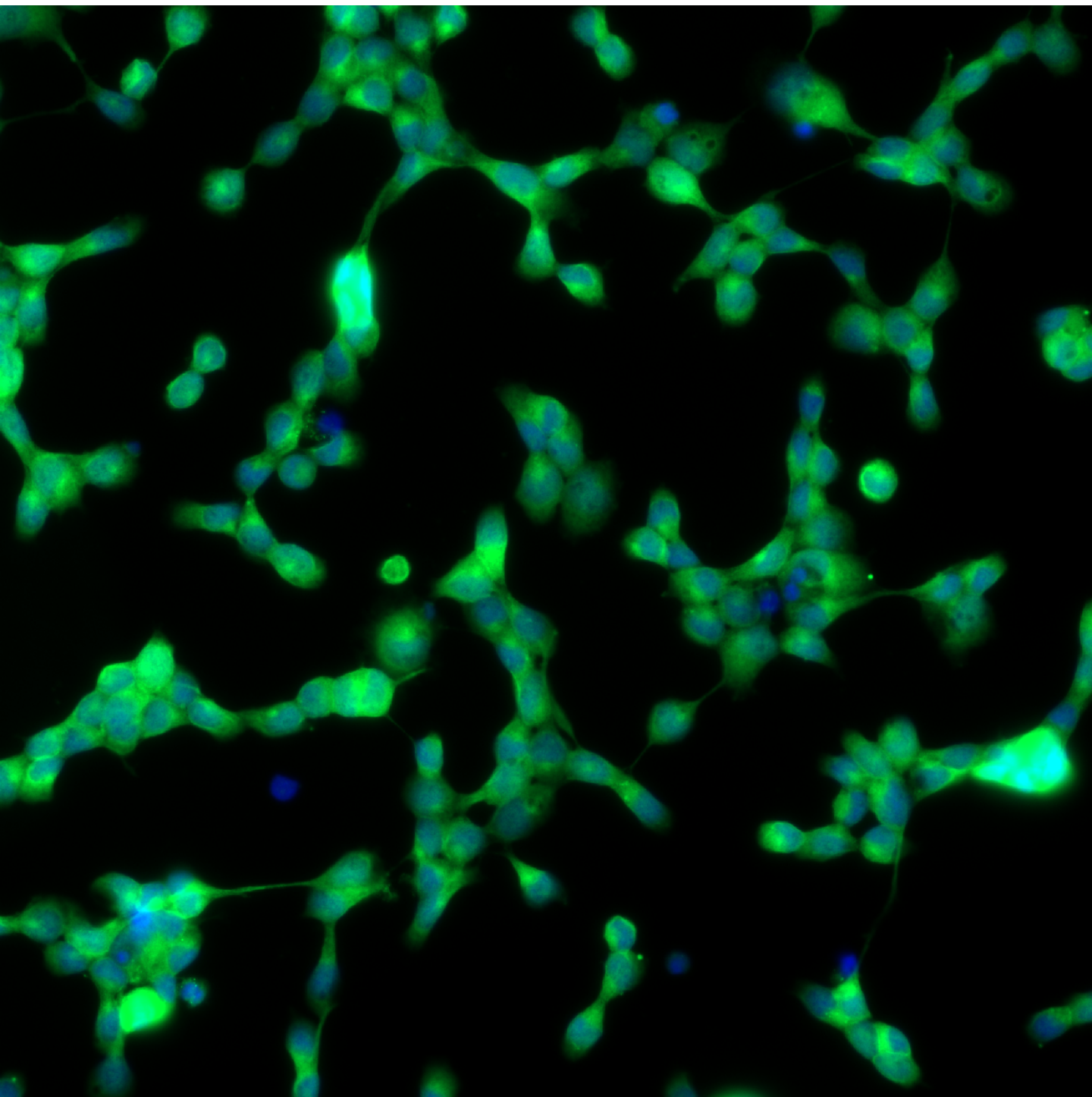

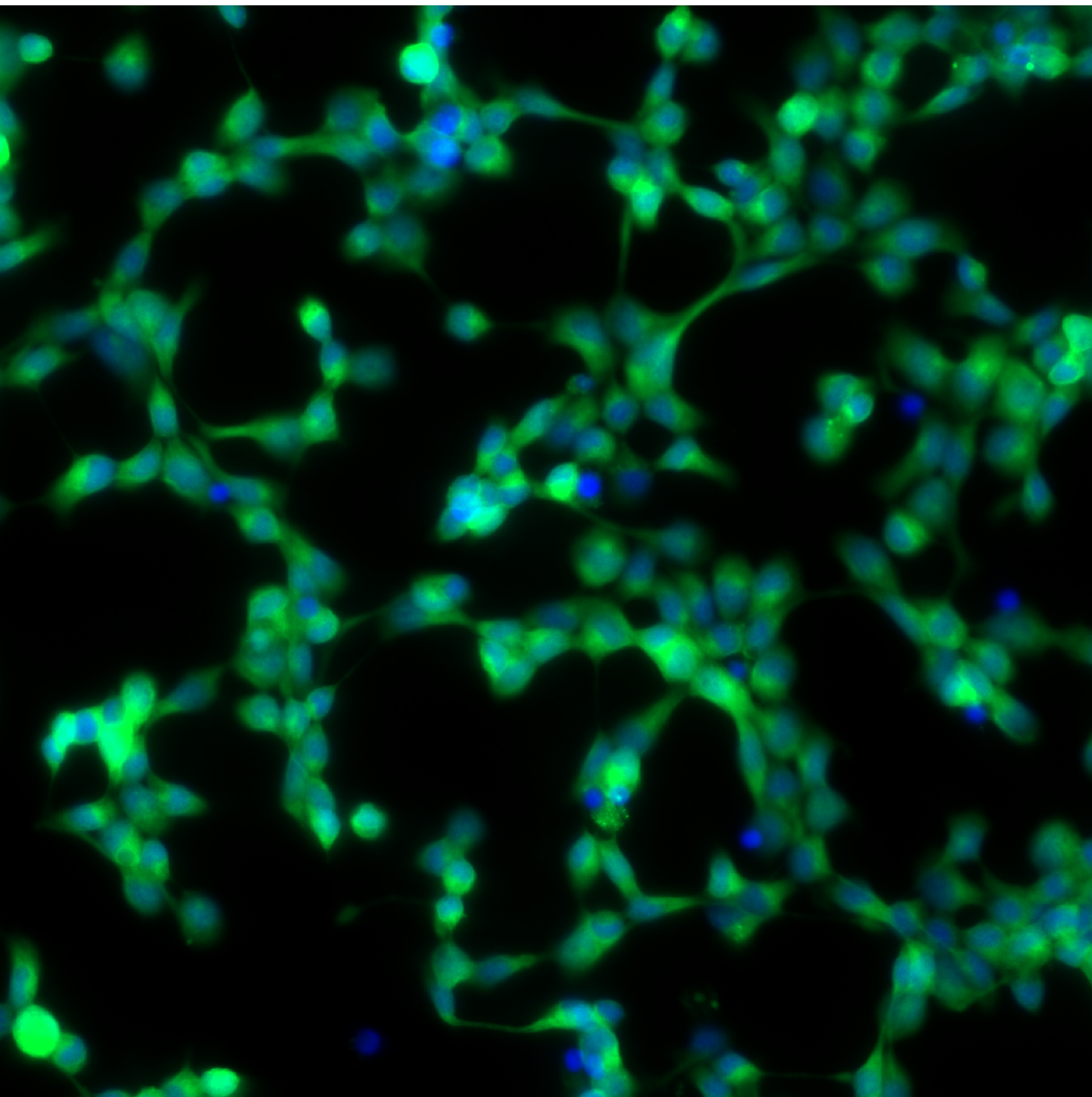

D HEK293FT control

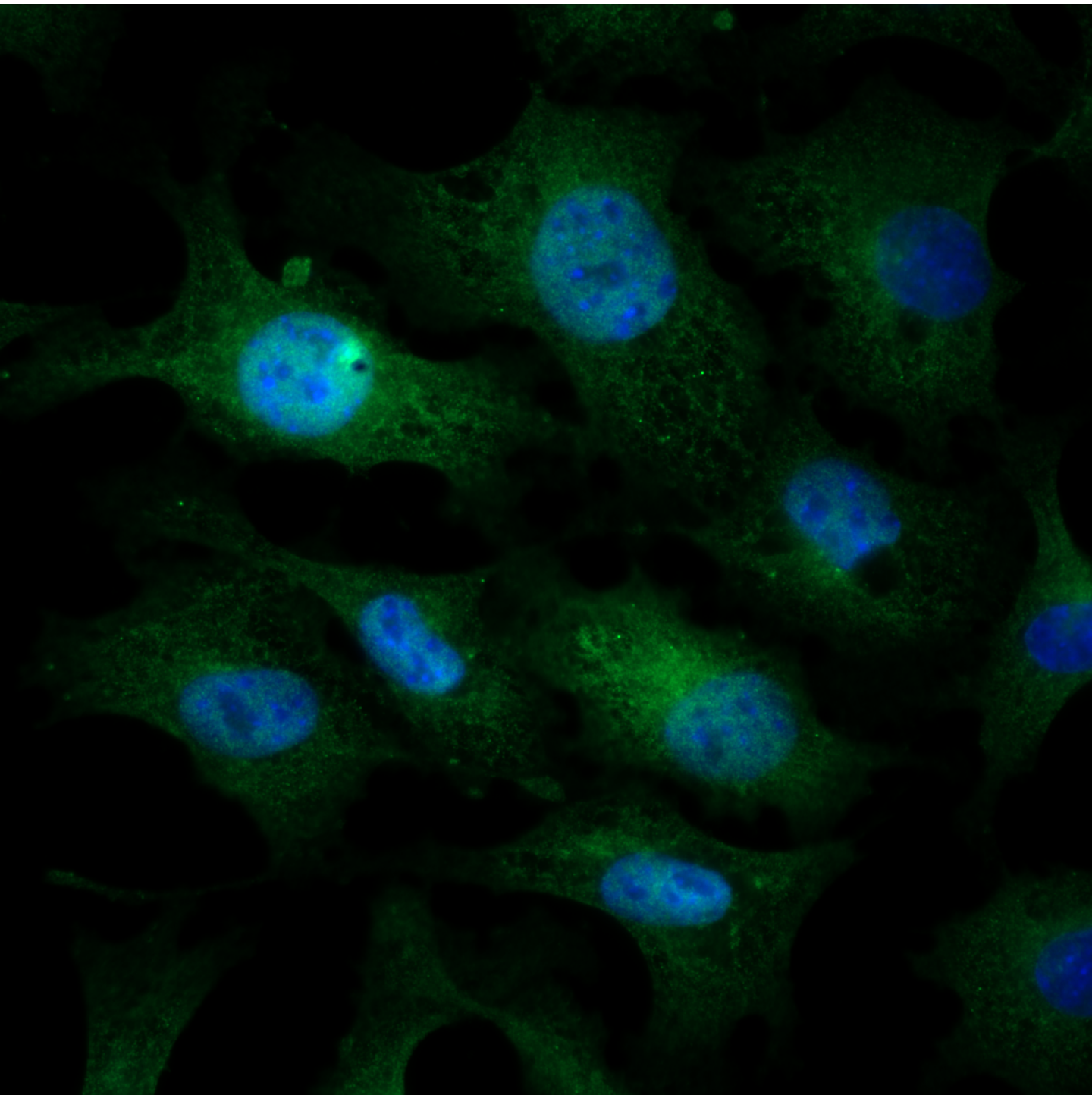

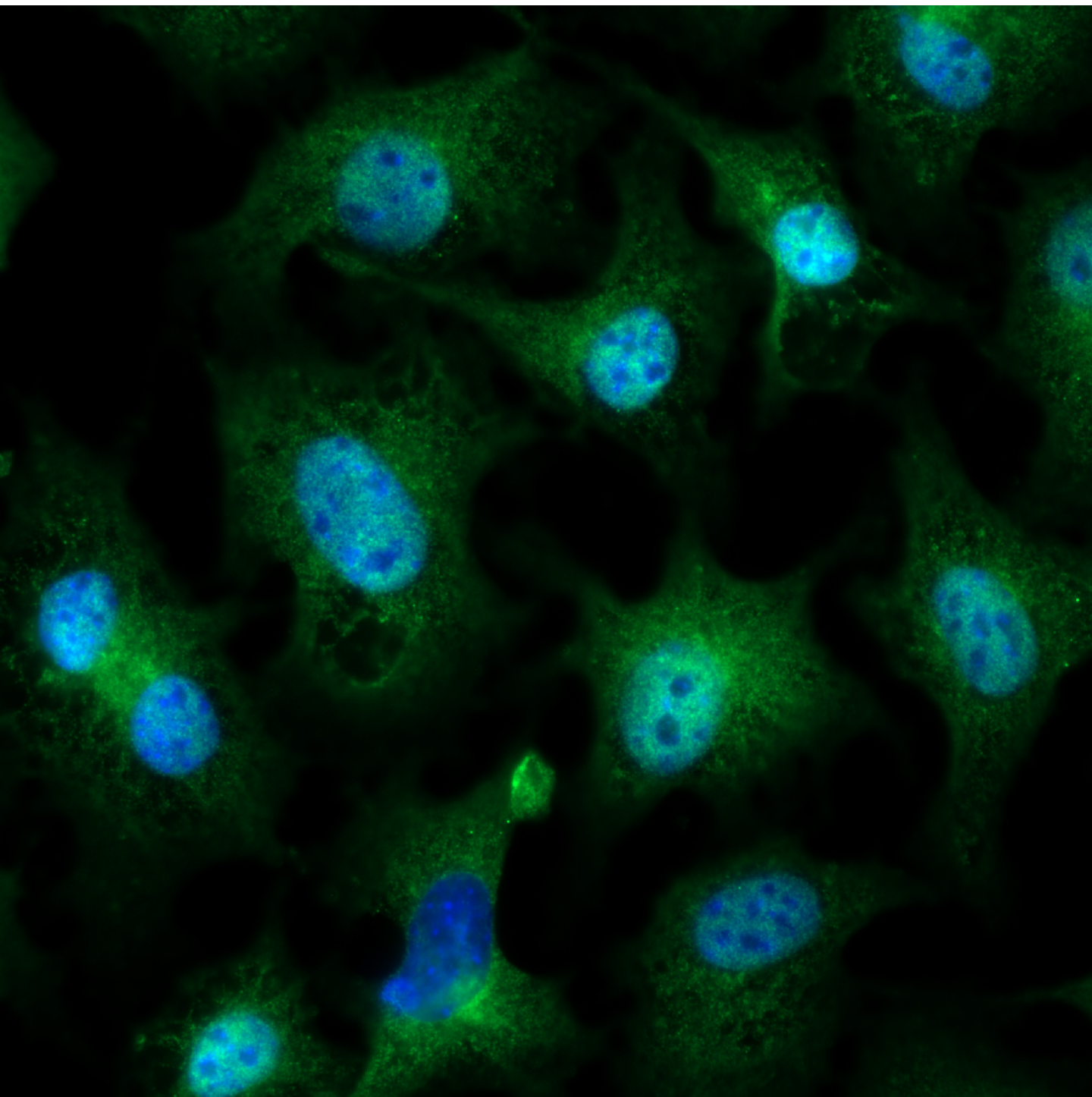

Supplement: Supplementary file 9 — Source Data for Figure 6 [file EMMM-11-e9034-s007.pdf]

A  
BT12 wt Vehicle

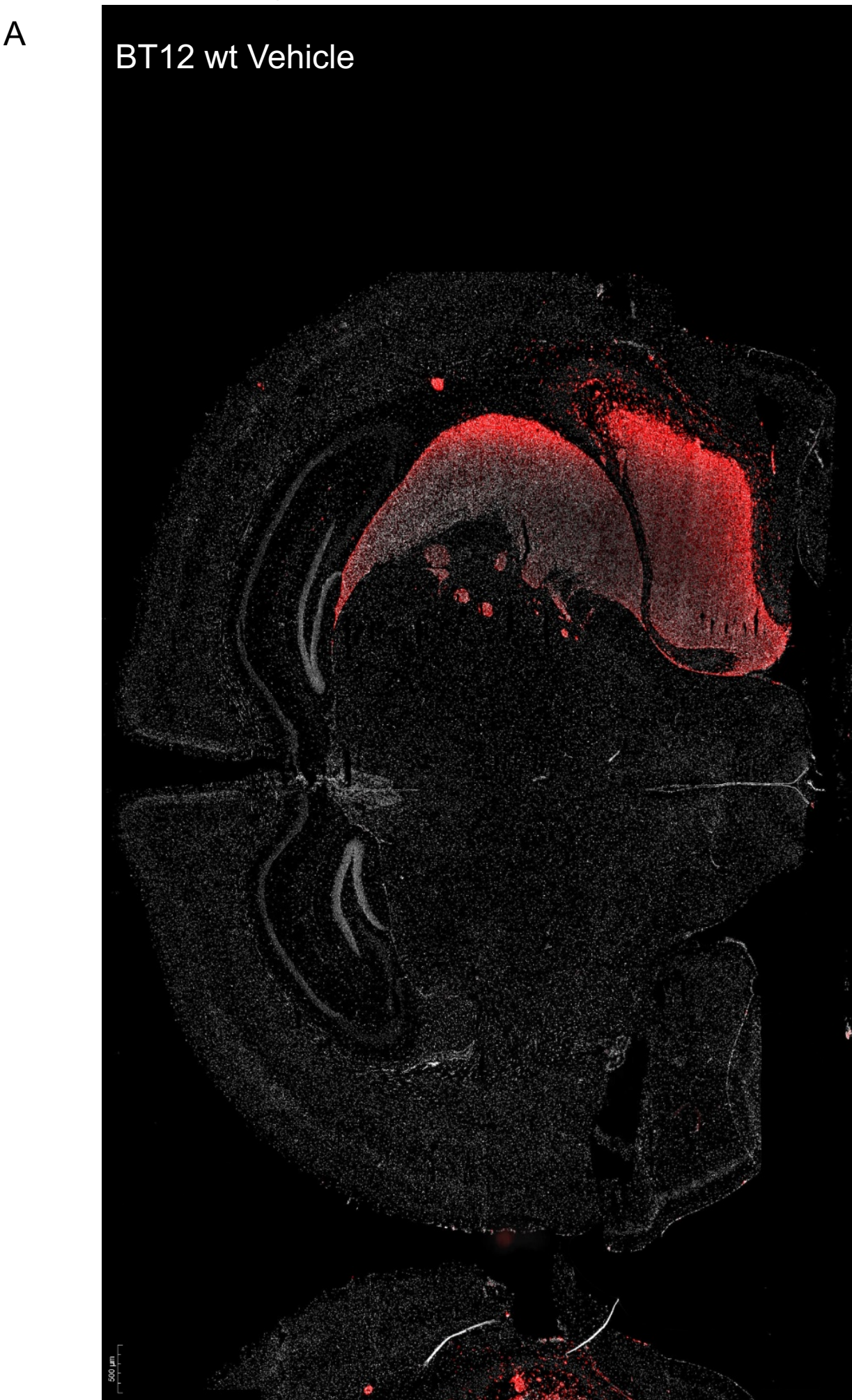

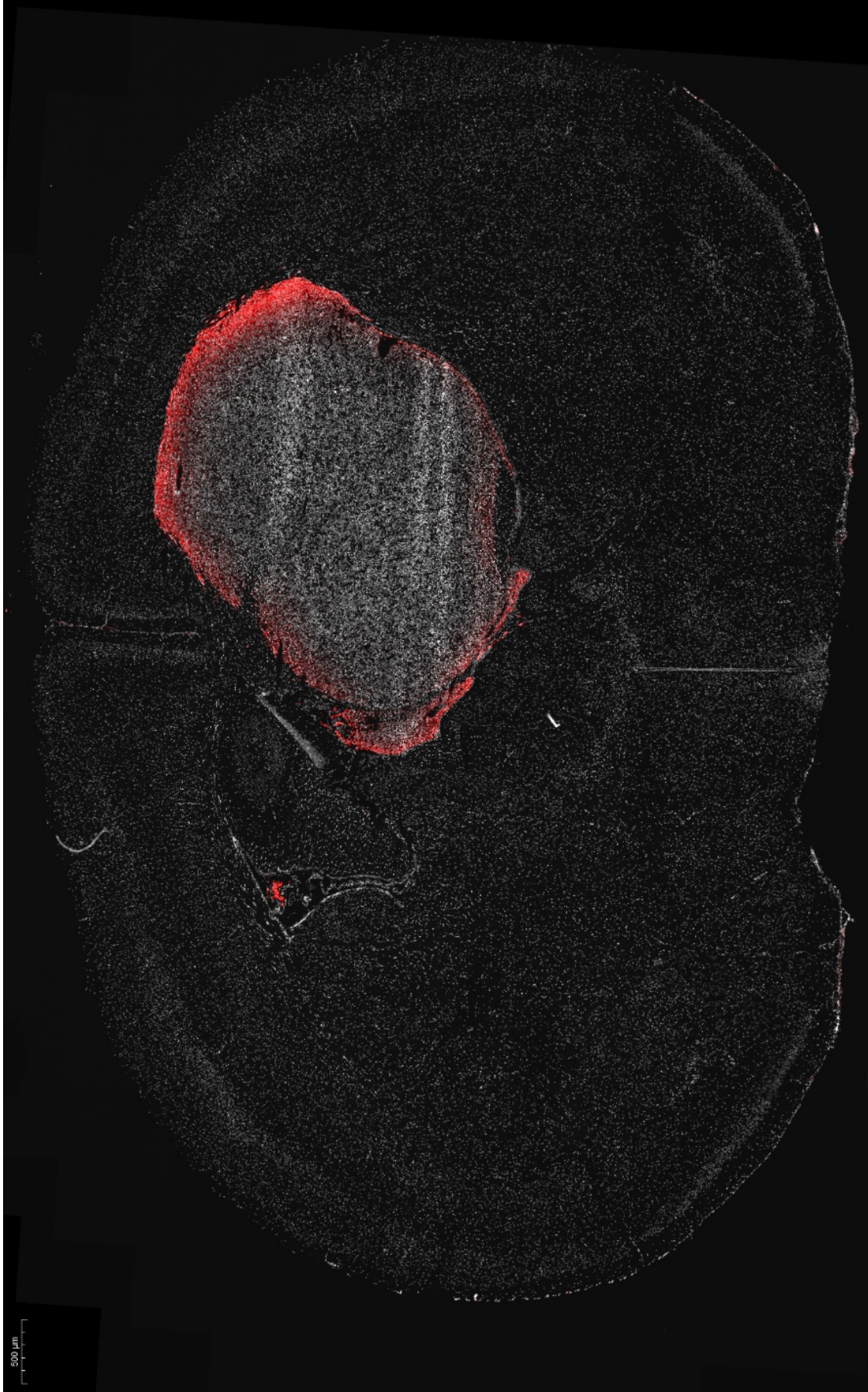

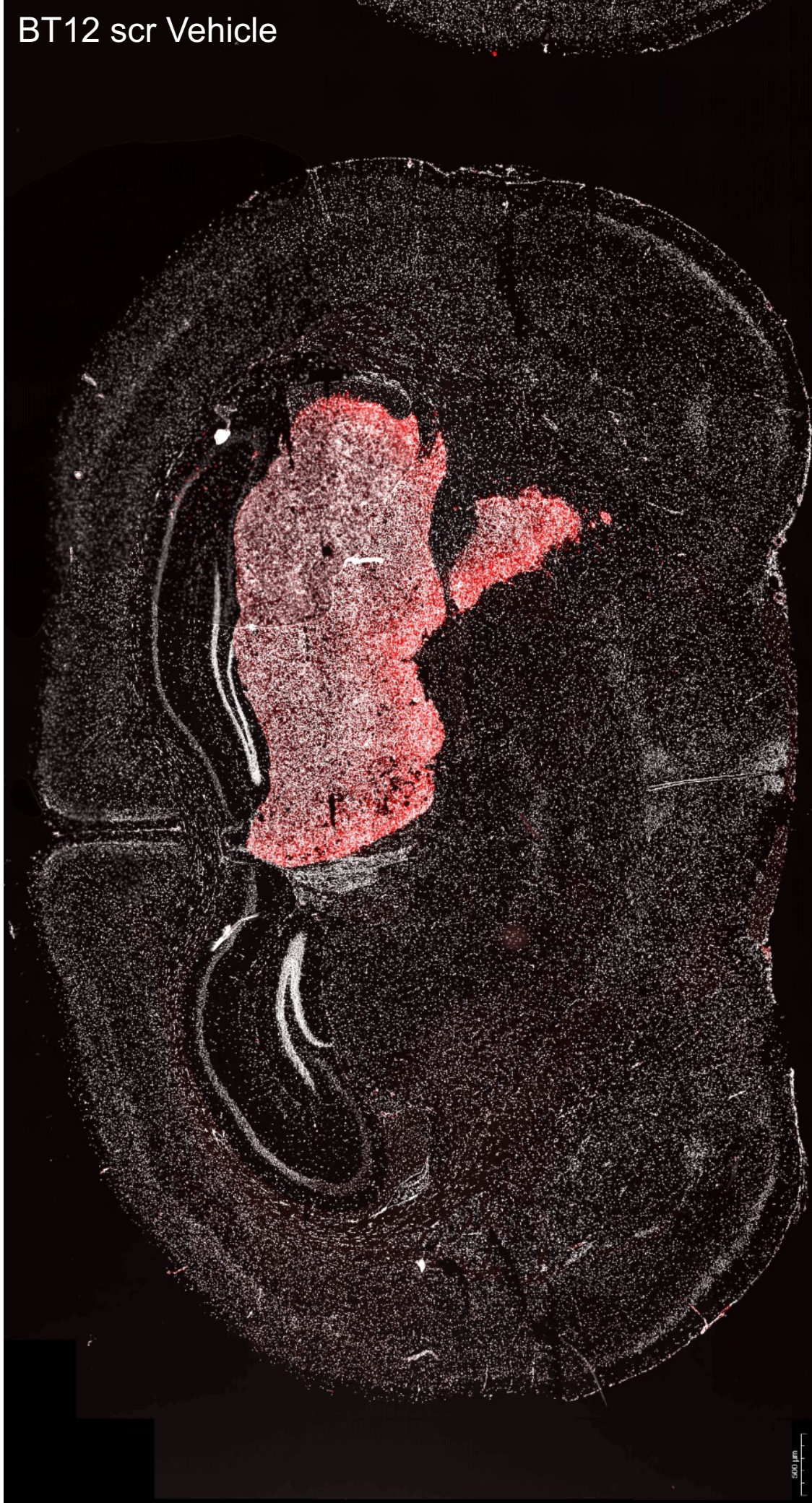

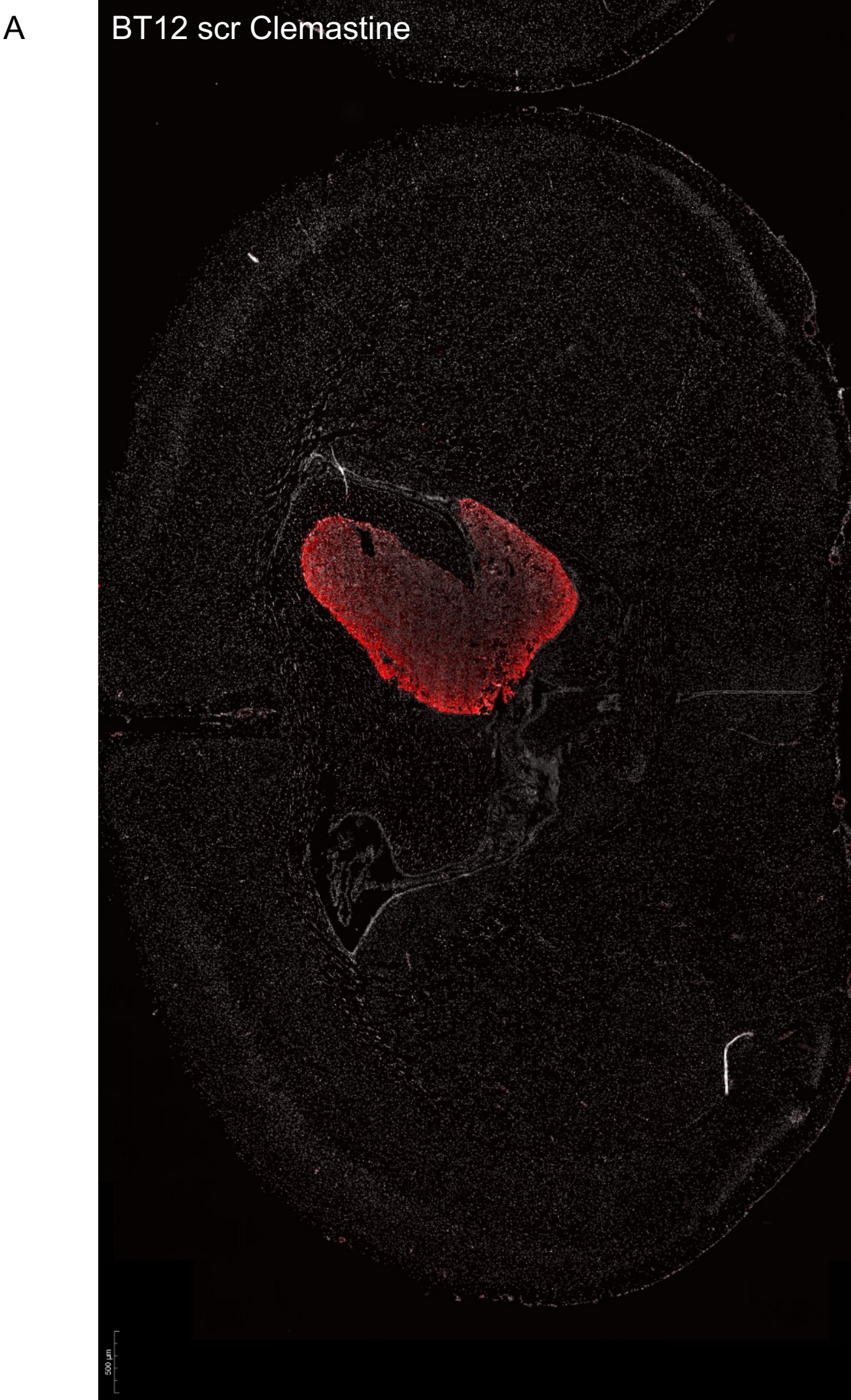

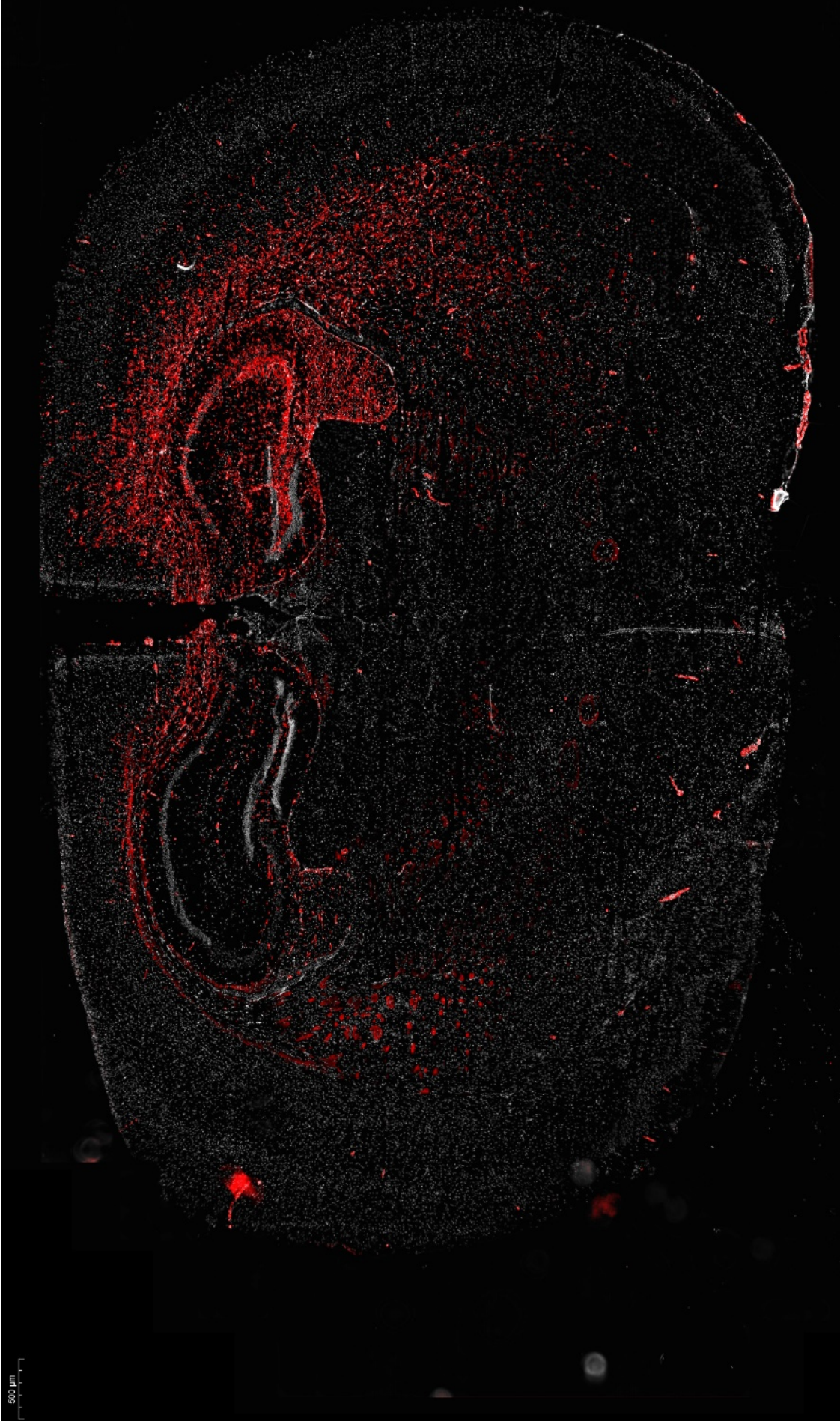

A

ZH305 Clemastine

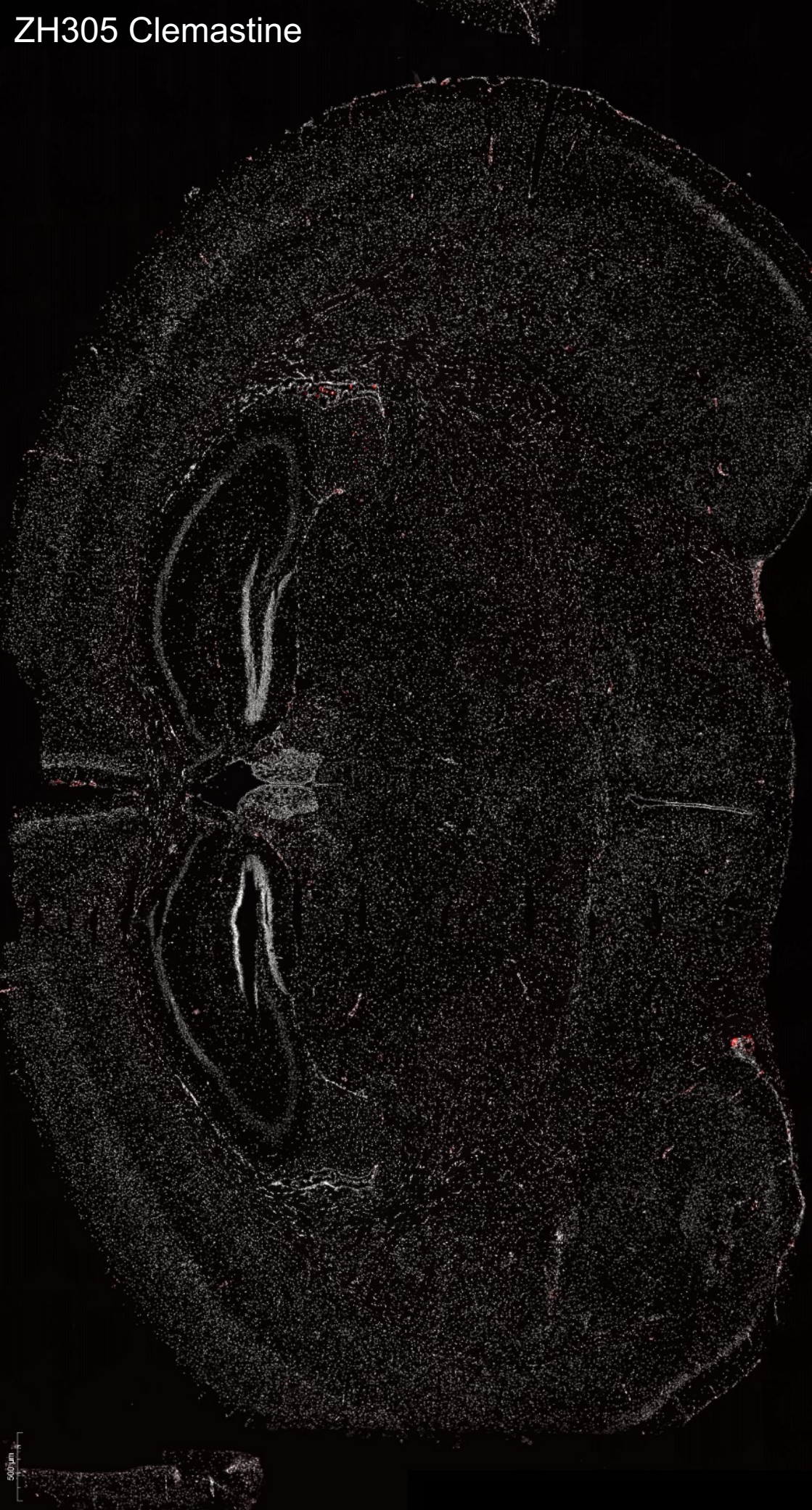

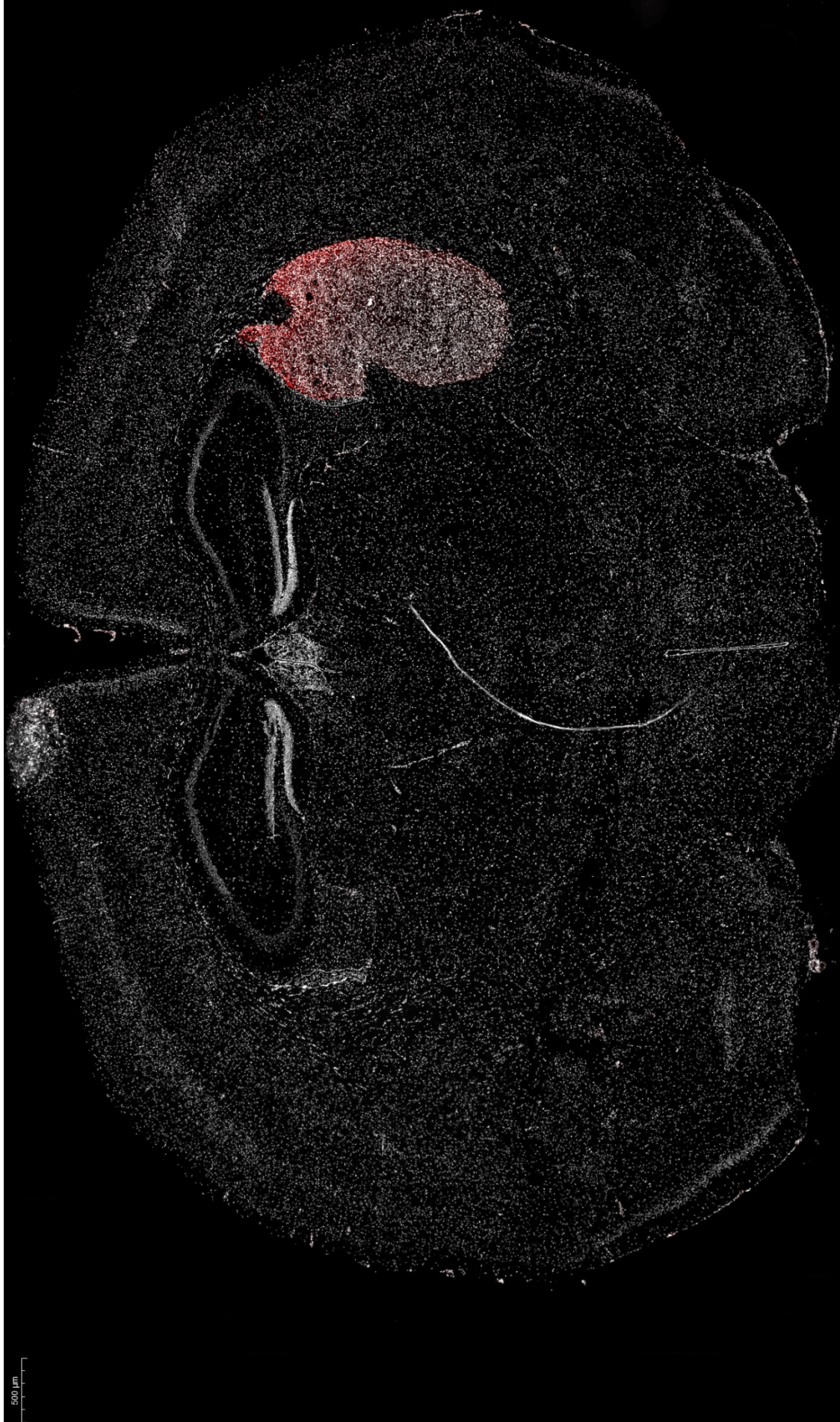

A

BT13 Clemastine

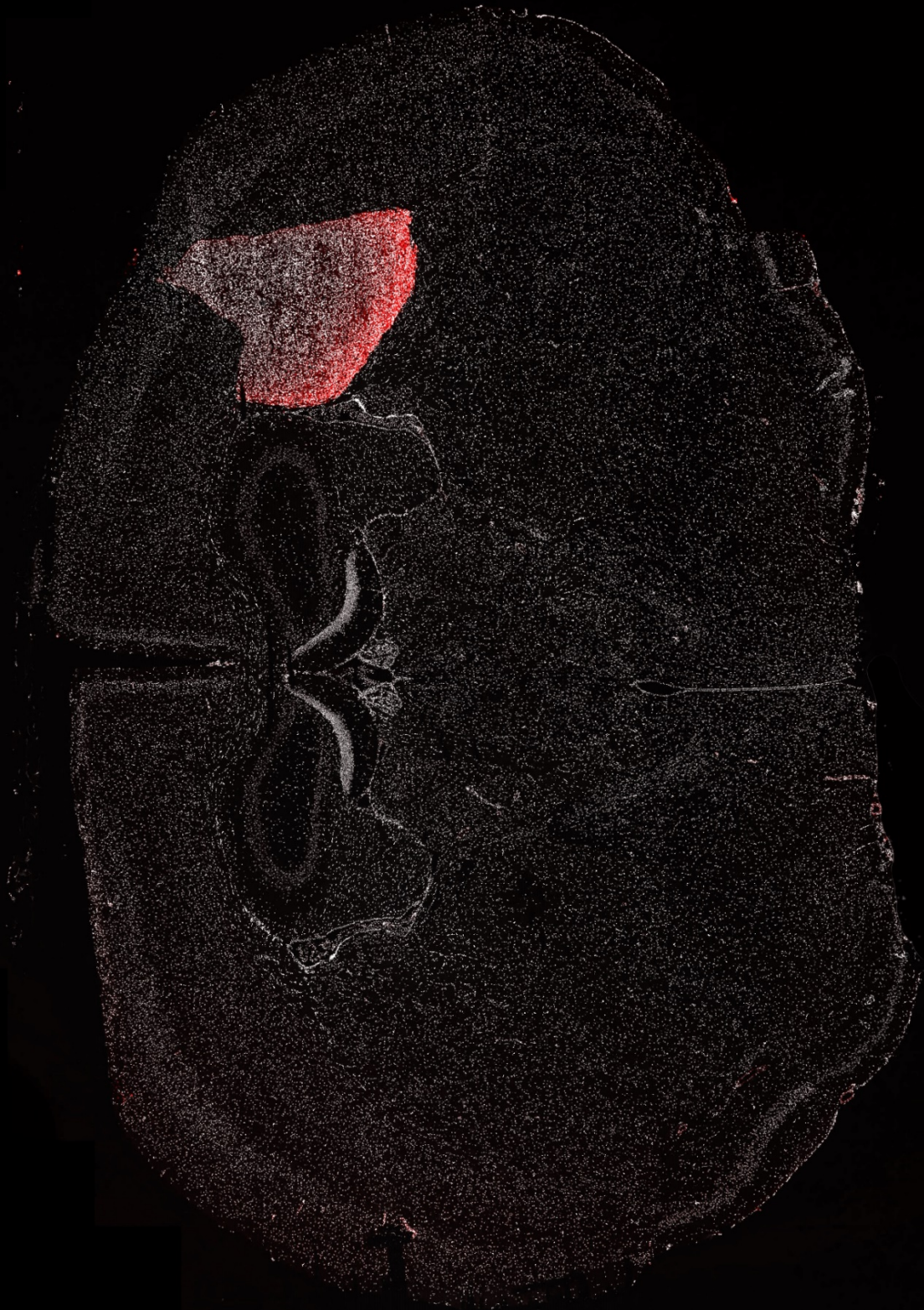

500 µm

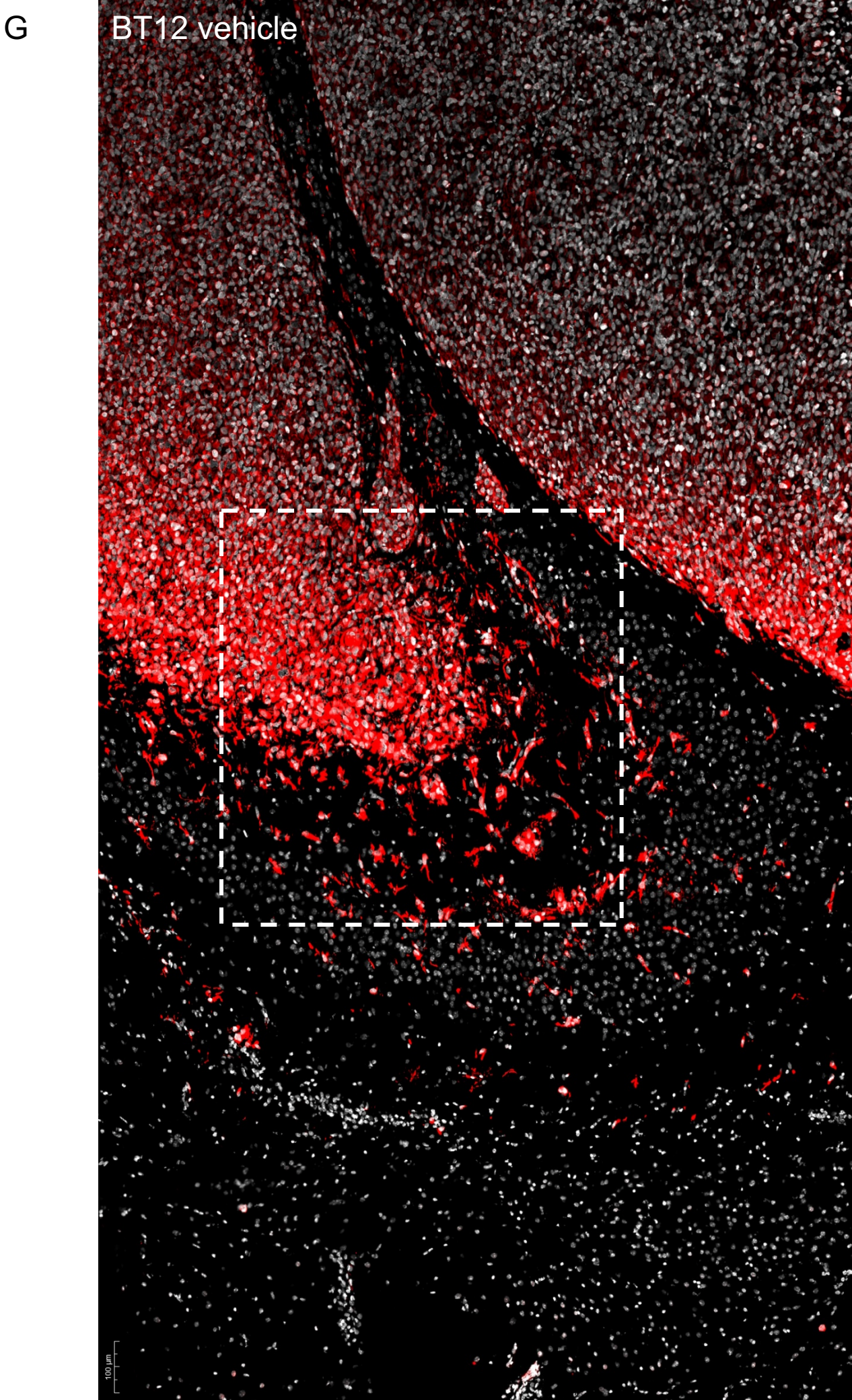

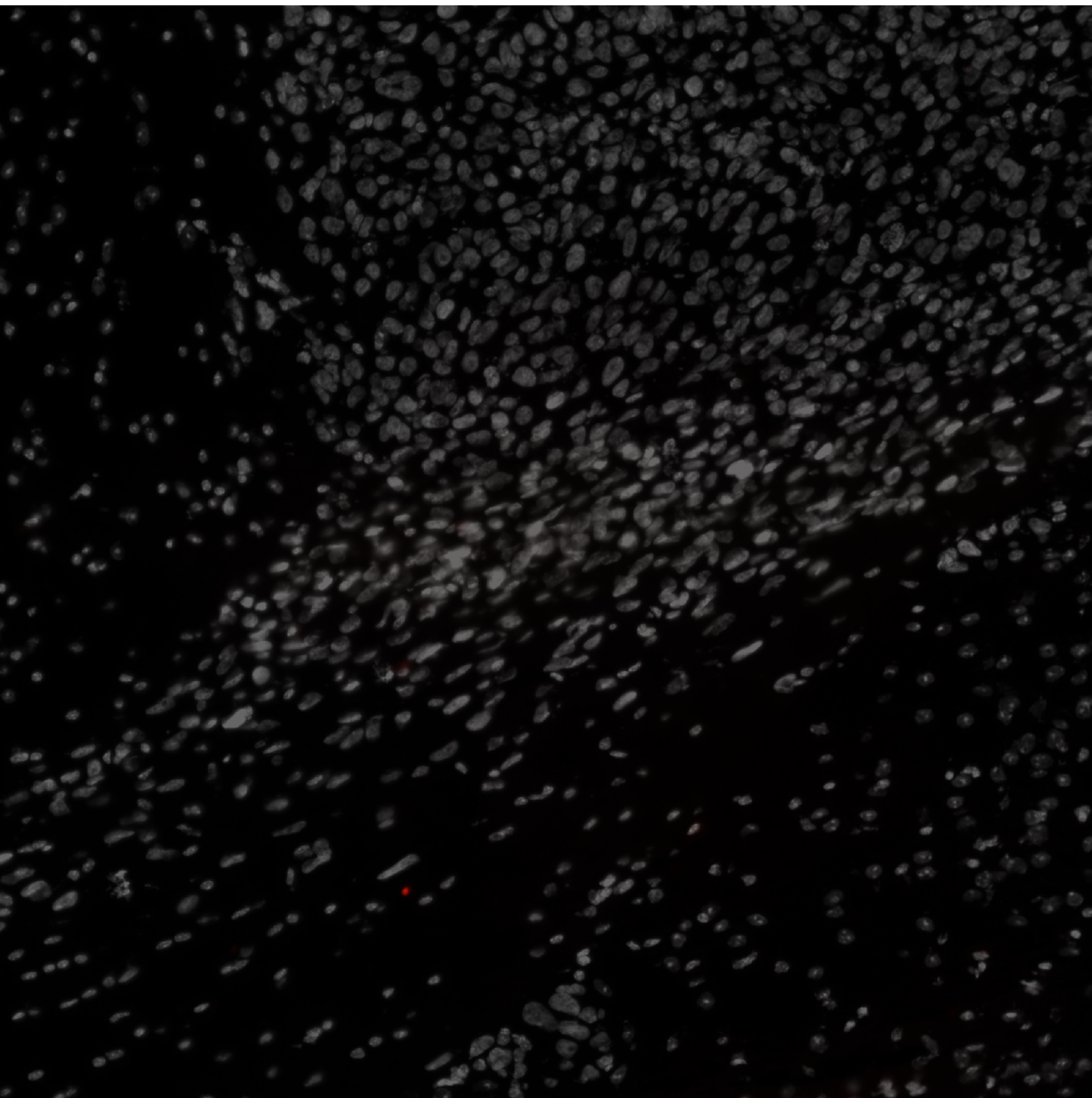

G

BT12 clemastine

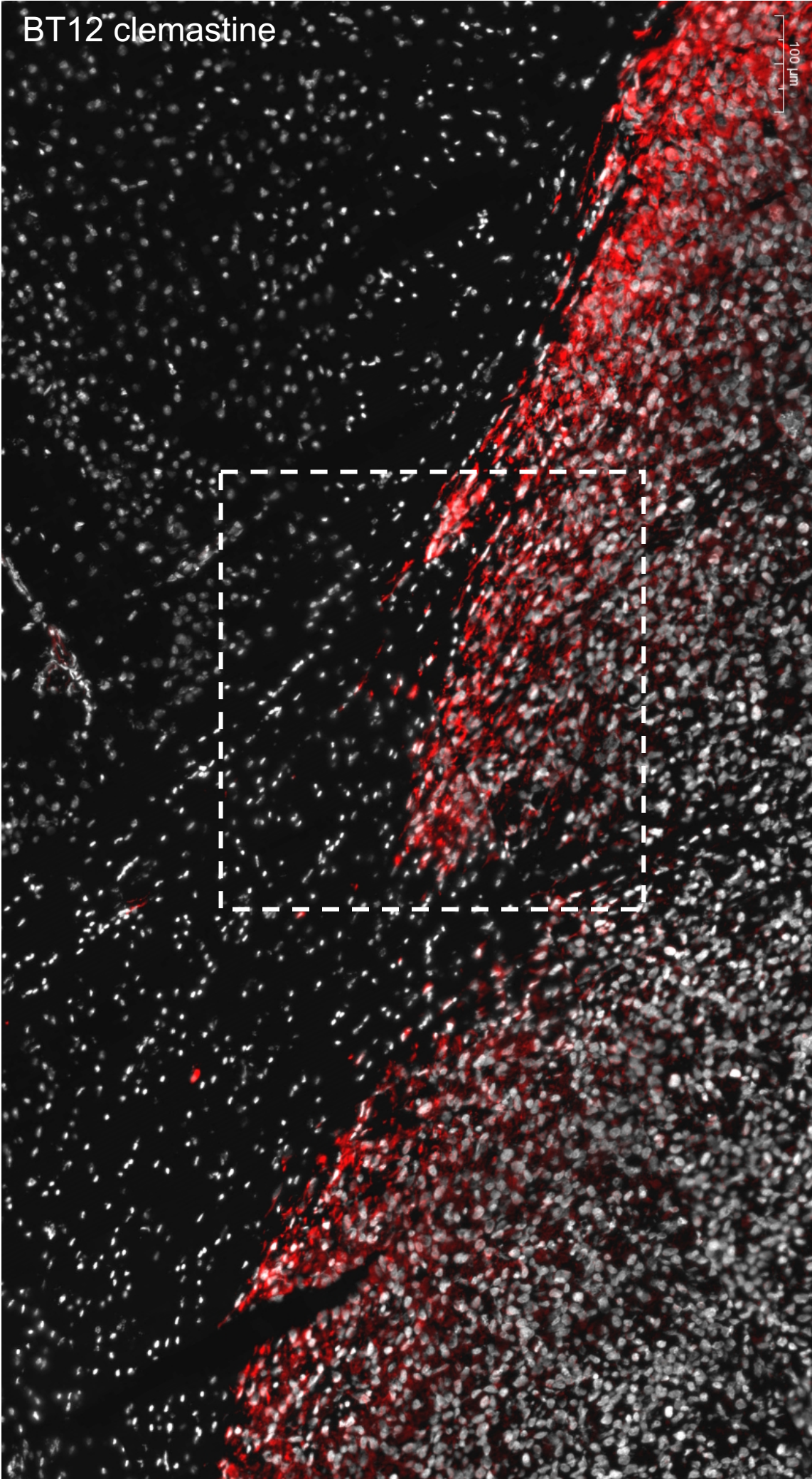

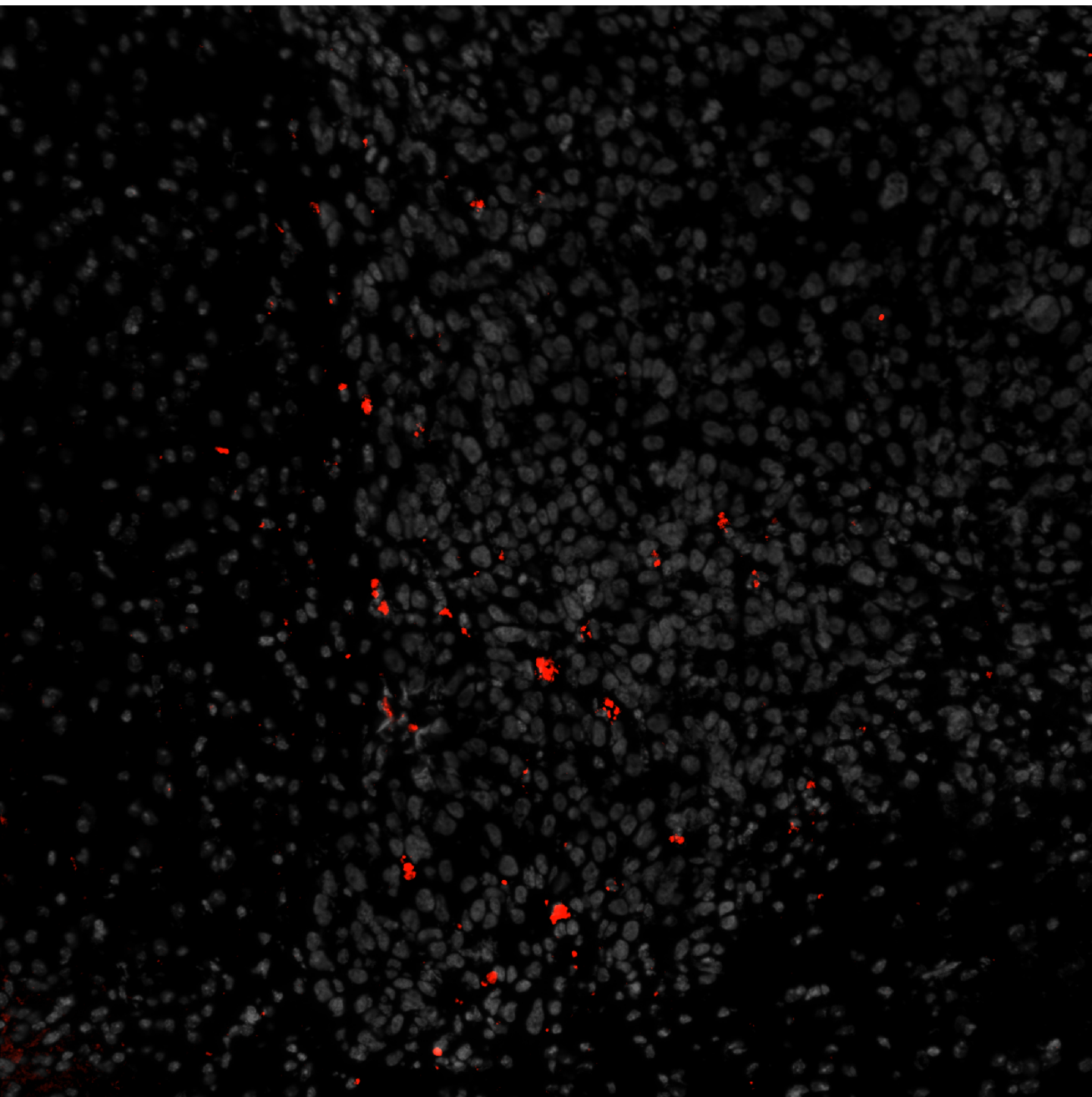

Supplement: Supplementary file 10 — Source Data for Figure 7 [file EMMM-11-e9034-s008.pdf]
