# Supplementary material for: Vulnerability of invasive glioblastoma cells to lysosomal membrane destabilization
Source: EMBO Mol Med. 2019 May 8;11(6):e9034. doi: 10.15252/emmm.201809034 (PMC6554674; doi:10.15252/emmm.201809034)

Source data for Fig 3

A

BT12 Scr

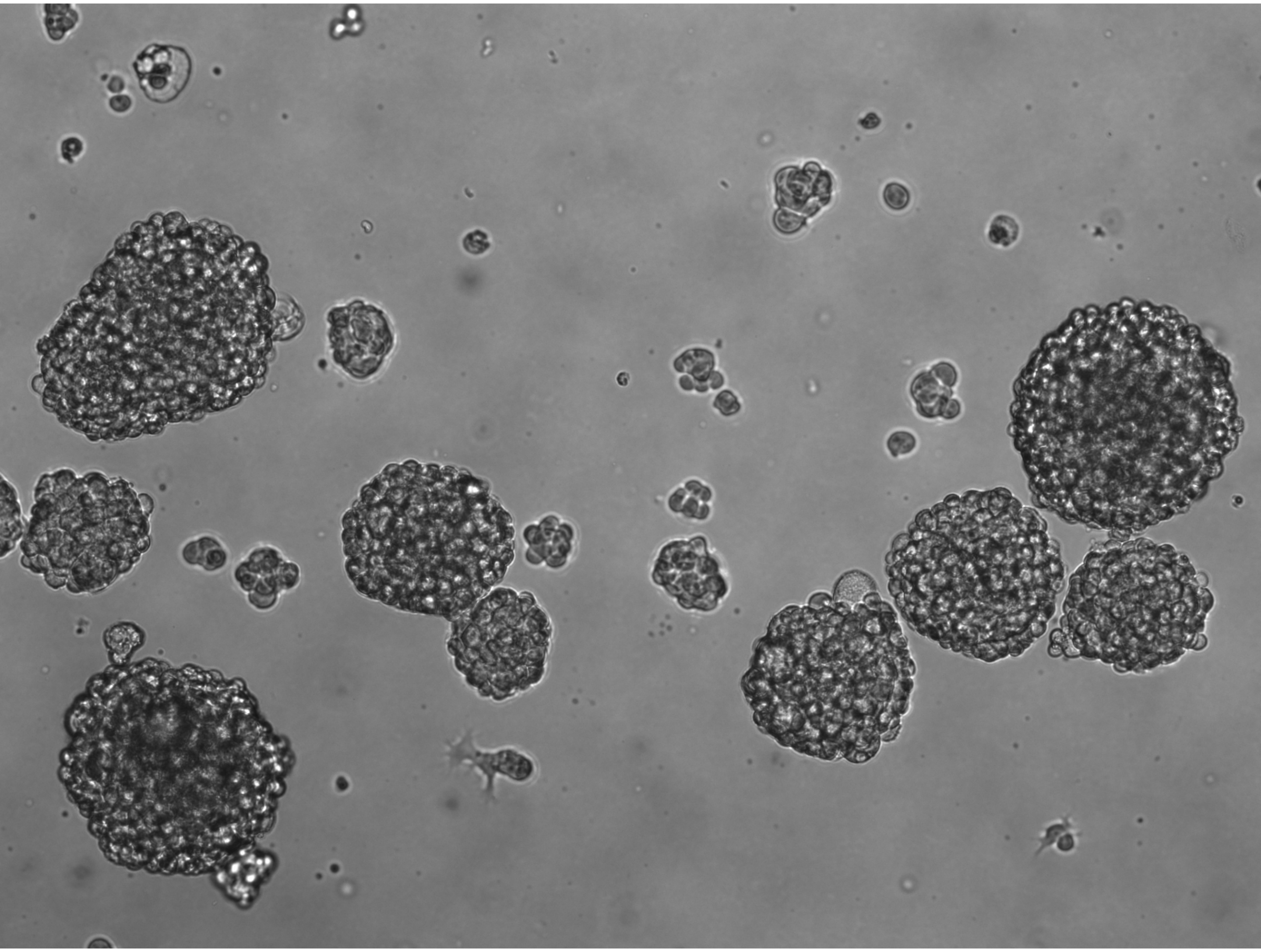

A

BT12 shMDGI1

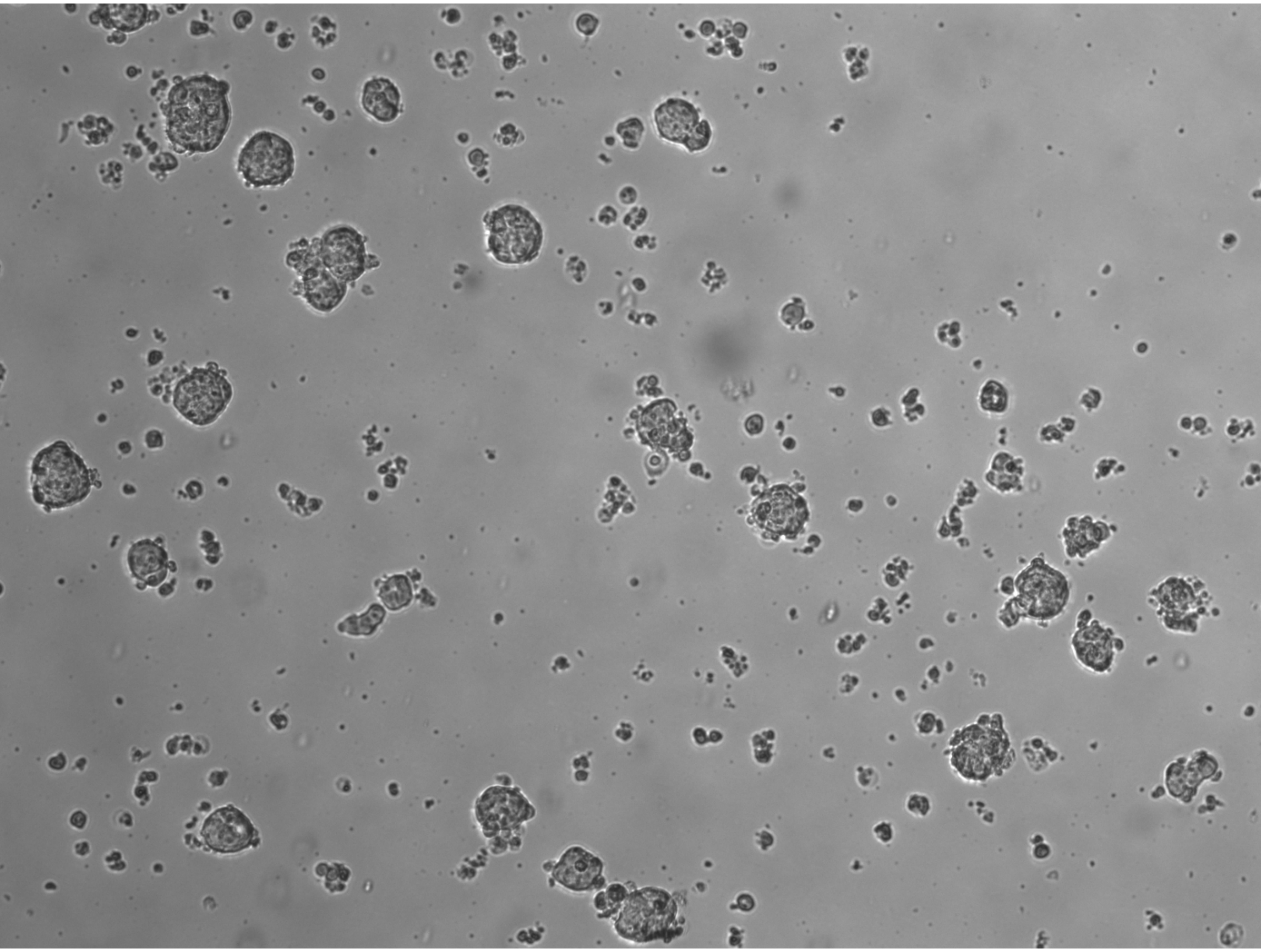

A

BT12 shMDGI2

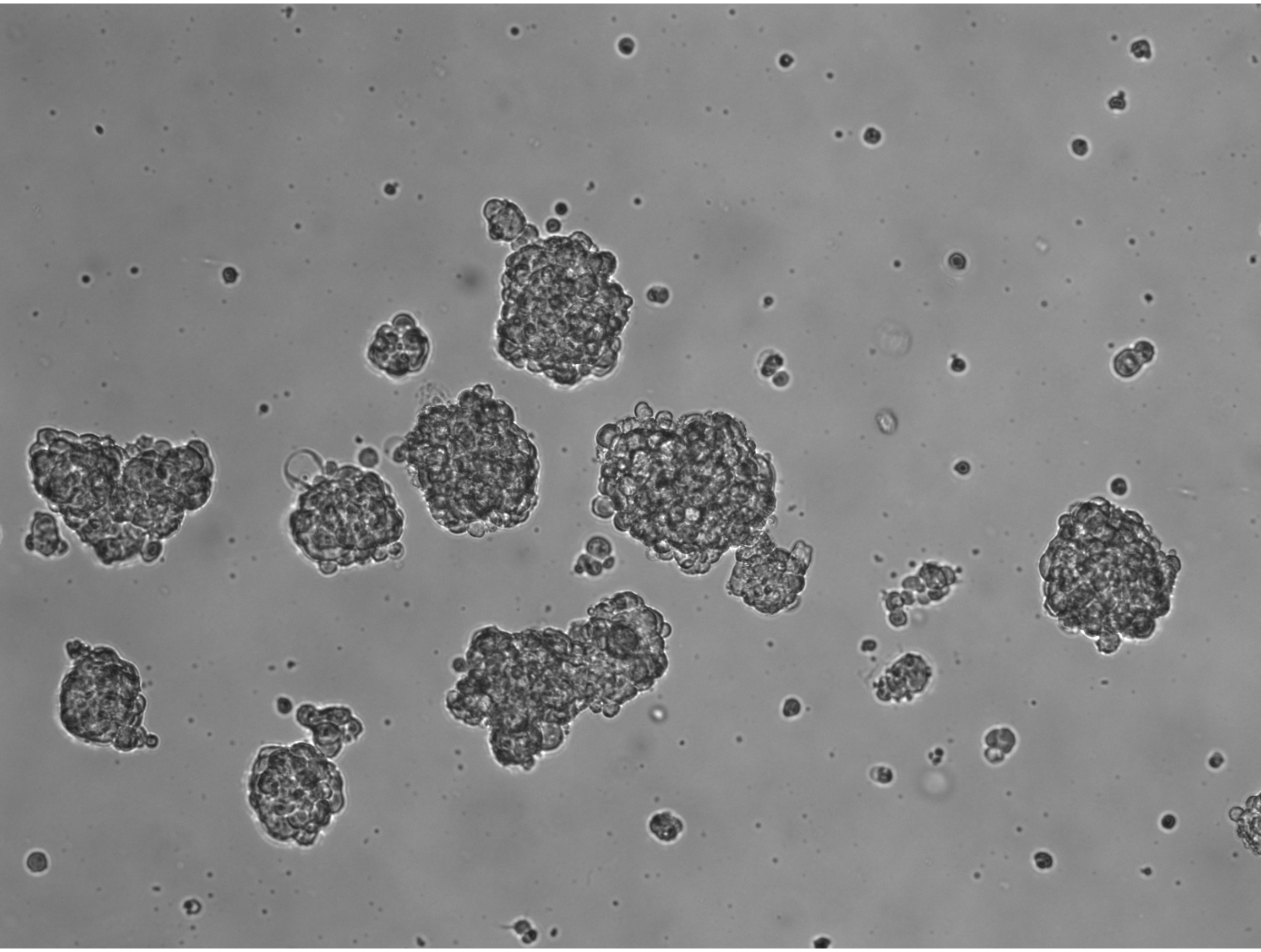

A

BT13 Scr

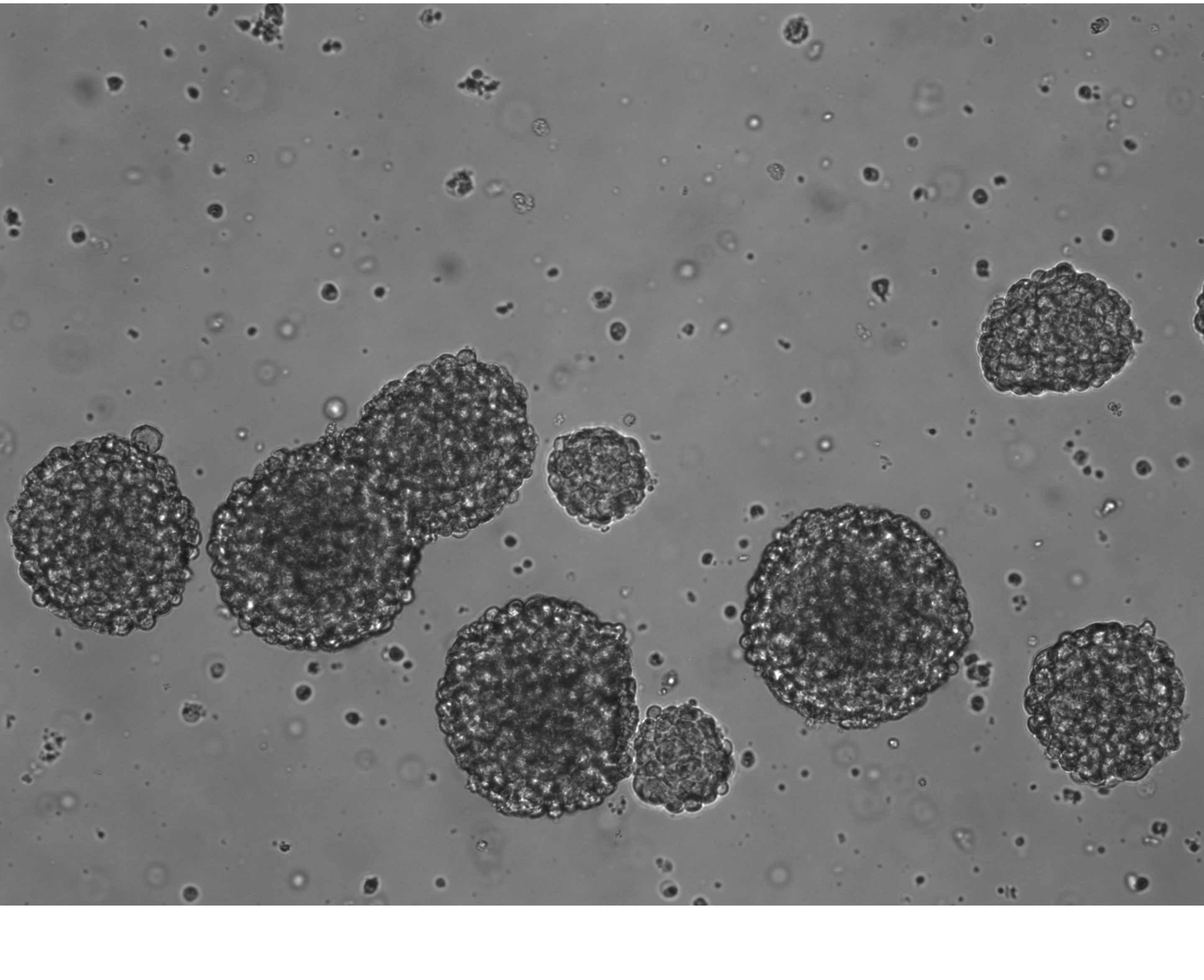

A

BT13 shMDGI1

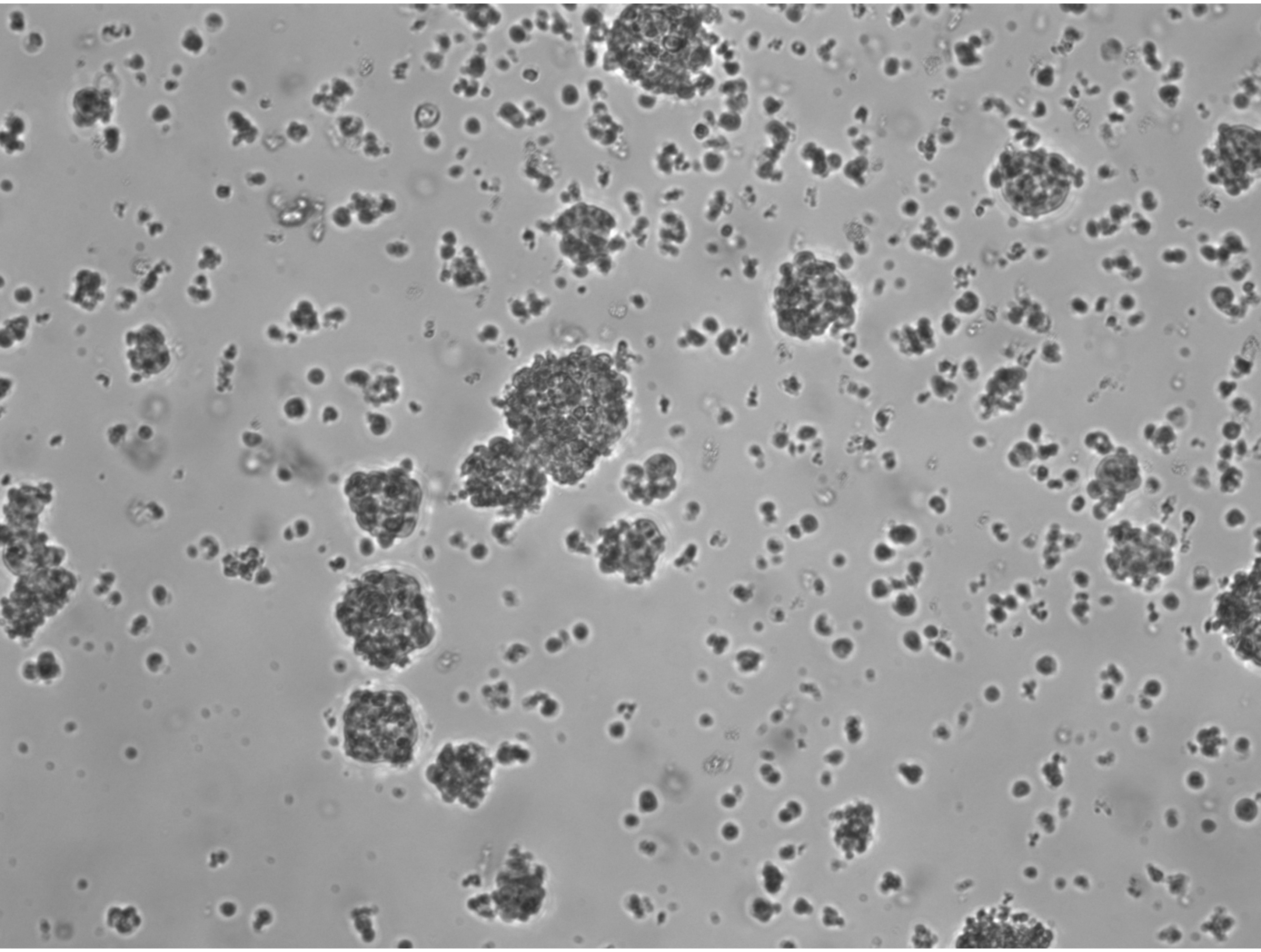

A

BT13 shMDGI2

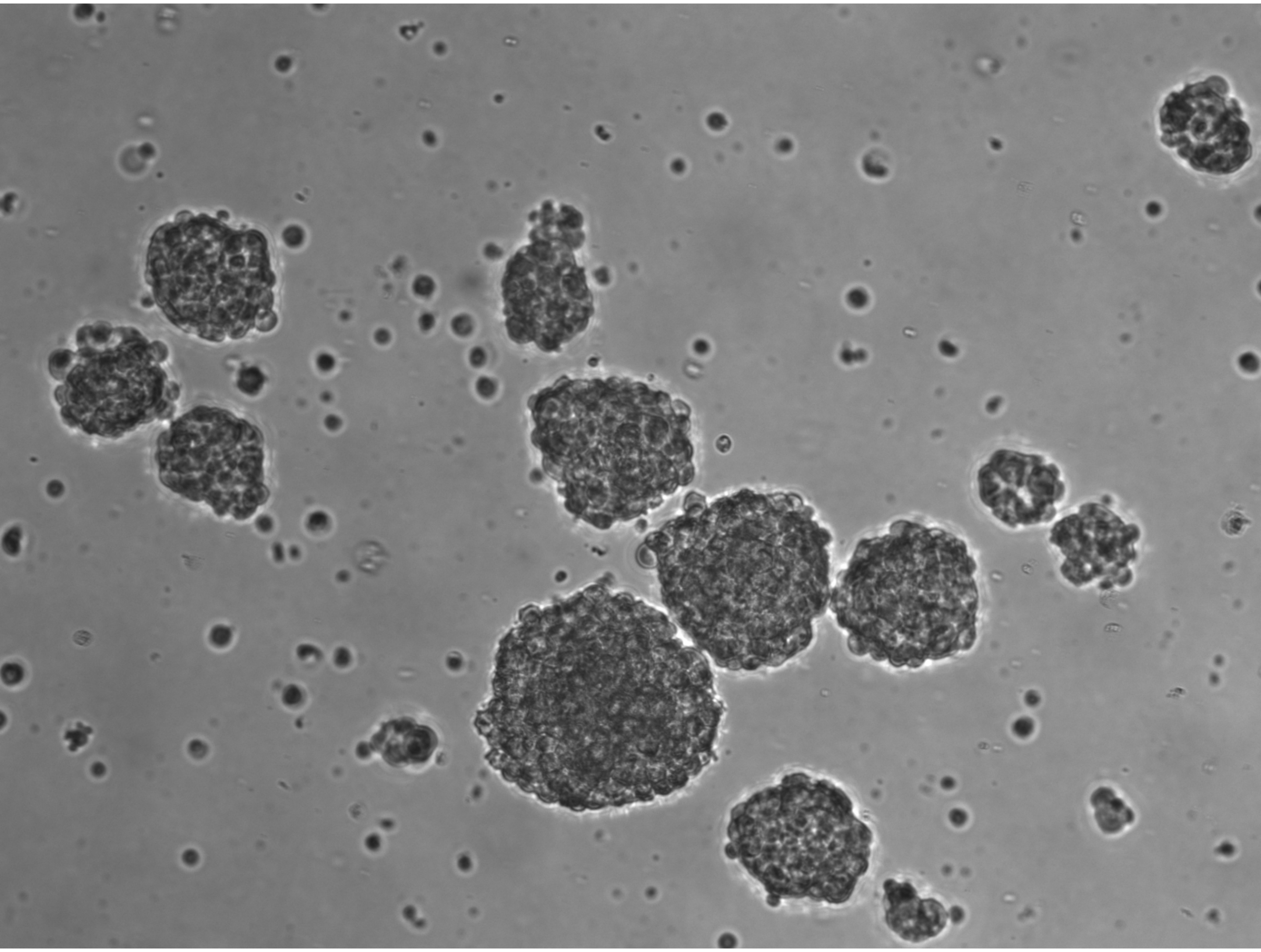

B

Representative 17/17 20x tiled fields quantified with CellProfiler. Dotted rectangle indicates the cropped illustration on Figure 3.

BT12 scr

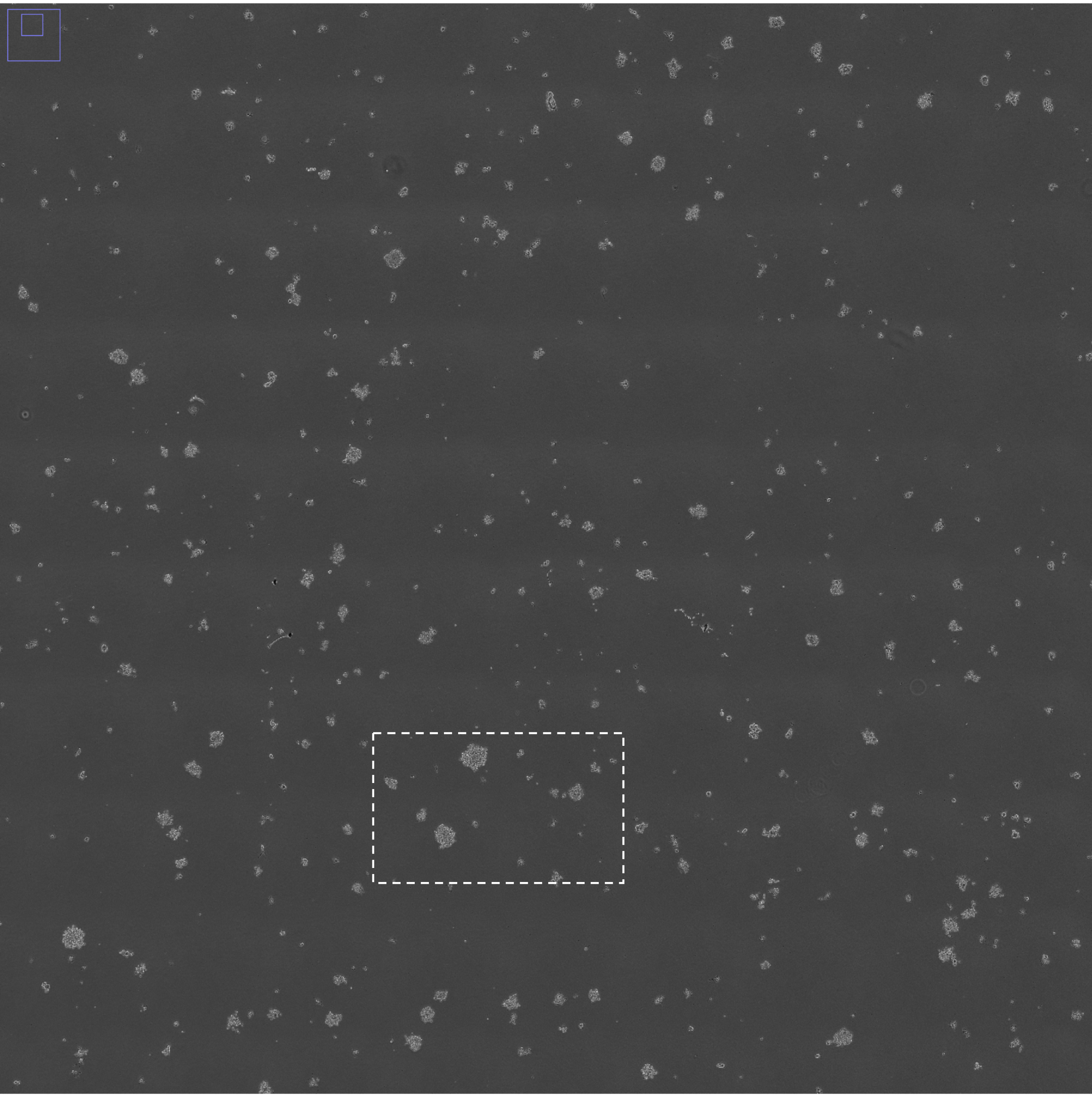

B

BT12 shMDGI1

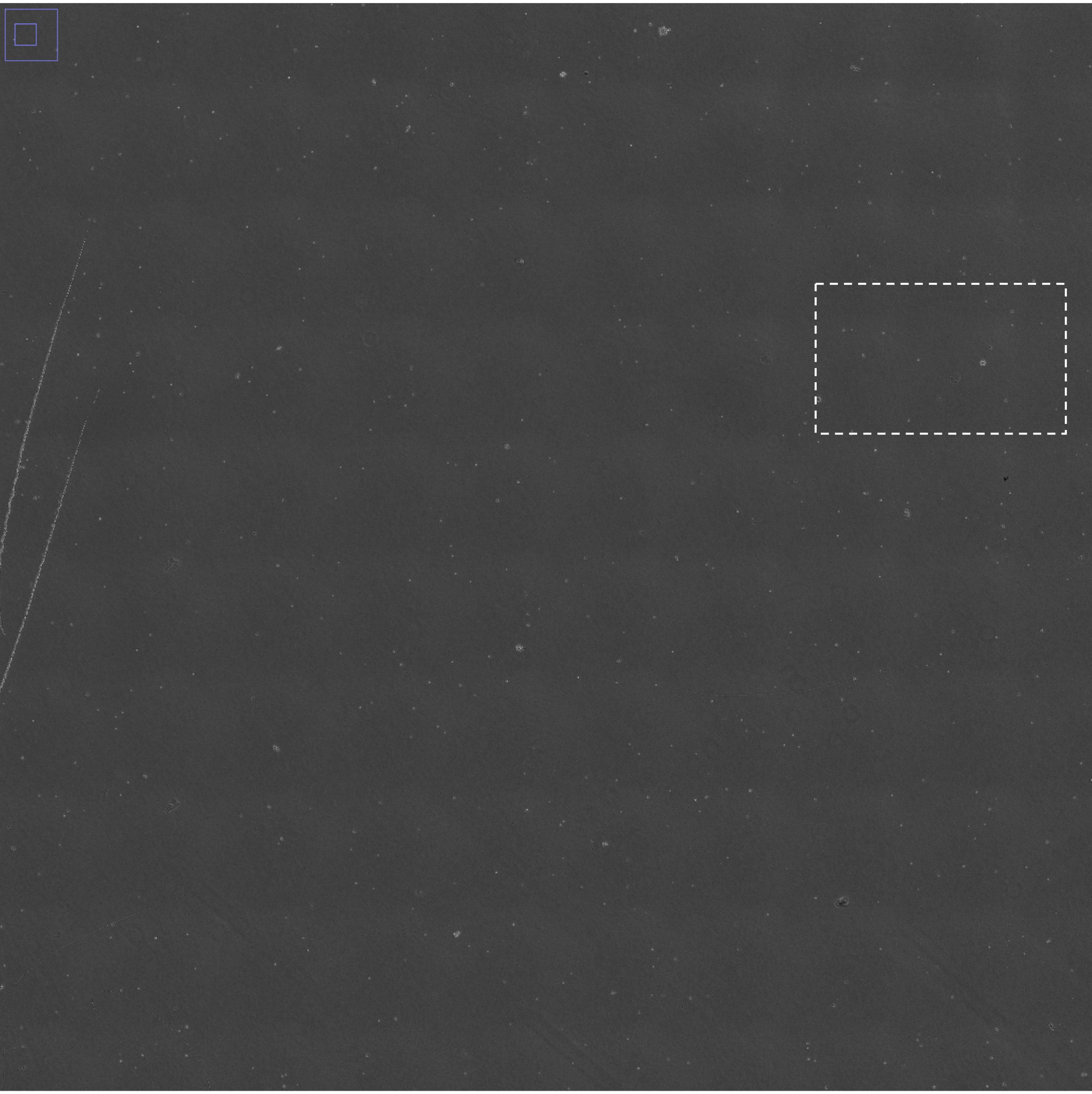

B

BT12 shMDGI2

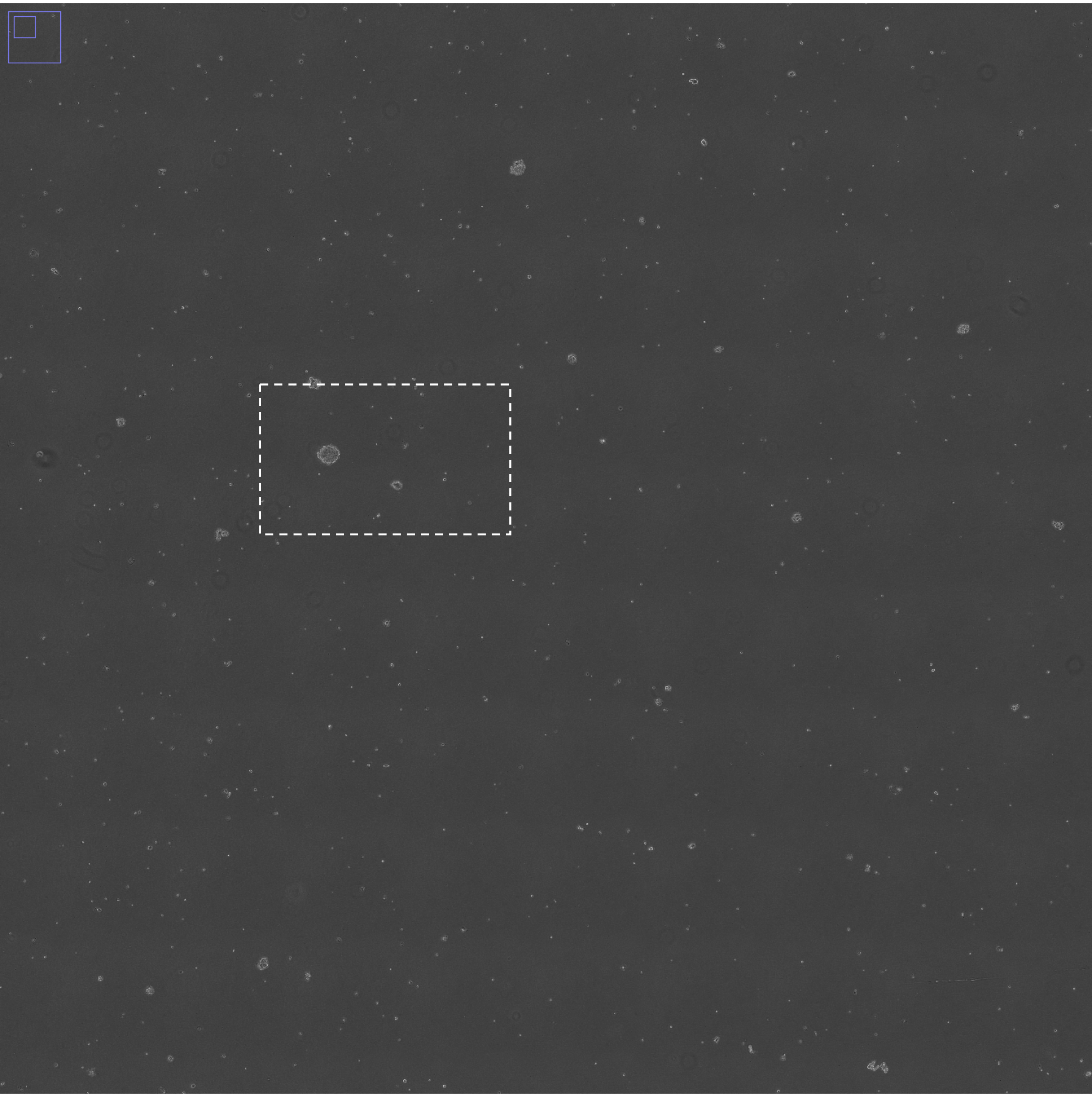

B

BT13 Scr

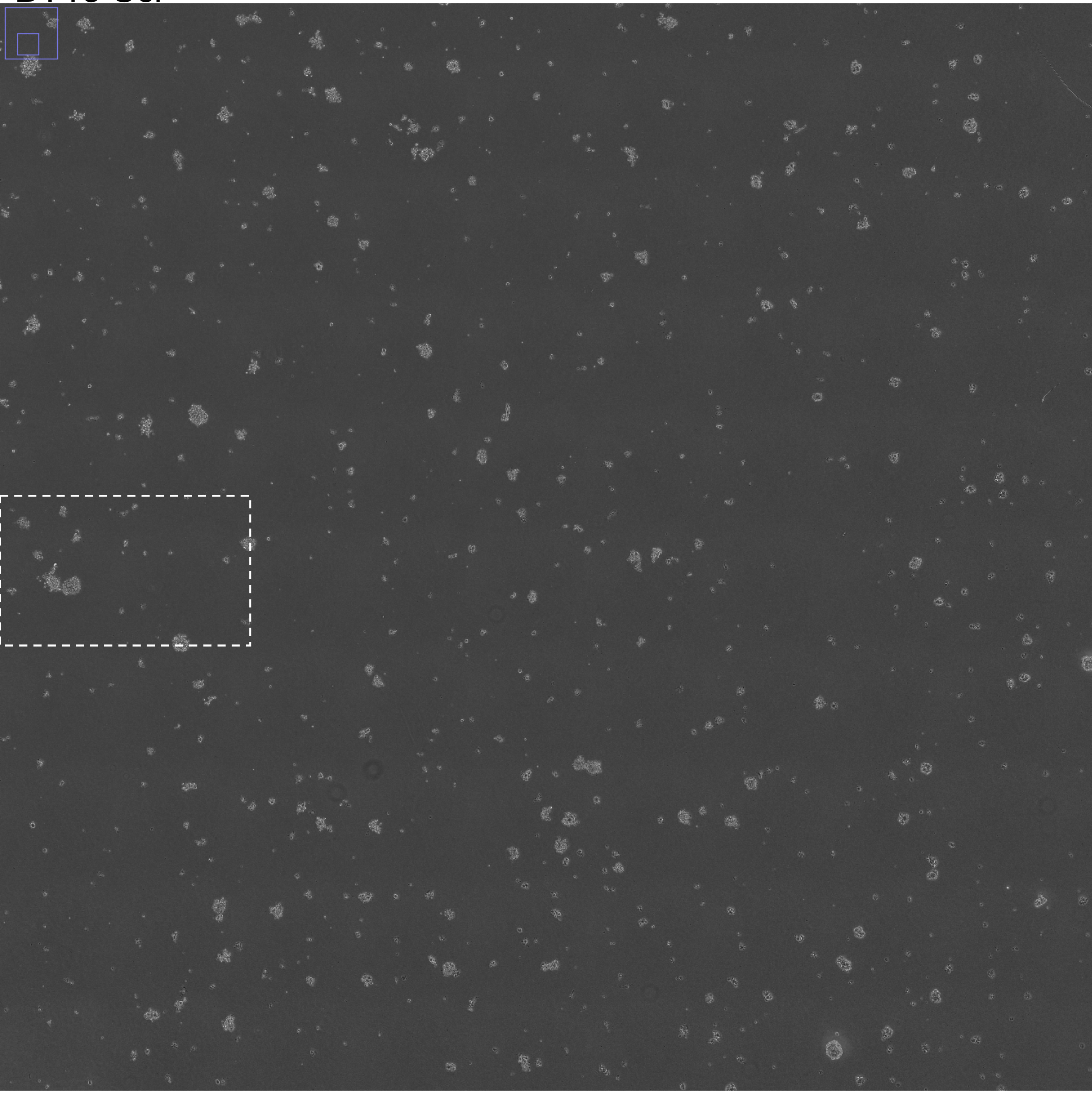

B

BT13 shMDGI1

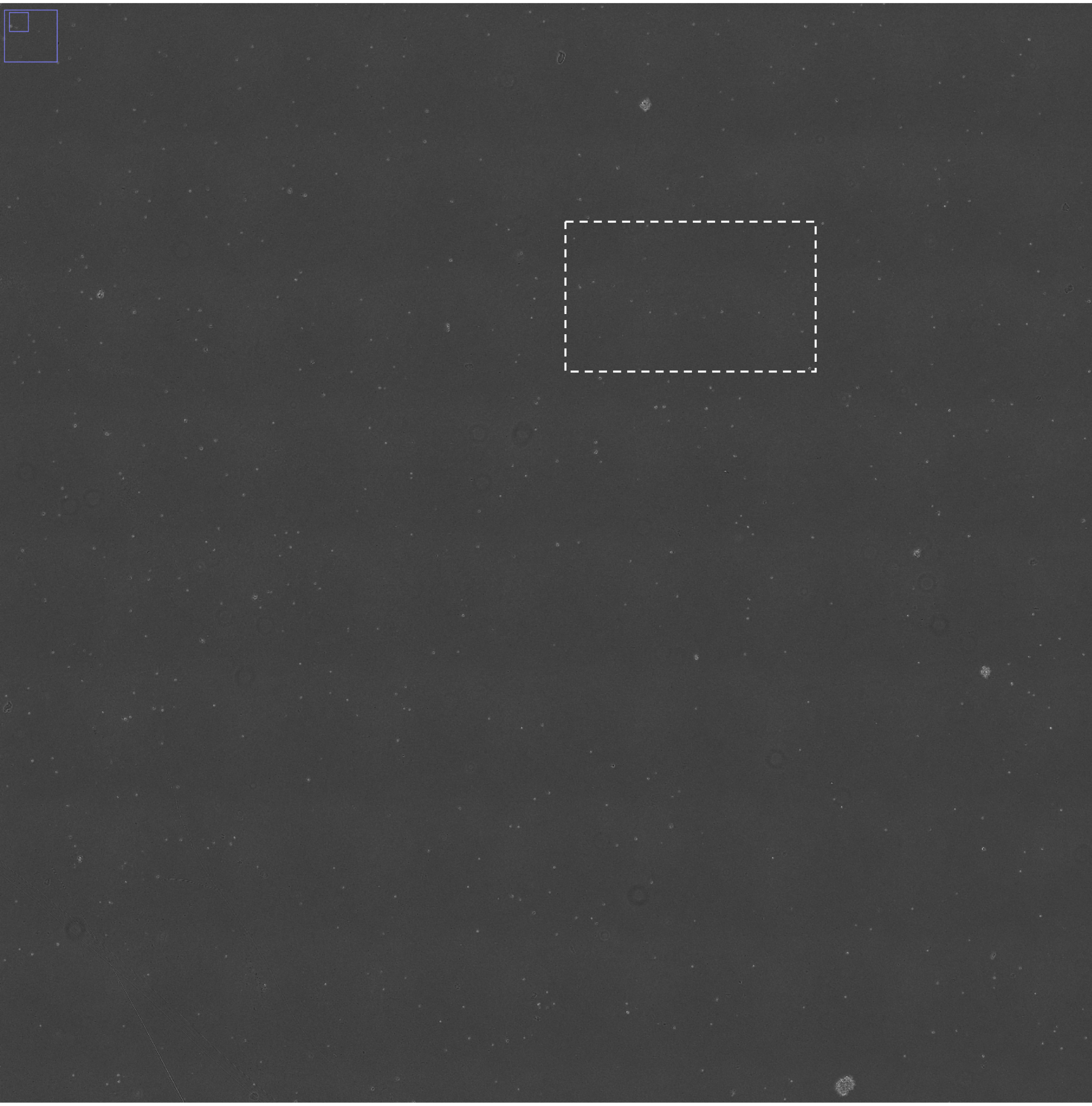

B

BT13 shMDGI2

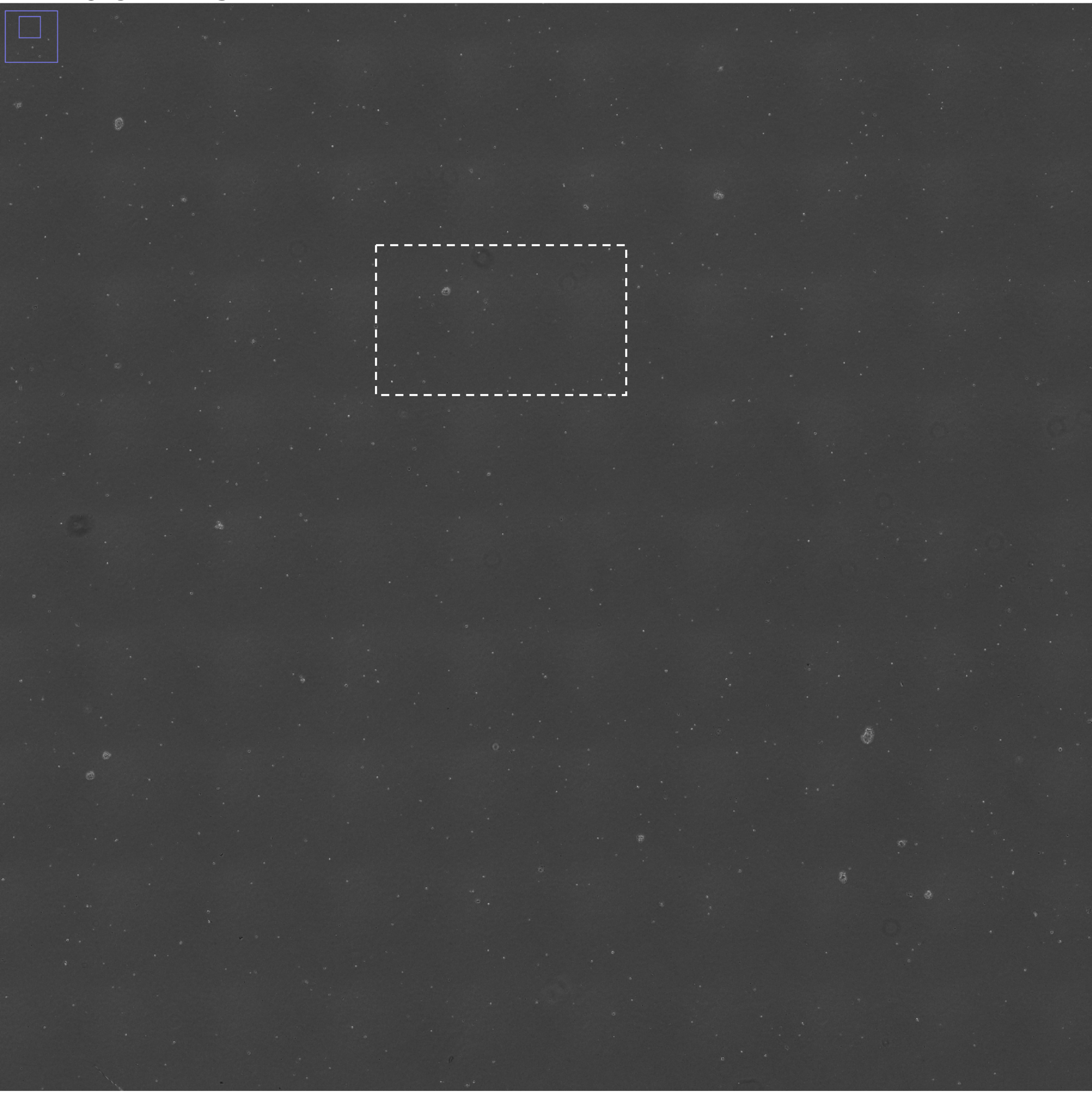

E

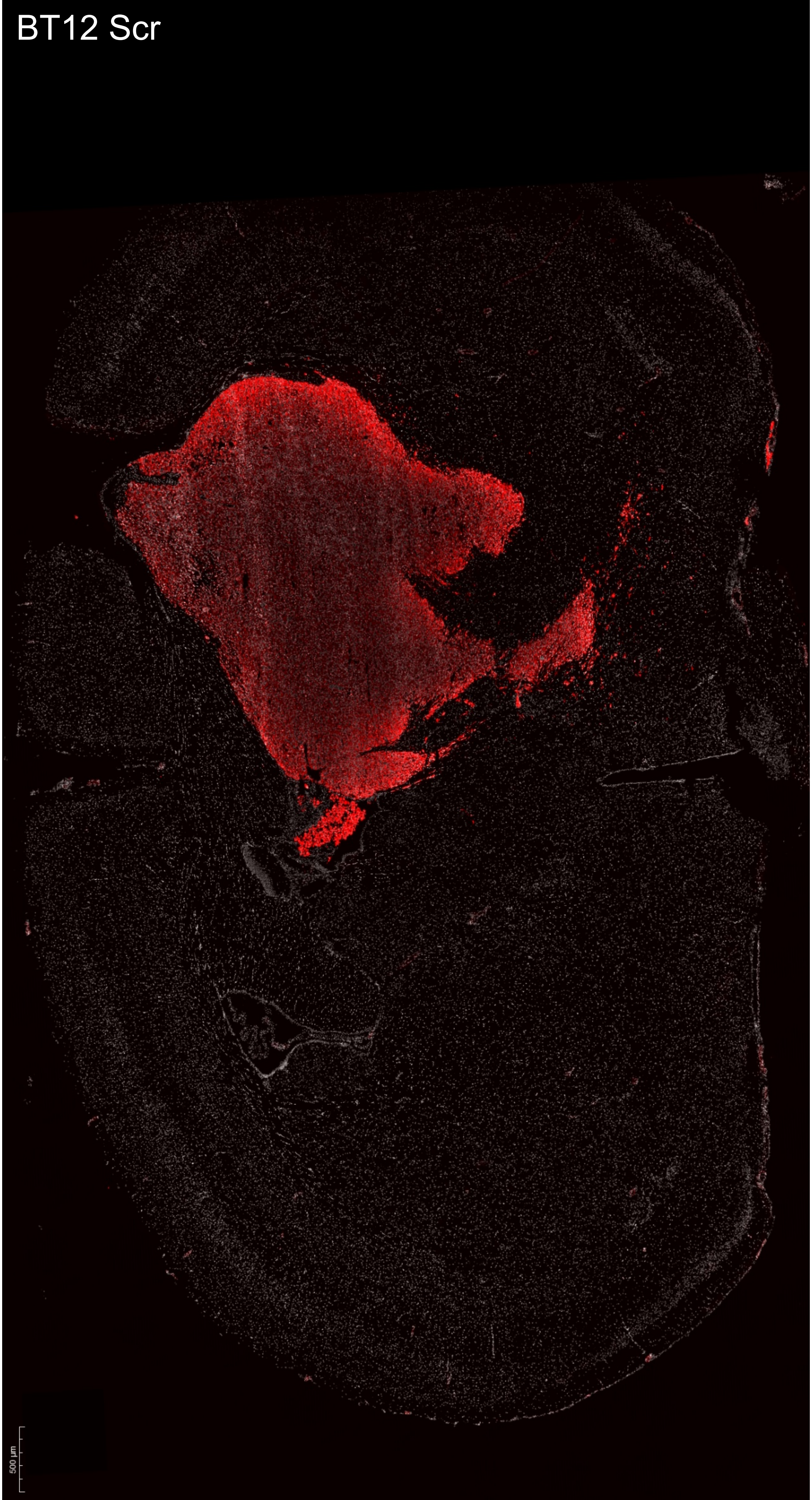

E

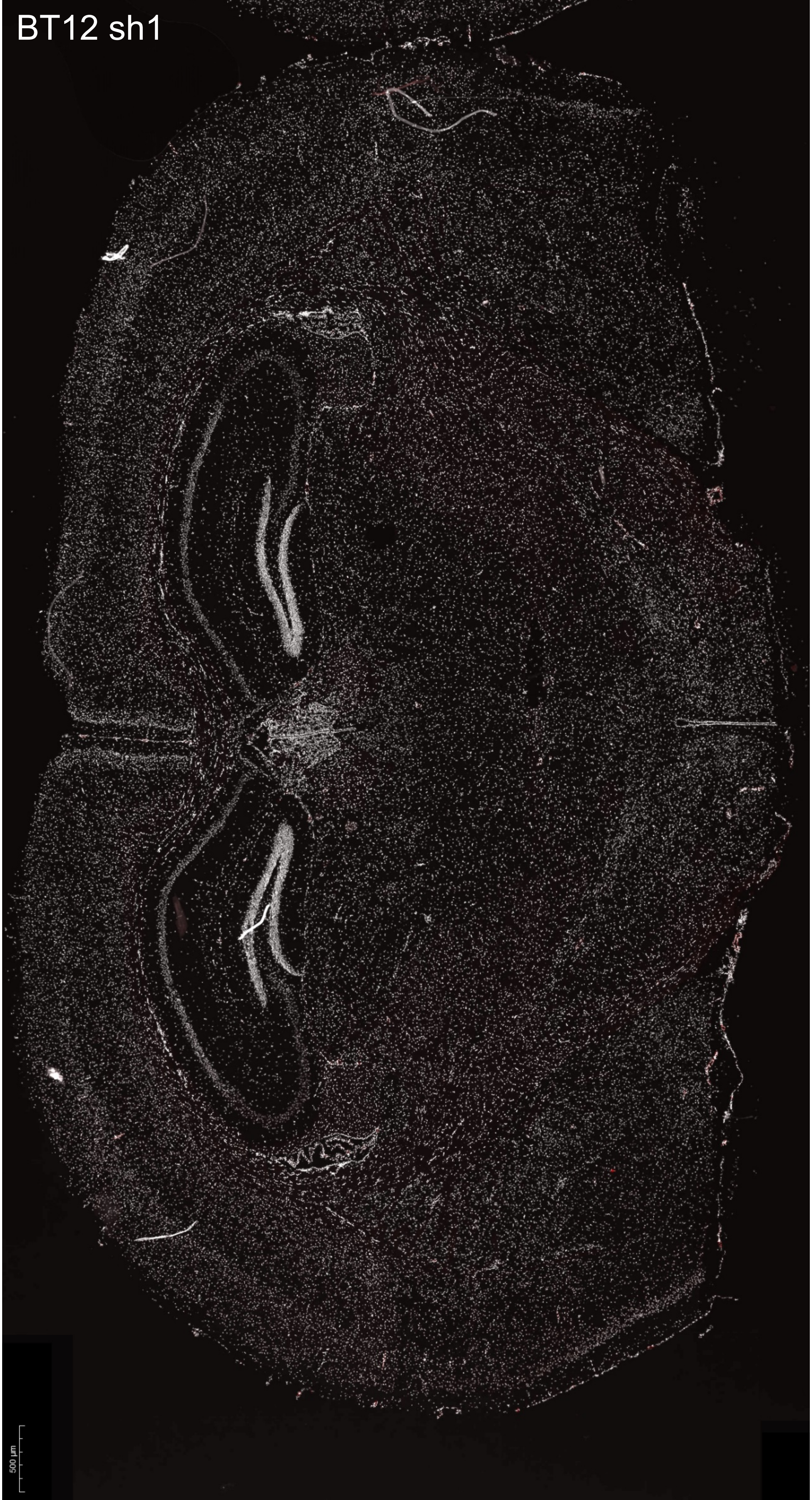

F

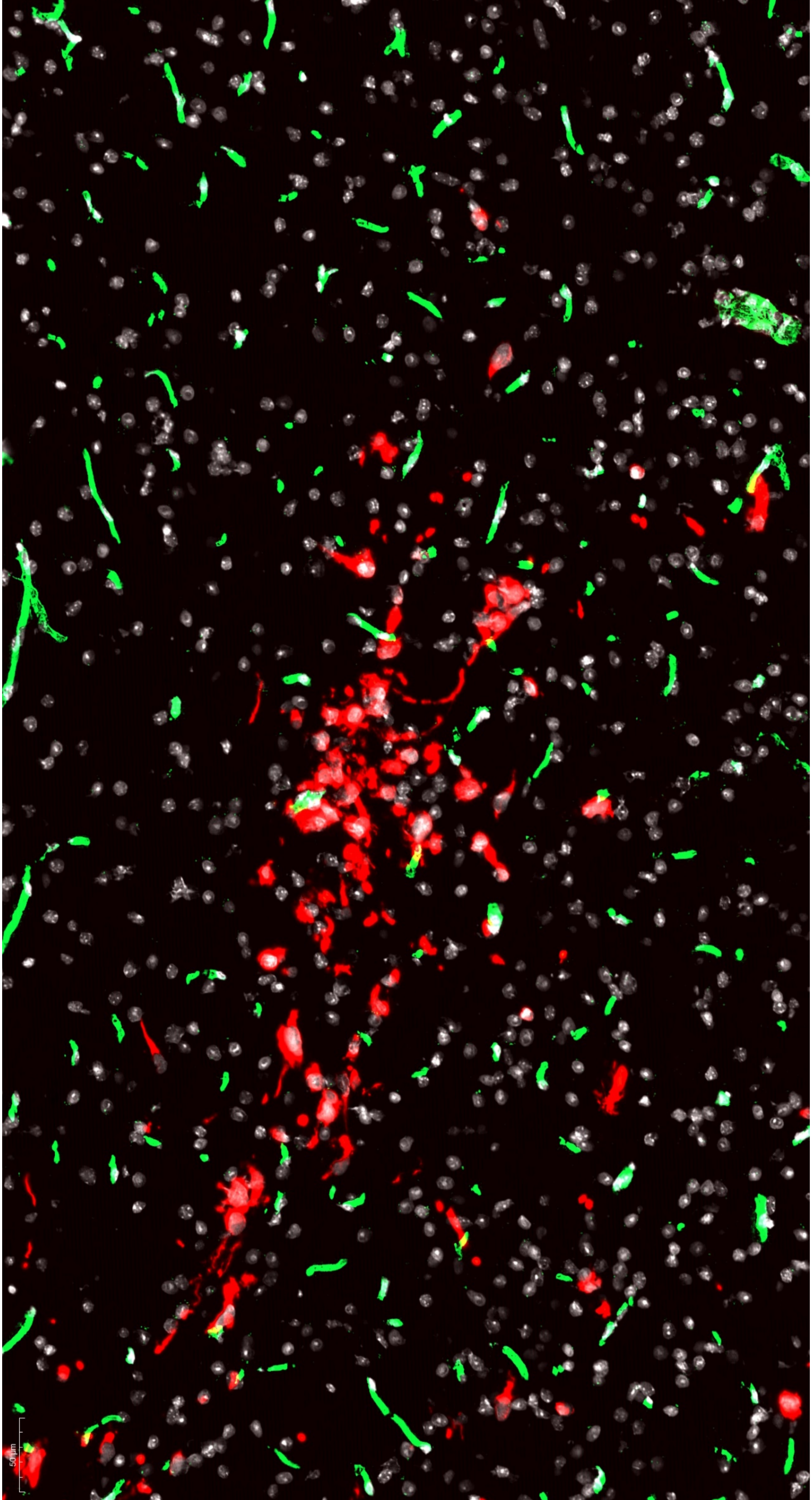

F

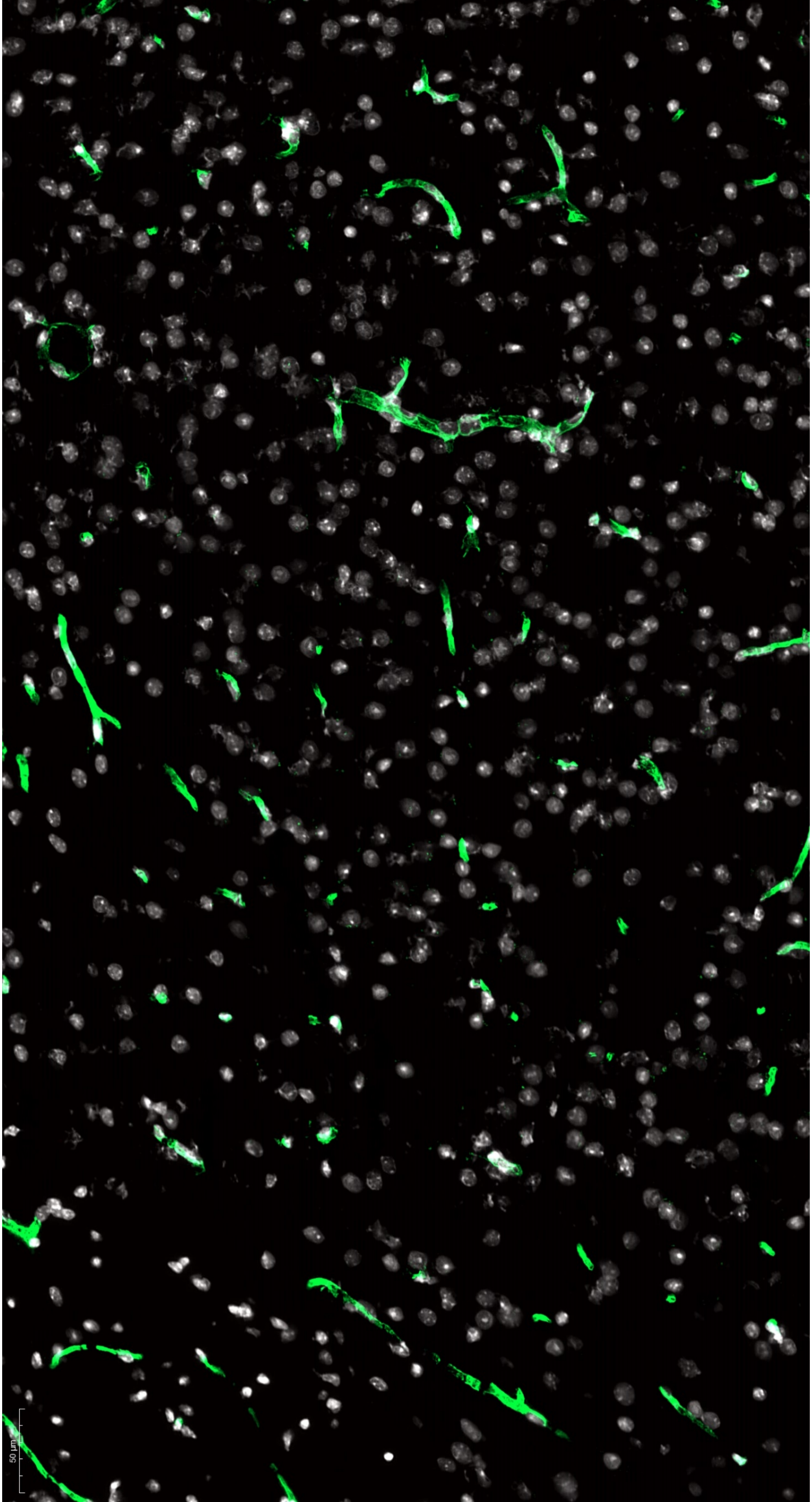

F

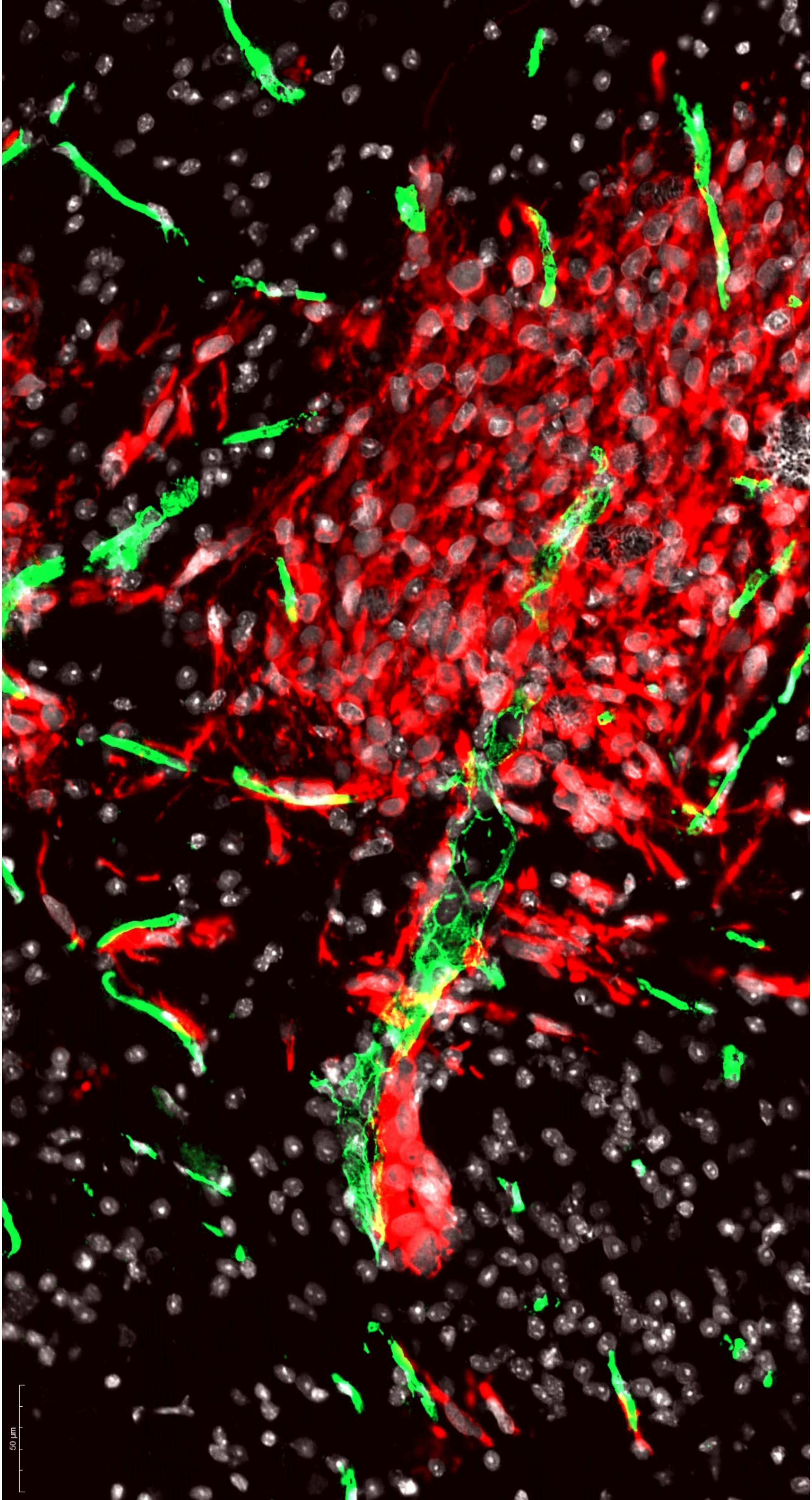

F

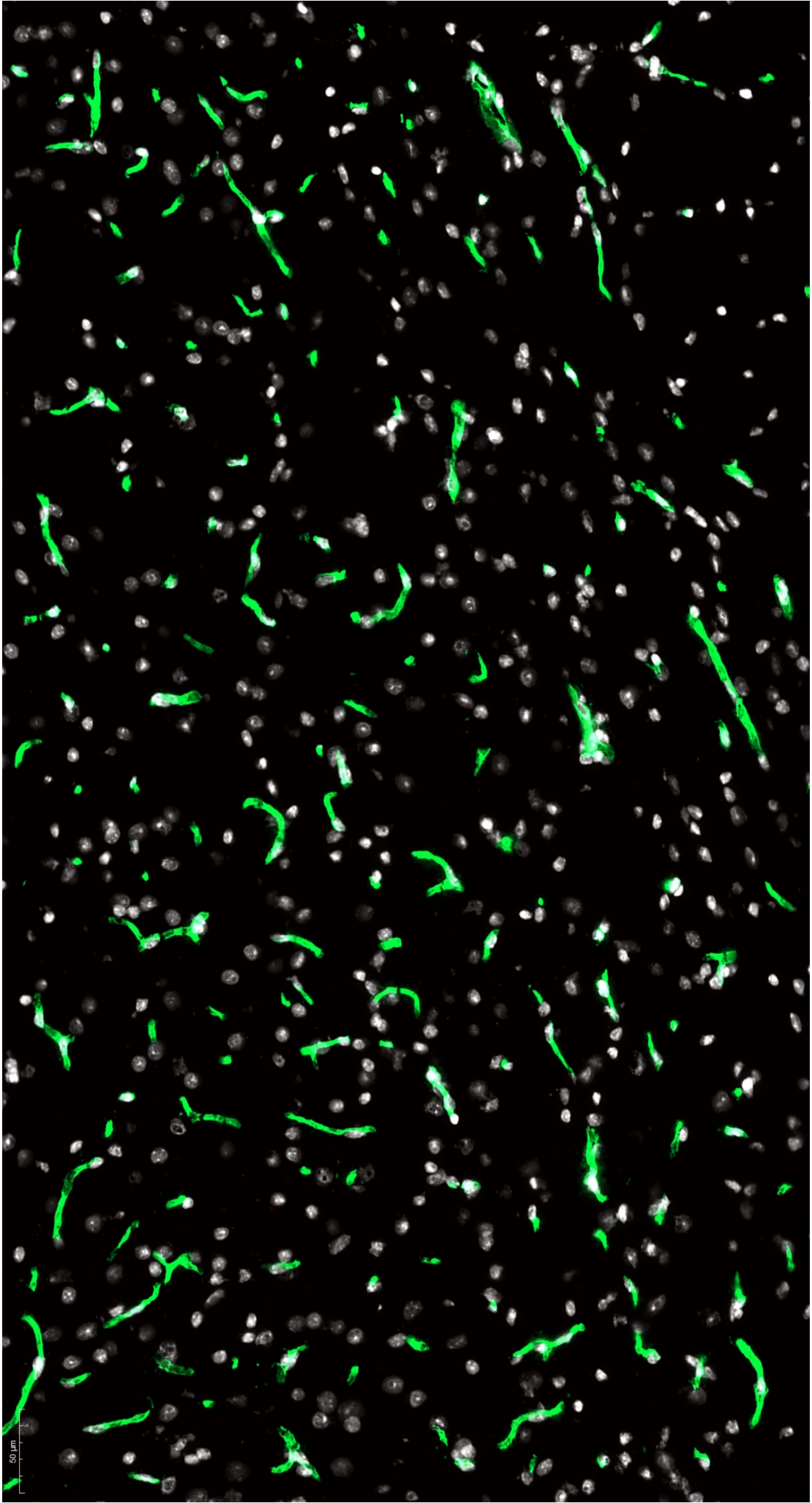

Supplement: Supplementary file 7 — Source Data for Figure 3 [file EMMM-11-e9034-s005.pdf]
